# How Not to Make the Joint Extended Kalman Filter Fail with Unstructured Mechanistic Models

**DOI:** 10.3390/s24020653

**Published:** 2024-01-19

**Authors:** Cristovão Freitas Iglesias, Miodrag Bolic

**Affiliations:** School of Electrical Engineering and Computer Science (EECS), University of Ottawa, Ottawa, ON K1N 6N5, Canada

**Keywords:** joint extended Kalman filter, unstructured mechanistic model, bioprocess monitoring

## Abstract

The unstructured mechanistic model (UMM) allows for modeling the macro-scale of a phenomenon without known mechanisms. This is extremely useful in biomanufacturing because using the UMM for the joint estimation of states and parameters with an extended Kalman filter (JEKF) can enable the real-time monitoring of bioprocesses with unknown mechanisms. However, the UMM commonly used in biomanufacturing contains ordinary differential equations (ODEs) with unshared parameters, weak variables, and weak terms. When such a UMM is coupled with an initial state error covariance matrix P(t=0) and a process error covariance matrix Q with uncorrelated elements, along with just one measured state variable, the joint extended Kalman filter (JEKF) fails to estimate the unshared parameters and state simultaneously. This is because the Kalman gain corresponding to the unshared parameter remains constant and equal to zero. In this work, we formally describe this failure case, present the proof of JEKF failure, and propose an approach called SANTO to side-step this failure case. The SANTO approach consists of adding a quantity to the state error covariance between the measured state variable and unshared parameter in the initial **P**(t = 0) of the matrix Ricatti differential equation to compute the predicted error covariance matrix of the state and prevent the Kalman gain from being zero. Our empirical evaluations using synthetic and real datasets reveal significant improvements: SANTO achieved a reduction in root-mean-square percentage error (RMSPE) of up to approximately 17% compared to the classical JEKF, indicating a substantial enhancement in estimation accuracy.

## 1. Introduction

The extended Kalman filter (EKF) is a recursive Bayesian filter [1,2]. This nonlinear state estimator (NSE) is a commonly used technique for estimating the state of a nonlinear system using a state-space model, first-order linearization, and linear estimation theory. It is composed of a process model and a measurement model along with error covariance matrices of the process (**Q**), measurement (**R**), and state (**P**) [3,4]. The EKF, beyond state estimation, is also used for the parameter estimation (parameter evolution [5]) of nonlinear systems (process models) considering a single joint state variable vector, which includes both the states and parameters of the process model [6,7,8]. This approach is called the **j**oint estimation of states and parameters with an **e**xtended **K**alman **f**ilter (JEKF). The joint estimation problem is motivated by the need to correct the prediction of a process model regarding state variables and to update the process model by evolving its parameters based on the corrections made [8]. A process model should be estimated (evolved) for different conditions of the same application. For example, in biomanufacturing, the parameters of a process model for monitoring a cell culture should change for each new condition. We can use a general set of parameters at the beginning of the process, but we need to evolve them during the process to improve the predictions of the states of the cell culture. Thus, JEKF uses each measurement as soon as it becomes available to correct both the predictions and parameters of a process model [8]. The first discussions and applications of the JEKF approach started in the 1960s for the estimation of linear systems (in which there is a bilinear relation between the states and parameters) [6,7,8,9,10]. However, the JEKF is still very popular, with several new applications in different areas [5,11,12,13,14,15,16,17,18,19,20,21], and with unsolved problems [22,23]. Furthermore, the JEKF has been established as the least expensive nonlinear estimator for moderate-size systems in terms of computational cost because the practical implementation of adaptive controllers using microcontrollers (and/or minicomputers and/or microprocessors) requires numerically economical and robust algorithms, such as the JEKF [11,24]. An important area of application of the JEKF is biomanufacturing, that is, the production of biological products from living cells [20,25,26]. The reason for this is that the JEKF with the mechanistic model (MM) as a process model effectively serves as a soft sensor in biomanufacturing. This combination can enable the real-time monitoring of critical process parameters (CPPs) or critical quality attributes (CQAs) that are difficult to measure directly or that can only be measured at low sampling frequencies in a bioprocess [20,27]. There are two types of MM: structured mechanistic models (SMMs) and unstructured mechanistic models (UMMs) [28]. When we have knowledge about a bioprocess, we can use an SMM with the JEKF. On the other hand, when we do not have knowledge about a bioprocess, we can use a UMM with the JEKF because the UMM allows us to model the macro-scale of a phenomenon. It is a mass-balance equation system with few parameters and variables and less complexity than SMMs [29,30].

The UMM used in biomanufacturing typically consists of ODEs with unshared parameters, weak variables, and weak terms. However, these characteristics of UMM in biomanufacturing, together with the use of **P**(t = 0) and **Q** with uncorrelated elements and the presence of a single measured state variable, represent a failure case that occurs when the JEKF cannot estimate the unshared parameters and the state simultaneously. There are many new bioprocesses for which the literature contains no prior knowledge that the biopharmaceutical industry aims to monitor, such as recombinant adeno-associated virus (rAAV) production [31]. Therefore, enabling the JEKF to side-step the failure case described above may help the industry perform biomanufacturing with the real-time monitoring of bioprocesses with unknown mechanisms. Consequently, this skill can support the biopharmaceutical industry in achieving biomanufacturing 4.0 by becoming more agile and intelligent, thus enhancing product quality, optimizing operations, and reducing costs [25,26,32,33]. Although the biopharmaceutical industry was valued at USD 239.8 billion in 2019 and is estimated to grow at an annual rate of over 13%, it faces significant challenges in achieving the desired productivity and product quality consistently [34].

In this work, we present the common conditions in biomanufacturing that represent a failure case where the JEKF fails to perform the unshared parameter evolution of a UMM, and we propose a solution to side-step this failure case, called SANTO, which consists of a Specific initiAl coNdiTiOn (SANTO) for the matrix Ricatti differential equation (MRDE). Our solution is inspired by the regularization technique to avoid singularity issues in EKF. However, instead of adding a small quantity to the diagonal elements of the state error covariance matrix **P** [35], we only add a quantity to the state error covariance between the measured state variable (MSV) and an unshared parameter (UP) in **P**(t = 0) for the MRDE. The proposed approach can avoid JEKF failure by preventing the Kalman gain from being zero throughout the entire process, which is an unrealistic situation that would mean that the predictions of the UMM (used as a process model) are perfect. Our theoretical and empirical results demonstrate the effectiveness of SANTO, which was assessed using synthetic and real datasets. The code and data used in this work are available in the data availability section of this paper to facilitate reproducibility. Our contributions can be summarized as follows:

- We provide proof of JEKF failure when acting as an unshared parameter estimator under specific biomanufacturing conditions that represent a failure case. To our knowledge, this is the first work to formally report this failure case regarding the JEKF.
- An approach to avoid the JEKF failure that enables using JEKF with UMM for real-time bioprocess monitoring. This is helpful in the macro-scale modeling of a phenomenon with UMM where the underlying process mechanism is not fully understood.

## 2. Related Work

In contrast to JEKF, the dual extended Kalman filter (DEKF) employs two consecutive EKFs, separating the estimation of system states and parameters [36]. This separation can be advantageous in certain scenarios, but JEKF offers three important benefits, particularly in the context of the practical implementation of adaptive controllers using microcontrollers in biomanufacturing that requires numerically economical and robust algorithms such as JEKF [11,24]. First, JEKF avoids the computational overhead associated with running two separate filters, as in DEKF, enhancing computational efficiency [37]. Second, it can provide more accurate and robust estimates in scenarios, such as nonlinear biochemical systems, that commonly occur in biomanufacturing processes [36]. Lastly, the single-filter structure of JEKF is simpler to implement and tune compared to the dual-filter approach of DEKF [8]. The main limitation of JEKF is not guaranteed convergence in some cases, as reported by [6,24,38]. A solution to deal with the convergence problems of JEKF is to use recurrent derivatives [6,38]. However, a theoretical justification for that was not provided [8]. On the other hand, it was reported that the cause of divergence in JEKF is linked to the linearization of the coupled system and not due to the lack of recurrent derivatives [24]. Furthermore, there are certain cases where the JEKF may be unable to estimate the parameters and the state simultaneously, such as singularity issues [35]. However, until now, the failure case (biomanufacturing conditions) where JEKF fails as an unshared parameter estimator has not been formally reported. Recently, the JEKF was applied for monitoring rAAV production [19]. In developing this application, the authors dealt with a situation that resembles the failure case reported here. Because they reported the use of a simple UMM, **P**(t = 0), and **Q** with uncorrelated elements and a second linear operator as an approach to enable Kalman gain (**K**) and **P** to be updated with prior error covariances with regard to the UMM parameters, their results showed the unshared parameter evolution with convergence. However, the authors did not describe the problem in detail. They did not present a theoretical justification for the approach used (second linear operator). They clearly stated that the work is an initial study and reported the need for future validation. We named this approach KPH2 because the authors used a second linear operator to enable **K** and **P** to be updated, and we used this approach in our experimental evaluation for comparison purposes with our proposed approach. A description of KPH2 and a possible interpretation can be found in Section S6 of the Supplementary Material.

## 3. Background

### 3.1. Unstructured Mechanistic Model (UMM)

Unstructured Mechanistic Models (or Unstructured Mechanistic Kinetic Models) are models of the temporal evolution of a bioprocess [39]. They are based on first-principle mechanisms that drive the bioprocess under consideration [34]. Examples of bioprocesses are (i) the production of therapeutic monoclonal antibodies (mAbs), which is projected to bring in USD 300 billion by 2025 [34], and (ii) the rAAV production that is a viral vector technology for gene therapy considered the safest and most effective way to repair single-gene abnormalities in non-dividing cells [19,31]. It is essential to point out that despite UMM being the most suitable option to describe the dynamic behavior of bioprocesses and being considered a crucial foundation for soft sensors in DT development, its industrial use is still in its early stages [28,39,40]. The UMMs are important because they allow for the macro-scale modeling of the bioreactor’s functionality and can provide insight into the upstream process’s underlying macro-scale phenomena. For example, this kind of model can be used to depict the dynamics of the cell density, viability, nutrient/metabolite concentrations, and product titer [41,42,43]. Therefore, UMMs are the most suitable option for explaining observed phenomena, predicting process behavior, and analyzing intrinsic bioprocess characteristics such as controllability [34].

The main difference between UMM and SMM is that SMM is more complex than UMM because it provides details about the intracellular environment of a homogenous cell population. Therefore, the development of SMM for a specific bioprocess requires extensive domain knowledge and substantial effort [34,41]. SMM is unsuitable for the dynamic control of bioprocess in bioreactors used commonly in biomanufacturing because many of the variables used in SMM cannot be manipulated directly [34]. SMM is most suited for cell-line development, in which a cells’ genome-level properties are changed to produce the desired process behavior [34].

It is essential to point out that a simple UMM has limited predictive power and is insufficient to process state estimation. Moreover, it is improbable that a single set of parameter values enables a kinetic model to satisfy several datasets collected under distinct operating circumstances [44]. The Kalman filter approach is commonly implemented with UMM [45] to improve prediction accuracy and generate predictions between sampling instances. Among several data analysis methods, the Kalman filter and its nonlinear extensions, such as the extended Kalman filter, are effective tools for predicting the values of unobserved states. Examples of UMM used in biomanufacturing can be found in Section S1 of Supplementary Material.

### 3.2. Continuous-Discrete Extended Kalman Filter

This section gives an overview of the continuous-discrete EKF (CD-EKF) algorithm. A detailed description of CD-EKF can be found in Section S2 of the Supplementary Material. The EKF requires a state-space model to perform an estimation on the state variables of a process (nonlinear system) present in a state variable vector ψ(t) [1,36,44]. A state-space model consists of process and measurement (observation) models [46]. EKF linearizes the nonlinear system (state-space model) by calculating the Jacobians of the nonlinear process and measurement models based on the first-order Taylor series expansion in order to analytically propagate the Gaussian random-variable representation [8,20,44].

A UMM can be used as the process model of EKF. The state variables vector to be used by the EKF is composed of the state variables of the UMM (observed and unobserved), and the state variables vector is defined as:

(1) $$\psi \left(t\right)={\left[x_{1},x_{2},\ldots ,x_{n}\right]}^{T}.$$

Subsequently, the process model is represented as

(2) $$\frac{d\psi (t)}{dt}=\phi \left(\psi \left(t\right),t,\theta \right)+\omega \left(t\right),$$

where ϕ denotes nonlinear functions of the state variables in ψ(t) , which corresponds to a UMM. The process model is formulated in a continuous time *t*, and the white process noise vector is represented by ω∼N(0,Q) with the zero mean and the error covariance matrix of process model represented by Q .

The measurement model is treated as a discrete system and defined as

(3) $$Z_{k}=h\left(\psi \left(t_{k}\right)\right)+v.$$

The nonlinear function *h* in the measurement model relates the current state variables to the measurements $Z_{k}$ . The white measurement noise vector is represented by v∼N(0,R) with zero mean and measurement noise variance represented by R . When some state variables can be measured directly, we have a simple case and *h* can be a linear model. If *h* is linear, we have $h\left(\psi \left(t_{k}\right)\right)=H\psi \left(t_{k}\right)$ [20,36,47] where the matrix H is a linear operator (row vector) that matches the states variables of $\psi (t_{k})$ to the measured variables $Z_{k}$ that are obtained at a discrete instance *k* [20,47]. Consequently, the measurement model (3) can be rewritten as

(4) $$Z_{k}=H\psi \left(t_{k}\right)+v.$$

The EKF algorithm is implemented through a state variables vector ψ(t) , initial condition, prediction step (time update) and correction step (measurement update) [1,20,21,36,47].

*Initialization step:* The initial condition is composed of the initial mean ${\hat{\psi }}_{0}=E\left[\psi_{0}\right]$ and initial error covariance matrix $P_{0}=P\left(t=0\right)=E\left[\left(\psi_{0}-{\hat{\psi }}_{0}\right){\left(\psi_{0}-{\hat{\psi }}_{0}\right)}^{T}\right]$ of the state variables vector in addition to the error covariance matrices of the process **Q** and measurement **R** [8].

*Prediction step:* In this step, the a priori predictions represented by the predicted mean $\hat{\psi }\left(t_{k/k-1}\right)$ and predicted error covariance matrix $P(t_{k|k-1})$ of state variables vector ψ(t) are obtained. This is completed by numerically integrating ϕ(ψ(t),t,θ) from discrete time $t_{k-1}$ to $t_{k}$ the following equation

(5) $$\hat{\psi }\left(t_{k/k-1}\right)={\left.\hat{\psi }\left(t_{k-1}\right)+\int_{t_{k-1}}^{t_{k}}\phi \left(\hat{\psi }\left(t\right)\right)dt\right|}_{\hat{\psi }\left(t_{k-1}\right)}$$

and solving the MRDE to predict the state error covariance matrix [4,48]

(6) $$\frac{dP(t)}{dt}=J_{t}^{\phi }P\left(t\right)+P\left(t\right)J_{t}^{\phi T}+Q$$

from $t_{k-1}$ to $t_{k}$ , where a new measurement is obtained at time k [4,49], and $J_{t}^{\phi }$ is the Jacobian matrix of ϕ evaluated at the prior mode [50,51],

(7) $$J_{t}^{\phi }={\left.\frac{\partial \phi (\psi (t))}{\partial \psi_{i}}\right|}_{\psi \left(t\right)=\hat{\psi }\left(t-1\right)}.$$

Equation (6) is basically a matrix of ODEs, and the matrix of ODEs solutions obtained from $t_{k-1}$ to $t_{k}$ represent each error covariance of the system state.

*Correction step:* In this step, the results of the prediction step ( $\hat{\psi }\left(t_{k/k-1}\right)$ and $P(t_{k|k-1})$ ) are combined with the measured value $Z_{k}$ and the Kalman gain ( $K_{k}$ ) to provide the estimated mean $\hat{\psi }\left(t_{k/k}\right)$ and estimated error covariance matrix $P(t_{k|k})$ of state variables using the following equations:

(i) innovation equations

(8) $$e_{Z,k}=Z_{k}-H\hat{x}\left(t_{k/k-1}\right)$$

(9) $$S_{k}=HP\left(t_{k|k-1}\right)H^{T}+R$$

and (ii) update step equations

(10) $$K_{k}=P\left(t_{k|k-1}\right)H^{T}S_{k}^{-1}$$

(11) $$\hat{x}\left(t_{k/k}\right)=\hat{x}\left(t_{k/k-1}\right)+K_{k}e_{Z,k}$$

(12) $$P\left(t_{k|k}\right)=\left(I-K_{k}H\right)P\left(t_{k|k-1}\right)$$

where $e_{Z,k}$ and $S_{k}$ represent, respectively, the innovation error and innovation covariance.

The Kalman gain is a scaling factor (ratio) to estimate the state variables by setting a value between the predicted state and measured state [4,50]. The $K_{k}$ chooses a value along the residual range ( $Z_{k}$ - $H\hat{\psi }\left(t_{k/k-1}\right)$ ) [8,50]. $K_{k}$ enables to set a value for $\hat{\psi }\left(t_{k/k}\right)$ between the $\hat{\psi }\left(t_{k/k-1}\right)$ (prediction) and $Z_{k}$ (measurement) using Equation (11) and update the belief regarding the state variables based on how certain we are regarding the measurement using Equation (12) [50]. The Kalman gain is computed as a ratio of prior and measurement uncertainty available; see Equation (10). The one-dimensional form of Equation (10) is the following K=P/(P+R) [50]. It is important to point out that linear operator H matches the states variables of $\psi (t_{k})$ to the measured variables $Z_{k}$ that are obtained at a discrete instance.

Using the estimated mean $\hat{\psi }\left(t_{k/k}\right)$ and the estimated error covariance matrix $P(t_{k|k})$ of the vector of the state variables as an initial condition, we can return to the prediction step until the next measurement is obtained and everything repeated again.

### 3.3. JEKF

JEKF is a Bayesian filter-based joint estimation approach where the states $x_{i}$ and parameters θ of a process model are concatenated into a single joint state vector [52]. Then, the state variables vector ( $\psi \left(t\right)={\left[x_{1},x_{2},\ldots ,x_{n}\right]}^{T}$ ) is considered as extended/augmented as following,

(13) $$\psi \left(t\right)={\left[x_{1},x_{2},\ldots ,x_{n},\theta_{1},\ldots ,\theta_{n}\right]}^{T}.$$

To be more specific, we consider the problem of learning both the states $x_{i}$ and parameters $\theta_{i}$ of a discrete-time nonlinear dynamical system (such as the UMM described in Section S1 of Supplementary Material) that is used as a process model. In JEKF, the system states $x_{i}$ and the set of model parameters $\theta_{i}$ for the dynamical system are simultaneously corrected based only on the observed noisy signal $Z_{k}$ . It is essential to point out that we consider JEKF as an approach for parameter evolution [5], because it cannot guarantee convergence in some cases [6]. However, it can guarantee the evolution of the parameters based on the following equation [5]

(14) $$\theta \left(t_{k}\right)=\theta \left(t_{k-1}\right)+noise,$$

where the parameters are defined as random variables with perturbation (noise) added at each time step. This parameter evolution can be enough to update the process model parameters when we are near the optimal parameters regarding a specific condition. In this paper, when we say parameter estimation, we are referring to parameter evolution.

## 4. Theoretical Analysis

This section presents the theoretical analysis of the JEKF failure to perform unshared parameter evolution with a UMM and SANTO, which is the proposed solution for this problem.

### 4.1. JEKF Failure

First, we present the conditions where JEKF fails to estimate (parameter evolution) the unshared parameters of a UMM. Next, we present the theoretical proof of the failure. However, before starting the analysis, we formally define unshared parameters and weak and strong terms/variables of an ODE as follows:

- **Unshared parameters:** They are parameters used only in one term of an ODE and not used by other ODEs of the same UMM. See the example in Section S3.1 of the Supplementary Material.
- **Weak and Strong term of an ODE:** A *weak term* is a term of an ODE with a low percentage of variables of the state variable vector, and a *"strong term"* is one with a high percentage of variables of the state variable vector. See the example in Section S3.2 of the Supplementary Material.
- **Weak and Strong variable of an ODE:** A *weak variable* is a variable used only in the first member of an ODE in UMM, and a *strong variable* is a variable used in the first member and different terms of the second member of an ODE. Furthermore, it is used in the second member of other ODEs of the same UMM. See the example in Section S3.3 of the Supplementary Material.

#### 4.1.1. Failure Case: Biomanufacturing Conditions

The following conditions are prevalent in biomanufacturing and should be taken into consideration while developing JEKF applications for this area:

- **ODEs of UMM with unshared parameters.** This parameter type is commonly used in ODE to model the dynamic of product formation in biomanufacturing [53,54,55]. See the example in Section S3.1 of the Supplementary Material.
- **P and Q with uncorrelated elements.** In case of the limited amount of data, it is very common to assume **P** and **Q** with uncorrelated elements in EKF applications [19,20,21,47]. This assumption means that the error covariance matrices **P** and **Q** are diagonal, with the diagonal elements being the noise variances (P ${\text{​}}_{i,i}\neq 0$ and Q ${\text{​}}_{i,i}\neq 0$ ) and off-diagonal elements equal to zero (P ${\text{​}}_{i,j}=0$ and Q ${\text{​}}_{i,j}=0$ ). The **Q** constant and with uncorrelated elements is used only to build the MRDE, and the **P** with uncorrelated elements can be used to build an MRDE and as an initial condition of MRDE (the initial predicted state error covariance **P**(t = 0)).
  This assumption raises two scenarios:
  1. The use of **P** with uncorrelated elements to build the MRDE (Equation (6)) and **P**(t = 0) with uncorrelated elements as the initial condition. When **P** with uncorrelated elements is used to build the MRDE, the ODEs of MRDE are based only on noise variance of P ${\text{​}}_{i,i}$ and Q ${\text{​}}_{i,i}$ and elements of Jacobian $J_{t}^{\phi }$ . See the example in Section S3.4 of the Supplementary Material. It is important to point out that depending on the partial derivative, the ODE to predict a state error covariance can be time-invariant $\frac{dP_{i,j}\left(t_{k|k-1}\right)}{dt}=0$ . See Section S3.2 of the Supplementary Material.
  2. The use of **P** with correlated elements to build the MRDE (Equation (6)) and **P**(t = 0) with uncorrelated elements as the initial condition. This means that the ODE of MRDE can be composed of off-diagonal elements of **P**, and it can reduce the number of the time-invariant ODE to predict a state error covariance between two state variables.
- **ODEs of UMM with weak terms.** A strong term contributes more than a weak term to compute the predicted state error covariance $P(t_{k|k-1})$ . Many elements of Jacobian $J_{t}^{\phi }$ result from the partial derivation of a strong term. See the example in Section S3.2 of the Supplementary Material.
- **ODEs of UMM with weak variables.** In the Jacobian $J_{t}^{\phi }$ , the first-order partial derivatives of all functions with respect to a *weak variable* are equal to zero. Consequently, this variable type does not contribute to the calculations of predicted error covariance $P(t_{k|k-1})$ since it will not be part of any element of MRDE to predict the state error covariance matrix $P(t_{k|k-1})$ . On the other hand, a *strong variable* contributes to the calculations of predicted error covariance $P(t_{k|k-1})$ . See the example in Section S3.3 of Supplementary Material.
- **Only one measured state variable.** In some cases (JEKF application), measuring only one state variable is possible. This measured state variable determines which column of the predicted state error covariance $P(t_{k|k-1})$ is used to compute the Kalman gain through $P\left(t_{k|k-1}\right)H^{T}$ in Equation (10). If this column has a row with a value equal to zero (no covariance between the measured variable and state variable represented by the row), the Kalman gain cannot be computed to the state variable defined by the row. See the example in Section S3.5 of the Supplementary Material.

#### 4.1.2. Lemma: Inability to Update Kalman Gain for Unshared Parameters based **P**(t = 0) and **Q** with Uncorrelated Elements

Given the conditions described above, we have the following Lemma:

**Lemma** **1.** *The Kalman gain cannot be updated (by Equation (10)) for an unshared parameter that is part of a state variable vector and part of a weak term in a UMM if the initial state error covariance matrix* ***P**(t = 0) and* ***Q*** *are formed by uncorrelated elements and there is only one state variable measured.*

The proof of this lemma is in the following, and an example can be found in Section S4 of the Supplementary Material.

**Proof of Lemma** **1.** Let us consider the following:

- A general UMM with an unshared parameter in a weak term represented by a system of nonlinear differential equations of the form:
(15) $$\begin{array}{cc} \frac{dx_{msv}}{dt} & =f_{1}\left(x_{msv},x_{2},\ldots ,x_{n-1},\theta_{1},\theta_{2},\ldots ,\theta_{m}\right) \end{array}$$
(16) $$\begin{array}{cc} \frac{dx_{2}}{dt} & =f_{2}\left(x_{msv},x_{2},\ldots ,x_{n-1},\theta_{1},\theta_{2},\ldots ,\theta_{m}\right) \end{array}$$
(17) $$\begin{array}{cc} \; & \vdots \end{array}$$
(18) $$\begin{array}{cc} \frac{dx_{n}}{dt} & =f_{n}\left(x_{msv},\theta_{up}\right) \end{array}$$
  where $x_{msv}$ and $x_{2},\ldots ,x_{n}$ are the variables of the system, $f_{1},f_{2},\ldots ,f_{n}$ are the functions defining the system, and $\theta_{1},\theta_{2},\ldots ,\theta_{m}$ are the parameters of the system, and $\theta_{up}$ is an unshared parameter.
- A joint state variables vector defined as
(19) $$\psi {\left(t\right)}_{general}=\left[x_{msv},x_{2},\ldots ,x_{n},\theta_{up}\right].$$
- A process model defined as
(20) $$\frac{d\psi {\left(t\right)}_{general}}{dt}=\phi \left(\psi {\left(t\right)}_{general},t\right)+\omega \left(t\right)=\frac{d}{dt}\left[\begin{array}{c} x_{msv}\\ x_{2}\\ \vdots \\ x_{n}\\ \theta_{up} \end{array}\right]=\left[\begin{array}{c} f_{1}\\ f_{2}\\ \vdots \\ f_{n}\\ 0 \end{array}\right]+\omega \left(t\right).$$
-
$x_{msv}$ as the unique measured state variable (MSV) and H = [1 0 … 0 0].
- R as measurement noise variance of $x_{msv}$ .
-
$\theta_{up}$ as the unshared parameter (UP) to be evolved (estimated) and presented in only one weak term.
- **P** and **Q** with uncorrelated elements for the $\psi {\left(t\right)}_{general}$ (Equation (19)),
(21) $$P=\left[\begin{array}{ccccc} P_{x_{msv},x_{msv}} & 0 & \cdots & 0 & 0\\ 0 & P_{x_{2},x_{2}} & \cdots & 0 & 0\\ \vdots & \vdots & \ddots & \vdots & \vdots \\ 0 & 0 & \cdots & P_{n,n} & 0\\ 0 & 0 & \cdots & 0 & P_{\theta_{up},\theta_{up}} \end{array}\right],$$
(22) $$Q=\left[\begin{array}{ccccc} Q_{x_{msv},x_{msv}} & 0 & \cdots & 0 & 0\\ 0 & Q_{x_{2},x_{2}} & \cdots & 0 & 0\\ \vdots & \vdots & \ddots & \vdots & \vdots \\ 0 & 0 & \cdots & Q_{n,n} & 0\\ 0 & 0 & \cdots & 0 & Q_{\theta_{up},\theta_{up}} \end{array}\right].$$
- The Jacobian $J_{t}^{\phi }$ (Equation (7)), with the $\psi {\left(t\right)}_{general}$ (Equation (19)),
(23) $$J_{t}^{\phi }\left(\phi \left(\psi {\left(t\right)}_{general},t\right)\right)=\left[\begin{array}{ccccc} \frac{\partial f_{1}}{\partial x_{msv}} & \frac{\partial f_{1}}{\partial x_{2}} & \cdots & \frac{\partial f_{1}}{\partial x_{n}} & 0\\ \frac{\partial f_{2}}{\partial x_{msv}} & \frac{\partial f_{2}}{\partial x_{2}} & \cdots & \frac{\partial f_{2}}{\partial x_{n}} & 0\\ \vdots & \vdots & \ddots & \vdots & \vdots \\ \frac{\partial f_{n}}{\partial x_{msv}} & \frac{\partial f_{n}}{\partial x_{2}} & \cdots & \frac{\partial f_{n}}{\partial x_{n}} & \frac{\partial f_{n}}{\partial \theta_{up}}\\ 0 & 0 & \cdots & 0 & 0 \end{array}\right].$$

Given these conditions and Equation (6), we have the following MRDE (based on **P** uncorrelated)

(24) $$\frac{dP(t)}{dt}=\left[\begin{array}{ccccc} \frac{dP_{x_{msv},x_{msv}}\left(t\right)}{dt}=Q_{1,1}+2P_{1,1}\frac{\partial f_{1}}{\partial x_{msv}} & \frac{dP_{x_{2},x_{msv}}\left(t\right)}{dt}=\left(P_{1,1}+P_{2,2}\right)\frac{\partial f_{1}}{\partial x_{2}} & \cdots & \frac{dP_{x_{n},x_{msv}}\left(t\right)}{dt}=\left(P_{1,1}+P_{3,3}\right)\frac{\partial f_{1}}{\partial x_{n}} & \frac{dP_{\theta_{up},x_{msv}}\left(t\right)}{dt}0\\ \frac{dP_{x_{msv},x_{2}}\left(t\right)}{dt}=\left(P_{1,1}+P_{2,2}\right)\frac{\partial f_{2}}{\partial x_{msv}} & \frac{dP_{x_{2},x_{2}}\left(t\right)}{dt}=Q_{2,2}+2P_{2,2}\frac{\partial f_{2}}{\partial x_{2}} & \cdots & \frac{dP_{x_{n},x_{2}}\left(t\right)}{dt}=\left(P_{3,3}+P_{2,2}\right)\frac{\partial f_{2}}{\partial x_{n}} & \frac{dP_{\theta_{up},x_{2}}\left(t\right)}{dt}=0\\ \vdots & \vdots & \ddots & \vdots & \vdots \\ \frac{dP_{x_{msv},x_{n}}\left(t\right)}{dt}=\left(P_{1,1}+P_{n,n}\right)\frac{\partial f_{n}}{\partial x_{msv}} & \frac{dP_{x_{2},x_{n}}\left(t\right)}{dt}=\left(P_{2,2}+P_{n,n}\right)\frac{\partial f_{n}}{\partial x_{2}} & \cdots & \frac{dP_{x_{n},x_{n}}\left(t\right)}{dt}=Q_{n,n}+2P_{n,n}\frac{\partial f_{n}}{\partial x_{n}} & \frac{dP_{\theta_{up},x_{n}}\left(t\right)}{dt}=0\\ \frac{dP_{x_{msv},\theta_{up}}\left(t\right)}{dt}=0 & \frac{dP_{x_{2},\theta_{up}}\left(t\right)}{dt}=0 & \cdots & \frac{dP_{x_{n},\theta_{up}}\left(t\right)}{dt}=0 & \frac{dP_{\theta_{up},\theta_{up}}\left(t\right)}{dt}=0 \end{array}\right].$$

Now, using this Equation (24) to compute the predicted state error covariance matrix $P(t_{k/k-1})$ from $t_{k-1}$ to $t_{k}$ with an initial predicted state error covariance matrix $P\left(t_{k-1}\right)=P_{0}=P_{init}\left(t=0\right)$ with uncorrelated elements as the following

(25) $$P_{init}\left(t=0\right)=\left[\begin{array}{ccccc} P_{x_{msv},x_{msv}}\left(t=0\right) & 0 & \cdots & 0 & 0\\ 0 & P_{x_{2},x_{2}}\left(t=0\right) & \cdots & 0 & 0\\ \vdots & \vdots & \ddots & \vdots & \vdots \\ 0 & 0 & \cdots & P_{n,n}\left(t=0\right) & 0\\ 0 & 0 & \cdots & 0 & P_{\theta_{up},\theta_{up}}\left(t=0\right) \end{array}\right],$$

we have

(26) $$P\left(t_{k/k-1}\right)=\left[\begin{array}{ccccc} P_{x_{msv},x_{msv}}\left(t_{k/k-1}\right) & P_{x_{2},x_{msv}}\left(t_{k/k-1}\right) & \cdots & P_{n,x_{msv}}\left(t_{k/k-1}\right) & P_{\theta_{up},x_{msv}}\left(t_{k/k-1}\right)\\ P_{x_{msv},x_{2}}\left(t_{k/k-1}\right) & P_{x_{2},x_{2}}\left(t_{k/k-1}\right) & \cdots & P_{n,x_{2}}\left(t_{k/k-1}\right) & P_{\theta_{up},x_{2}}\left(t_{k/k-1}\right)\\ \vdots & \vdots & \ddots & \vdots & \vdots \\ P_{x_{msv},n}\left(t_{k/k-1}\right) & P_{x_{2},n}\left(t_{k/k-1}\right) & \cdots & P_{n,n}\left(t_{k/k-1}\right) & P_{\theta_{up},n}\left(t_{k/k-1}\right)\\ P_{x_{msv},\theta_{up}}\left(t_{k/k-1}\right)=0 & P_{x_{2},\theta_{up}}\left(t_{k/k-1}\right) & \cdots & P_{n,\theta_{up}}\left(t_{k/k-1}\right) & P_{\theta_{up},\theta_{up}}\left(t_{k/k-1}\right) \end{array}\right].$$

Now, using $P(t_{k/k-1})$ , **H** and R to compute the Kalman gain for all variables in the state variable vector $\psi {\left(t\right)}_{general}$ (Equation (19)), we have

(27) $$K_{k}=P\left(t_{k|k-1}\right)H^{T}{\left(HP\left(t_{k|k-1}\right)H^{T}+R\right)}^{-1}=\left[\begin{array}{c} K_{x_{msv}}\\ K_{x_{2}}\\ \vdots \\ K_{x_{n}}\\ K_{\theta_{up}} \end{array}\right]=\left[\begin{array}{c} \frac{P_{x_{msv},x_{msv}}\left(t_{k/k-1}\right)}{P_{x_{msv},x_{msv}}\left(t_{k/k-1}\right)+R}\\ \frac{P_{x_{msv},x_{2}}\left(t_{k/k-1}\right)}{P_{x_{msv},x_{msv}}\left(t_{k/k-1}\right)+R}\\ \vdots \\ \frac{P_{x_{msv},n}\left(t_{k/k-1}\right)}{P_{x_{msv},x_{msv}}\left(t_{k/k-1}\right)+R}\\ \frac{P_{x_{msv},\theta_{up}}\left(t_{k/k-1}\right)}{P_{x_{msv},x_{msv}}\left(t_{k/k-1}\right)+R} \end{array}\right]=\left[\begin{array}{c} \frac{P_{x_{msv},x_{msv}}\left(t_{k/k-1}\right)}{P_{x_{msv},x_{msv}}\left(t_{k/k-1}\right)+R}\\ \frac{P_{x_{msv},x_{2}}\left(t_{k/k-1}\right)}{P_{x_{msv},x_{msv}}\left(t_{k/k-1}\right)+R}\\ \vdots \\ \frac{P_{x_{msv},n}\left(t_{k/k-1}\right)}{P_{x_{msv},x_{msv}}\left(t_{k/k-1}\right)+R}\\ \frac{0}{P_{x_{msv},x_{msv}}\left(t_{k/k-1}\right)+R} \end{array}\right]=\left[\begin{array}{c} \frac{P_{x_{msv},x_{msv}}\left(t_{k/k-1}\right)}{P_{x_{msv},x_{msv}}\left(t_{k/k-1}\right)+R}\\ \frac{P_{x_{msv},x_{2}}\left(t_{k/k-1}\right)}{P_{x_{msv},x_{msv}}\left(t_{k/k-1}\right)+R}\\ \vdots \\ \frac{P_{x_{msv},n}\left(t_{k/k-1}\right)}{P_{x_{msv},x_{msv}}\left(t_{k/k-1}\right)+R}\\ 0 \end{array}\right].$$

**H** selected the first column of $P(t_{k/k-1})$ , since it is related to the measured value $x_{msv}$ . However, in this column, we have that the predicted state error covariance between $x_{msv}$ and $\theta_{up}$ is zero, $P_{x_{msv},\theta_{up}}\left(t_{k/k-1}\right)=Cov\left(x_{msv},\theta_{up}\right)=0$ . The solution of $\frac{dP_{x_{msv},\theta_{up}}\left(t\right)}{dt}=0$ obtained from $t_{k-1}$ to $t_{k}$ is equal to the initial condition that is zero due to **P**(t = 0) with uncorrelated elements, and we have $Cov\left(x_{msv},\theta_{up}\right)=P_{x_{msv},\theta_{up}}\left(t_{k-1}\right)=P_{x_{msv},\theta_{up}}\left(t=0\right)=0$ . Then, the Kalman gain value for the unshared parameter is zero, $K_{\theta_{up}}$ = 0, and consequently, the predicted state error covariance $P_{x_{msv},\theta_{up}}\left(t_{k/k-1}\right)$ cannot be updated (by Equation (12)). Since

(28) $$\begin{array}{c} P\left(t_{k|k}\right)=\left(I-K_{k}H\right)P\left(t_{k|k-1}\right)=\left[\begin{array}{cc} \vdots & \\ P_{x_{msv},\theta_{up}}\left(t_{k/k-1}\right)-K_{\theta_{up}}.P_{x_{msv},x_{msv}}\left(t_{k/k-1}\right) & \ldots \end{array}\right]=\\ \left[\begin{array}{cc} \vdots & \\ 0-0.P_{x_{msv},x_{msv}}\left(t_{k/k-1}\right) & \ldots \end{array}\right]. \end{array}$$

Therefore, we have that $P_{x_{msv},\theta_{up}}\left(t_{k/k}\right)=P_{x_{msv},\theta_{up}}\left(t_{k/k-1}\right)=0$ , and as $P_{x_{msv},\theta_{up}}\left(t_{k/k}\right)=0$ has to be used as a new initial condition for MRDE (Equation (24)), we have $K_{\theta_{up}}$ = 0 for all $P_{x_{msv},\theta_{up}}\left(t_{k/k-1}\right)$ obtained from $t_{k-1}$ to $t_{k}$ using Equation (24) and consequently $K_{\theta_{up}}$ and $P_{x_{msv},\theta_{up}}\left(t_{k/k}\right)=P_{x_{msv},\theta_{up}}\left(t_{k/k-1}\right)=0$ are always zero and cannot be updated. □

#### 4.1.3. Theorem: JEKF Failure

The consequence of Lemma 1 (Section 4.1.2) is the following theorem:

**Theorem** **1.** *The JEKF (Section 3.3) fails to estimate an unshared parameter (parameter evolution) that is part of a state variable vector and part of a weak term in a UMM if the initial state error covariance matrix* ***P**(t = 0) and* ***Q*** *are composed of uncorrelated elements, and there is only one state variable measured. This is because the Kalman gain value for the unshared parameter is equal to zero for all steps of execution of the JEKF algorithm.*

The proof of Theorem 1 is in the following, and an example of this theorem can be found in Section S5 of the Supplementary Material.

**Proof of Theorem** **1.** This proof can be completed using the conditions and results described previously in the proof of Lemma 1 (Section 4.1.2).

Then, let us consider the following:

- **H** = [1 0 … 0 0] and $K_{k}={\left[K_{x_{msv}},K_{x_{2}},\cdots ,K_{x_{n}},K_{\theta_{up}}\right]}^{T}$ as obtained in the proof of Lemma 1 in Section 4.1.2, where $K_{\theta_{up}}$ = 0.
-
$Z_{k}$ as a measured value of $x_{msv}$ .
- Predicted mean of the state variable vector $\hat{\psi }{\left(t_{k/k-1}\right)}_{general}={\left[{\hat{x}}_{msv},{\hat{x}}_{2},\cdots ,{\hat{x}}_{n},{\hat{\theta }}_{up}\right]}^{T}$ with regard to the general UMM used in the proof of Lemma 1 in Section 4.1.2.

Now, using Equation (11) to compute the estimated mean of the state variable vector $\hat{\psi }{\left(t_{k/k}\right)}_{general}$ , we have

(29) $$\hat{\psi }{\left(t_{k/k}\right)}_{general}=\hat{\psi }{\left(t_{k/k-1}\right)}_{general}+K_{k}\left(Z_{k}-H\hat{\psi }{\left(t_{k/k-1}\right)}_{general}\right)$$

(30) $$\hat{\psi }{\left(t_{k/k}\right)}_{general}=\left[\begin{array}{c} {\hat{x}}_{msv}\\ {\hat{x}}_{2}\\ \vdots \\ {\hat{x}}_{n}\\ {\hat{\theta }}_{up} \end{array}\right]+\left[\begin{array}{c} K_{x_{msv}}\\ K_{x_{2}}\\ \vdots \\ K_{x_{n}}\\ K_{\theta_{up}} \end{array}\right].\left(Z_{k}-{\hat{x}}_{msv}\right)=\left[\begin{array}{c} {\hat{x}}_{msv}+K_{x_{msv}}.\left(Z_{k}-{\hat{x}}_{msv}\right)\\ {\hat{x}}_{2}+K_{x_{2}}.\left(Z_{k}-{\hat{x}}_{msv}\right)\\ \vdots \\ {\hat{x}}_{n}+K_{x_{n}}.\left(Z_{k}-{\hat{x}}_{msv}\right)\\ {\hat{\theta }}_{up}+0 \end{array}\right]$$

Then, we have that the estimated mean of the unshared parameter ${\hat{\theta }}_{up}\left(t_{k/k}\right)$ (composing the $\hat{\psi }{\left(t_{k/k}\right)}_{general}$ ) is equal to the predicted mean of unshared parameter ${\hat{\theta }}_{up}\left(t_{k/k-1}\right)$ (composing the $\hat{\psi }{\left(t_{k/k-1}\right)}_{general}$ ) for all steps from $t_{k-1}$ to $t_{k}$ . In other words, the JEKF fails to perform the parameter evolution, since it does not have a noise component to evolve the parameter as described in the $\theta \left(t_{k}\right)=\theta \left(t_{k-1}\right)+noise$ (Equation (14)); then, ${\hat{\theta }}_{up}\left(t_{k/k}\right)={\hat{\theta }}_{up}\left(t_{k/k-1}\right)$ for all steps from $t_{k-1}$ to $t_{k}$ . □

### 4.2. SANTO: Specific Initial Condition for MRDE ( $P_{MSV,UP}\left(t=0\right)\neq 0\;in$ $P_{0}$ )

This section presents the SANTO approach to avoid the JEKF failure described in Theorem 1. The initial condition of MRDE is the initial state error covariance matrix $P_{0}=P\left(t=0\right)$ . When it is composed of uncorrelated elements (P ${\text{​}}_{i,j}=0$ ), some initial conditions of time-invariant ODEs ( $\frac{dP_{i,j}\left(t_{k|k-1}\right)}{dt}=0$ ) in the MRDE are zero, and consequently, the obtained solutions from $t_{k-1}$ to $t_{k}$ for some of these time-invariant ODEs are zero, too. Furthermore, in the presence of the biomanufacturing conditions (failure case presented in Section 4.1.1), we have that the Kalman gain value regarding the unshared parameter ( $K_{UP}$ ) and the predicted state error covariance between the unique measured state variable and the unshared parameter ( $P_{MSV,UP}\left(t_{k|k-1}\right)$ ), are zero too, $K_{UP}=0$ and $P_{MSV,UP}\left(t_{k|k-1}\right)=0$ . Then, the $K_{UP}$ and $P_{MSV,UP}\left(t_{k|k-1}\right)$ that compose $P(t_{k|k-1})$ cannot be updated with regard to the unshared parameter (see Lemma 1), and they are constant and equal to zero during the entire process execution of JEKF. It is worth noting that $P_{MSV,UP}\left(t_{k|k-1}\right)$ is an element of $P(t_{k|k-1})$ such as $P_{MSV,UP}\left(t=0\right)$ is an element of P(t=0) . Furthermore, that $K_{UP}=0$ during the entire JEKF execution reflects an unrealistic situation. This would mean that the prediction regarding the unshared parameter is perfect and does not need the influence of the measurement in the correction step of JEKF since there is no uncertainty in the prediction regarding the unshared parameter. This reflects the second intuition behind Kalman gain described in Section S2 of the Supplementary Material. However, based on prior knowledge, we know that the process model predictions regarding the unshared parameter are imperfect since we need to perform the evolution of the unshared parameter; otherwise, they would be the same during the entire process. Therefore, we need $K_{UP}\neq 0$ and $P_{MSV,UP}\left(t_{k|k-1}\right)\neq 0$ .

In general, the initial condition of MRDE is **P** (t=0) with uncorrelated elements (P ${\text{​}}_{i,j}=0$ ) due to the difficulty of estimating all covariances with a limited dataset. However, instead, considering all off-diagonal elements of P(t=0) equal zero (P ${\text{​}}_{i,j}=0$ ), we can consider only the key off-diagonal element (that is P ${\text{​}}_{MSV,UP}\left(t=0\right)$ ) with an initial value different of zero ( $P_{MSV,UP}\left(t=0\right)\neq 0$ ) to avoid the failure case. This value could be a positive quantity, λ , since the off-diagonal elements of P(t=0) can show a positive covariance between two variables, indicating that they tend to increase or decrease together. Furthermore, the value of λ should be different from zero and small enough to not significantly affect the filter’s estimates but large enough to prevent the failure case. Then, with this consideration, we can have a value for the initial state error covariance between the MSV and an UP (P ${\text{​}}_{MSV,UP}\left(t=0\right)$ ). If we add it to the initial state error covariance matrix P(t=0) with the other uncorrelated elements, we have a specific initial condition for MRDE that enables us to update the $K_{UP}$ and $P_{MSV,UP}\left(t_{k|k-1}\right)$ present in $P(t_{k|k-1})$ and, consequently, avoids the JEKF failure.

**Theorem** **2** (SANTO—Proposed approach to avoid the JEKF failure)**.** *The addition of a positive quantity (λ) to the $P_{MSV,UP}\left(t=0\right)$ in P(t=0) to initialize the MRDE with a specific initial condition can prevent the Kalman gain being zero in the entire execution of JEKF and prevent the JEKF failure (Section 4.1).*

**Proof.**  The proof of the SANTO approach can be completed using the conditions described previously in the proof of Lemma 1 (Section 4.1.2) and Theorem 1 (Section 4.1.3).

Then, let us consider the following:

- A **positive** quantity λ .
-
$x_{msv}$ as the unique measured state variable (MSV) and H = [1 0 … 0 0].
- R as measurement noise variance of $x_{msv}$ .
-
$\theta_{up}$ as the unshared parameter (UP) to be evolved (estimated) and presented in only one weak term.
- A specific initial predicted state error covariance matrix $P\left(t_{k-1}\right)=P_{0}=P_{santo}\left(t=0\right)$ with uncorrelated elements and $P_{MSV,UP}\left(t=0\right)=P_{x_{msv},\theta_{up}}\left(t=0\right)=\lambda$ as following
(31) $$P_{santo}\left(t=0\right)=\left[\begin{array}{ccccc} P_{x_{msv},x_{msv}}\left(t=0\right) & 0 & \cdots & 0 & 0\\ 0 & P_{x_{2},x_{2}}\left(t=0\right) & \cdots & 0 & 0\\ \vdots & \vdots & \ddots & \vdots & \vdots \\ 0 & 0 & \cdots & P_{n,n}\left(t=0\right) & 0\\ P_{x_{msv},\theta_{up}}\left(t=0\right)=\lambda & 0 & \cdots & 0 & P_{\theta_{up},\theta_{up}}\left(t=0\right) \end{array}\right],$$

Now, using this Equation (24) to compute the predicted state error covariance matrix $P(t_{k/k-1})$ from $t_{k-1}$ to $t_{k}$ with the specific initial predicted state error covariance matrix $P\left(t_{k-1}\right)=P_{0}=P_{santo}\left(t=0\right)$ , we have

(32) $$P\left(t_{k/k-1}\right)=\left[\begin{array}{ccc} \vdots & \vdots & \\ P_{x_{msv},\theta_{up}}\left(t_{k/k-1}\right)=\lambda & P_{x_{2},\theta_{up}}\left(t_{k/k-1}\right) & \cdots \end{array}\right].$$

where $P_{x_{msv},\theta_{up}}\left(t_{k/k-1}\right)=\lambda$ because the solution of $\frac{dP_{x_{msv},\theta_{up}}\left(t\right)}{dt}=0$ obtained from $t_{k-1}$ to $t_{k}$ is equal to the initial condition that is λ in $P_{0}$ . Now, using $P(t_{k/k-1})$ , **H** and R to compute the Kalman gain for all variables in the state variable vector ψ(t) (Equation (19)), we have

(33) $$K_{k}=P\left(t_{k|k-1}\right)H^{T}{\left(HP\left(t_{k|k-1}\right)H^{T}+R\right)}^{-1}=\left[\begin{array}{c} \vdots \\ K_{\theta_{up}} \end{array}\right]=\left[\begin{array}{c} \vdots \\ \frac{P_{x_{msv},\theta_{up}}\left(t_{k/k-1}\right)}{P_{x_{msv},x_{msv}}\left(t_{k/k-1}\right)+R} \end{array}\right]=\left[\begin{array}{c} \vdots \\ \frac{\lambda }{P_{x_{msv},x_{msv}}\left(t_{k/k-1}\right)+R} \end{array}\right].$$

Then, we have the Kalman gain value for the unshared parameter as

(34) $$K_{\theta_{up}}=\lambda {\left(P_{x_{msv},x_{msv}}\left(t_{k/k-1}\right)+R\right)}^{-}\neq 0,$$

and consequently, the predicted state error covariance $P_{x_{msv},\theta_{up}}\left(t_{k/k-1}\right)$ can be updated by Equation (12) and predicted mean of the state variable vector with regard to UP, ${\hat{\theta }}_{up}\left(t_{k/k-1}\right)$ can be updated as Equation (11). Therefore, we have $P_{x_{msv},\theta_{up}}\left(t_{k/k}\right)\neq P_{x_{msv},\theta_{up}}\left(t_{k/k-1}\right)$ and ${\hat{\theta }}_{up}\left(t_{k/k}\right)\neq {\hat{\theta }}_{up}\left(t_{k/k-1}\right)$ during the entire execution of JEKF. □

It is essential to point out that the SANTO is inspired by the idea of a regularization technique used to avoid the singularity problem in the state error covariance matrix [35,56]. However, instead of adding a small quantity to the diagonal elements of the state error covariance matrix **P**, such as the perturbed-P algorithm [35], we only add a positive quantity ( λ ) to the $P_{MSV,UP}\left(t=0\right)$ in P(t=0) to initialize the MRDE. Furthermore, a positive quantity to the P ${\text{​}}_{MSV,UP}\left(t=0\right)$ can be defined by empirical tuning. One of the most common ways to define a quantity is by trial and error. This involves running the filter with different values of λ and choosing the value that results in the best performance [57].

**Figure 1:**
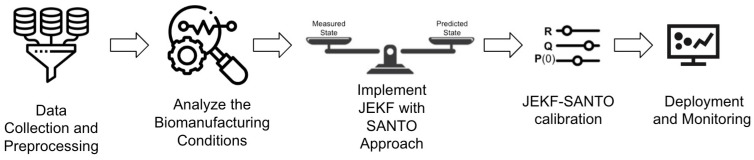
The basic steps to develop a soft sensor for bioprocess monitoring based on JEKF-SANTO.

Figure 1 shows the steps to develop a soft sensor for bioprocess monitoring based on JEKF-SANTO.

- Step 1: Data Collection and Preprocessing. The first step in developing a soft sensor for bioprocess monitoring using the JEKF-SANTO approach involves comprehensive data collection and preprocessing. Once collected, these data must be meticulously cleaned and preprocessed to remove outliers and address any missing values. This preprocessing is crucial to ensure the quality and reliability of the data, which forms the foundation for accurate modeling and estimation in subsequent steps.
- Step 2: Analyze the Biomanufacturing Conditions. This step involves a comprehensive analysis of the biomanufacturing conditions where JEKF fails to estimate an unshared parameter that is part of a state variable vector and part of a weak term in a UMM if the initial state error covariance matrix **P**(t = 0) and **Q** are composed of uncorrelated elements, and there is only one state variable measured.
- Step 3: Implement JEKF with the SANTO approach. Implement the JEKF algorithm, defining the process model and the measurement model. Modify the initial state error covariance matrix P(t=0) as per the SANTO approach, adding a specific positive quantity λ to the covariance between the measured state variable and the unshared parameter.
- Step 4: JEKF-SANTO calibration. Tune the R and Q of JEKF-SANTO based on consistency tests, and adjust the λ parameter model based on the estimates obtained from JEKF-SANTO related to the unshared parameter and the associated weak variable.
- Step 5: Deployment and Monitoring. Integrate the JEKF-SANTO as a soft sensor into the biomanufacturing process control system to monitor critical quality attributes (CQAs) and critical process parameters (CPPs) in real time.

## 5. Empirical Evaluation

In our evaluation, we have the two goals (**G1** and **G2**) that are addressed by answering three Research Questions (**RQs**) comparing three NSEs: JEKF-Classic, JEKF-SANTO and JEKF-KPH2. First, the goals are the following:

- (**G1**) Experimentally test Theorem 1 (JEKF Failure ) in Section 4.1.3;
- (**G2**) Test whether SANTO can avoid the JEKF failure and compare its performance with KPH2.

Lastly, the research questions are the following:

- (**RQ1-G1**) Is there any variation in the unshared parameter estimation completed by JEKF-Classic with the biomanufacturing conditions (failure case), or are the estimations constant in the entire process?
- (**RQ2-G2**) Is there any variation in the unshared parameter estimation completed by SANTO and KPH2 with the biomanufacturing conditions (failure case), and which one has the best estimations (performance)?
- (**RQ3-G2**) Can the SANTO simultaneously estimate more than one unshared parameter, performing better than KPH2?

### 5.1. Experimental Setup

#### 5.1.1. Synthetic Dataset—mAb Production

**Figure 2:**
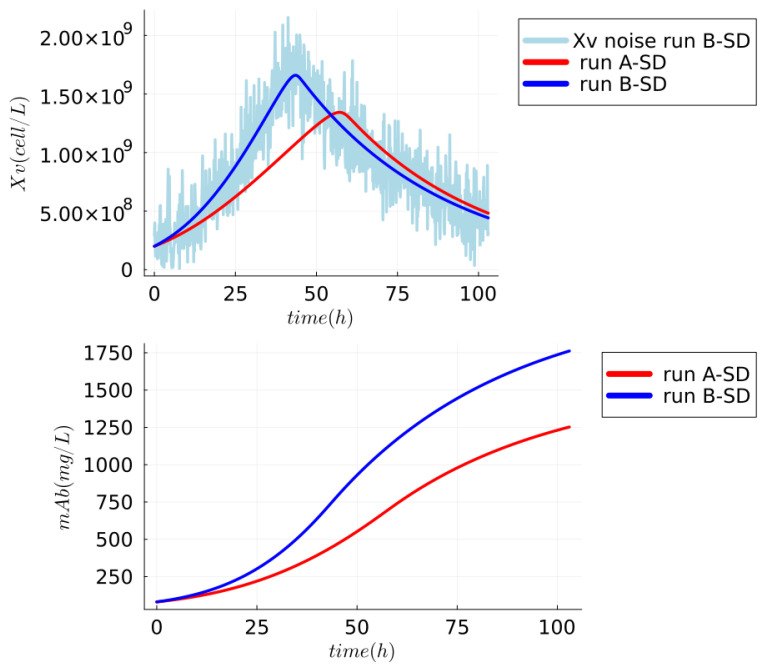
Synthetic dataset regarding mAb production. The run A-SD (red lines) was generated using the original parameters proposed by [59]. Run B-SD (blue lines) has the maximum cell expansions and the maximum mAb (titer) production of SD. The $X_{v}$ of B-SD with noise is highlighted in light blue in the first plot. This noise is used to evaluate the performance of the NSE to estimate mAb and QmAb.

The synthetic dataset (SD) has data regarding Monoclonal Antibody (mAb) productions that represent the biomanufacturing of a protein widely used as diagnostic reagents and for therapeutic purposes [58]. The SD comprises two runs (A-SD and B-SD) with different cell expansions and maximums of the mAb (titer) production. The runs of SD can be seen in Figure 2, and the runs have a sample rate of 7.5 minutes during 103 hours of the process. The runs were generated using the UMM proposed by [59] with small variations in parameters $\mu_{max}$ (maximum growth rate) and QmAb (mAb specific production rate) (see Table S1 of the Supplementary Material) but with the same initial concentrations of states variables (viable cell density (Xv), glucose (GLC), glutamine (GLN), lactate (LAC), ammonium (AMM) and mAb) and with different conditions of pH and temperature as completed in the synthetic dataset of [55]. The run A-SD (red lines in plots of Figure 2) was generated using the original parameters proposed by [59], which are the parameters $\mu_{max}$ = $5.8\times 10^{-9}($ h ${\text{​}}^{-})$ and QmAb = 7.21 ( $\times 10^{-9}\;$ mg cells ${\text{​}}^{-1}$ h ${\text{​}}^{-1}$ ). Run B-SD (blue lines in plots of Figure 2) has the maximum cell expansions and a maximum of mAb (titer) production of SD, and they were obtained with the parameters $\mu_{max}$ = $7.5\times 10^{-9}($ h ${\text{​}}^{-})$ and QmAb = 9.21 ( $\times 10^{-9}\;$ mg cells ${\text{​}}^{-1}$ h ${\text{​}}^{-1}$ ). Furthermore, the run B-SD has samples regarding X ${\text{​}}_{V}$ (cell/L) with Gaussian white noise, and they were created by adding the Gaussian white noise with a standard deviation of 20 $\times \;10^{7}$ to the data represented in blue and green lines. The $X_{v}$ of B-SD with noise is highlighted in light blue in the first plot. It is essential to point out that X ${\text{​}}_{V}$ samples with Gaussian white noise represent a possible online measurement with a sensor that includes noises. This noise is used to evaluate the performance of the NSEs (JEKF-Classic, JEKF-SANTO, and JEKF-KPH2) to estimate mAb and QmAb.

#### 5.1.2. Real Dataset: AAV Production

The real dataset (RD) contains data regarding rAAV productions, which are described and available in [19]. rAAV is a viral vector technology for gene therapy that is considered the safest and most effective way to repair single-gene abnormalities in non-dividing cells [60]. The RD has two runs with online and offline measurements of the state variables viable cell density (Xv), glucose (GLC), glutamine (GLN), lactate (LAC), ammonium (AMM), and rAAV (titer) regarding the rAAV production in shake-flasks and in bioreactors. The run A-RD (production in shake-flasks) has only offline measurements, and the run B-RD (production in bioreactor) has online measurements of Xv and offline measurements of GLC, LAC, and rAAV (titer). The samples of the runs add up to 2902 with a sample rate of 1 minute during 48.3 hours of the process. The details of the real dataset development can be seen in [19].

#### 5.1.3. NSEs Assessment with Synthetic Dataset to Address RQ1-G1 and RQ2-G2

All NSEs (JEKF-Classic, JEKF-SANTO, and JEKF-KPH2) used the UMM described in Section S1.4 of the Supplementary Material as a process model and the same initial concentration regarding the state variables; see Table S2 of the Supplementary Material. The NSEs were used to correct (estimate) the predictions regarding state variables (Xv and mAb) and to evolve the unshared parameter (QmAb) of the process model. This was accomplished using the Xv samples with the noise of the run B-SD as the unique measured state variable and the parameters used to generate the run A-SD as initial parameters of the process model (see Tables S1 and S2 of Supplementary Material). This situation represents a joint estimation problem where the prediction and parameter of the process model should be corrected by the NSEs based on measured state variable Xv with noise. For example, the initial value used for QmAb is the value of run A-SD (QmAb = 7.21 $\times \;10^{-9}\;$ mg cells ${\text{​}}^{-1}$ h ${\text{​}}^{-1}$ ), and it should be evolved to the value of run B-SD (9.21 $\times \;10^{-9}$ mg cells ${\text{​}}^{-1}$ h ${\text{​}}^{-1}$ ) based on Xv with the noise of run B-SD. Furthermore, the Xv (without noise) and mAb samples of run B-SD were used as ground truth, too. It is important to point out that the estimations were made with MRDE formed by **P** with correlated elements (MRDE-PC) and uncorrelated elements (MRDE-PU). In addition, MRDE-PC and MRDE-PU were combined with standard and specific **P**(t = 0) to check the sensitivity of SANTO (with regard to P ${\text{​}}_{MSV,UP}\left(t=0\right)$ ) and KPH2 (with regard to P ${\text{​}}_{UP,UP}\left(t=0\right)$ ). The standard **P**(t = 0) means that all NSEs used the same **P**(t = 0). On the other hand, the specific **P**(t = 0) means that each NSE used a different **P**(t = 0) that enables its best performance. For example, the specific **P**(t = 0) for SANTO contains a specific value of P ${\text{​}}_{MSV,UP}\left(t=0\right)$ , and the specific **P**(t = 0) for KPH2 includes specific value of P ${\text{​}}_{UP,UP}\left(t=0\right)$ . The specific **P**(t = 0) was obtained by trial and error, and a standard **Q** was used for all NSEs. For example, the specific **P**(t = 0) for SANTO contains a specific value of P ${\text{​}}_{MSV,UP}\left(t=0\right)=\lambda$ , and the specific **P**(t = 0) for KPH2 includes a specific value of P ${\text{​}}_{UP,UP}\left(t=0\right)$ . The values of specific **P**(t = 0) (including λ ) were obtained by trial and error. Furthermore, a standard and specific **Q** were also used for all NSEs. In addition, the root-mean-square percentage error (RMSPE) was used as a metric to assess the similarity between NSEs estimations and the ground truth of run B-SD. The details about the design of NSEs with SD can be found in the Section S7.3 of the Supplementary Material.

#### 5.1.4. NSEs Assessment with Real Dataset to Address RQ3-G2

The NSEs (JEKF-Classic, JEKF-SANTO, and JEKF-KPH2) used the UMM described in Section S1.5 of the Supplementary Material as a process model and the same initial concentration regarding the state variables; see Table S8 of the Supplementary Material. These three NSEs were used to correct (estimate) the predictions regarding Xv, GLC, LAC, and rAAV (titer) and to evolve the unshared parameters ( $\mu_{Lac}$ , $\mu_{GLC}$ and $\mu_{rAAV}$ ) of the process model. This was accomplished using the Xv samples with the noise of the run B-RD as the unique measured state variable and the parameters obtained with the run A-SD as initial parameters (see Table S9 of the Supplementary Material). This situation also represents a joint estimation problem where the predictions and parameters of the process model should be corrected simultaneously by the NSEs based on measured state variable Xv with noise. However, in this case, the NSEs have to correct three different unshared parameters simultaneously based on Xv with the noise of run B-RD. Furthermore, the RMSPE was used as a metric to assess the similarity between NSEs estimations and the ground truth of run B-SD, which are the offline measurements of GLC, LAC and rAAV (titer) of run B-RD. It is essential to point out that the estimations were also completed with MRDE-PC and with specific **P**(t = 0). The details about the design of NSEs with RD can be found in Section S7.4 of the Supplementary Material.

#### 5.1.5. Checking Consistency and Efficiency

The calibration of standard and specific **Q** were based on consistency tests, specifically the innovation magnitude bound (IMB) test and the normalized innovations squared (NIS) Chi-square test [61]. These two tests are used to check that the NSEs are performing correctly with **Q** and **R** selected [50,62].

**Figure 3:**
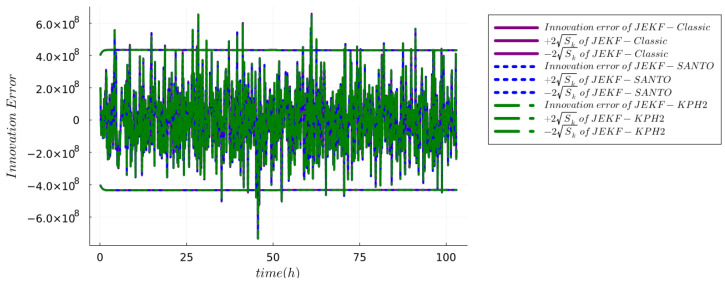
Innovation magnitude bound test using the run B of synthetic dataset for the NSEs with MRDE-PC and specific **Q** and **P**(0).

**Figure 4:**
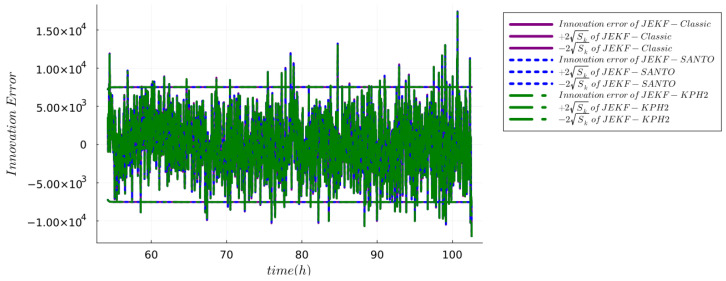
Innovation magnitude bound test using the run B of real dataset for the NSEs with MRDE-PC and specific **Q** and **P**(0).

**IMB Test.** It checks that the innovation is consistent with its covariance by verifying that the magnitude of the innovation is bounded by $\pm 2\sqrt{S_{k}}$ . A positive result in this test occurs when at least 95% of the values of the innovation lie within the $\pm 2\sqrt{S_{k}}$ . Figure 3 presents the innovation error sequence for the NSEs configured with MRDE-PC, utilizing specific **Q** and **P**(0) settings as detailed in Tables S4–S6 of the Supplementary Material using run B of the synthetic dataset. This figure demonstrates that the innovation errors are unbiased with approximately 95.14% of the values falling within the $\pm 2\sqrt{S_{k}}$ bounds as required. Similar outcomes were observed for NSEs configured with both MRDE-PC and MRDE-PU, and irrespective of whether standard or specific **Q** and **P**(0) settings were employed, as shown in Figures S1–S3 using **Q** and **P**(0) as detailed in Tables S4, S5 and S7 of the Supplementary Material. Each of these configurations yielded similar innovation error characteristics, underscoring the robustness of the NSEs under varying conditions. Furthermore, similar results were also obtained with run B of the real dataset. Figure 4 presents the innovation error sequence for the NSEs configured with MRDE-PC, utilizing specific **Q** and **P**(0) settings as detailed in Tables S10 and S11 of the Supplementary Material. This figure demonstrates that the innovation errors are unbiased with approximately 95.9% of the values falling within the $\pm 2\sqrt{S_{k}}$ bounds as required.

**Figure 5:**
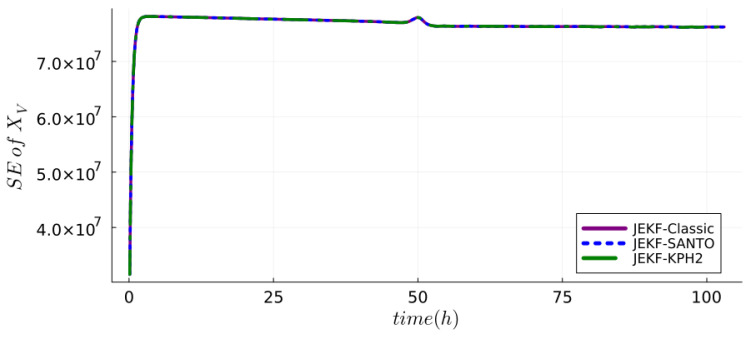
Standard error of $X_{V}$ at each k estimated by NSEs with synthetic dataset (run B) using MRDE-PC and specific P(0).

**Figure 6:**
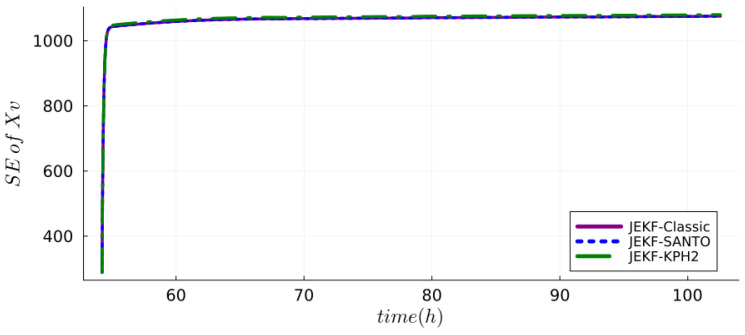
Standard error of $X_{V}$ at each k estimated by NSEs with real dataset using MRDE-PC and specific P(0).

Complementarily, standard error (SE) plots, based on the **P** matrix’s diagonal, demonstrate the changing uncertainty in state estimates. Filter stability and consistency are indicated by SEs, related to the measured state variable, converging to a stable value. This convergence signifies the adaptability and equilibrium of a filter in making accurate predictions. The alignment of positive innovation test results with this convergent SE (of measured state variable) trend substantiates the overall stability and consistency of a filter. Figure 5 depicts the SE over time of $X_{V}$ (measured state variable) estimated by NSEs with a synthetic dataset using MRDE-PC and specific **P**(0). Initially, these errors exhibited an increase, reflecting a period of adaptation as the filter assimilated the initial data. However, after this initial phase, the standard errors converged around a stable value. This convergence signifies the increasing reliability of the filter in estimating the state of $X_{V}$ as it processed more data. The initial increase followed by a steady convergence of the standard errors, in tandem with the favorable innovation test results, compellingly demonstrates the robustness of the NSEs. Similar results were obtained with NSEs with the synthetic dataset using MRDE-PU and specific **P**(0) (Figure S4 of the Supplementary Material) and with NSEs with the real dataset using MRDE-PC and specific **P**(0); see Figure 6. It is important to point out that Figures S5–S8 in the Supplementary Material show the normal behavior of standard errors for the state variables (QmAb and mAb) estimated by JEKF-SANTO and JEKF-KPH2 with the synthetic dataset. Similarly, Figures S10–S14 in the Supplementary Material show the standard errors for the state variables (GLC, LAC, rAAV, $\mu_{\mathrm{GLC}}$ , $\mu_{\mathrm{LAC}}$ , and $\mu_{\mathrm{rAAV}}$ ) estimated by JEKF-SANTO and JEKF-KPH2 with the synthetic dataset.

**NIS Chi-square Test.** It verifies that the innovation is unbiased and white by using hypothesis testing ( $\chi^{2}$ test) [50,62]. The NIS is defined as $NIS_{k}=e_{Z,k}S_{k}^{-}e_{Z,k}$ , and the mean of NIS is defined as $\mu \left(NIS\right)=\frac{1}{N}\sum_{k=1}^{N}e_{Z,k}S_{k}^{-}e_{Z,k}$ from a single run of a JEKF. Therefore, the NIS test involves verifying that μ(NIS) lies in the confidence interval [r1, r2] defined by the hypothesis $H_{0}$ that N×μ(NIS) is $\chi_{Nm}^{2}$ distributed with probability 1 − α , such that $P(N\times \mu \left(NIS\right)\in \left[r1,r2\right]|H_{0})=1-\alpha$ where *m* is the number of measured state variables and *N* is the number of samples from the measured state variables. In our case, *m* = 1 because we have only one measure state variable, and N = 824 for SD and N = 2901 for RD. Furthermore, for the case of a two-sided 95% confidence region, we have $\left[r1,r2\right]=[\chi_{Nm}^{2}\left(0.025\right),\chi_{Nm}^{2}\left(0.975\right)]$ . Therefore, the NIS test of NSEs with the synthetic dataset is concerned with answering the following question: Is N×μ(NIS) inside of $[\chi_{824}^{2}\left(0.025\right),\chi_{824}^{2}\left(0.975\right)]=\left[745.39,904.39\right]$ where N = 824, such that $P(N\times \mu \left(NIS\right)\in \left[745.39,904.39\right]|H_{0})=1-\alpha$ ? All the NSEs designed with the synthetic dataset using the **Q**, **R** and **P**(0) defined in Tables S3–S7 of the Supplementary Material presented the N×μ(NIS) falling inside of the confidence bound defined by the χ ^2^ test. The NSEs with MRDE-PU had an N×μ(NIS)=835.29 and NSEs with MRDE-PC presented a N×μ(NIS)=830.35 . Furthermore, the NSEs with the real dataset had a positive result in the $\chi^{2}$ test with the following question: Is N×μ(NIS) inside of $[\chi_{2901}^{2}\left(0.025\right),\chi_{2901}^{2}\left(0.975\right)]=\left[2752.63,3051.15\right]$ where N = 2901? NSEs with MRDE-PC and, **Q**, **R** and **P**(0) defined in Tables S10 and S11 of the Supplementary Material presented an N×μ(NIS)=2840.55 .

**Figure 7:**
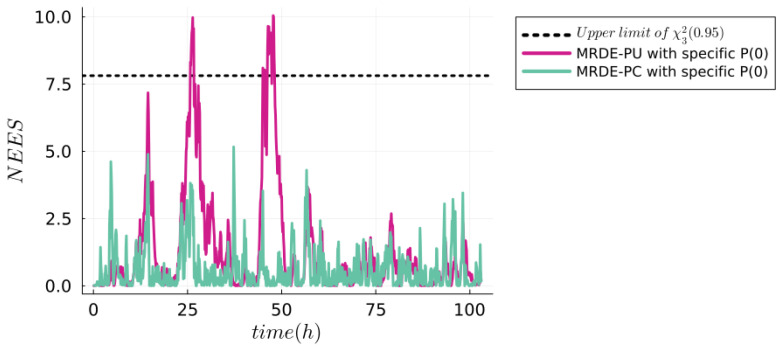
NEES test of JEKF-SANTO with synthetic dataset. In case of JEKF-SANTO with MRDE-PC with specific P(0), we have that 100% of all NEES computed are found inside the one-sided 95% probability region where the 5% tail is $\chi_{3}^{2}\left(0.95\right)=7.815$ (upper limit). In case of JEKF-SANTO with MRDE-PU with specific P(0), we have 98.4% of the NEES inside of confident interval [0, $\chi_{3}^{2}\left(0.95\right)=7.815$ ].

**Normalized estimation error squared (NEES) test**. It is the metric used to evaluate the efficiency of the JEKF-SANTO as an estimator. This involves verifying that the actual estimation errors ( $e_{x,k}$ ) appropriately match the predictions made by the **P**(t ${\text{​}}_{k/k}$ ) [62]. Essentially, if the **P**(t ${\text{​}}_{k/k}$ ) predicts a certain degree of uncertainty, it is expected for the real-world errors $e_{x,k}$ to match this prediction. This match is crucial for the estimator to be considered accurate and reliable. NEES is calculated as $\mathrm{NEES}\left(k\right)=e_{x,k}^{⊤}P{\left(t_{k/k}\right)}^{-1}e_{x,k}$ where $e_{x,k}$ is the estimation error at time step *k*, defined as $e_{x,k}=x\left(t_{k}\right)-\hat{x}\left(t_{k/k}\right)$ , with $x(t_{k})$ being the true state and $\hat{x}\left(t_{k/k}\right)$ being the estimated state. Then, for the case of a single run, the NEES(k) is Chi-square distributed with n ${\text{​}}_{x}$ degrees of freedom. In our case, we have n ${\text{​}}_{x}=3$ because we are concerned with evaluating the performance of JEKF-SANTO to estimate the states $X_{V}$ , QmAb and mAb of the synthetic dataset. Therefore, we consider a one-sided 95% probability region as seen in Bar-Shalom et al. in [62] for single-run simulation tests with small degrees of freedom. We have the hypothesis H ${\text{​}}_{0}$ that JEKF-SANTO’s efficiency ( $e_{x,k}$ matches **P**(t ${\text{​}}_{k/k}$ )), and H ${\text{​}}_{0}$ is accepted if $P(NEES\left(k\right)\leq \chi_{3}^{2}\left(0.95\right)=7.815|H_{0})=1-\alpha$ . Figure 7 depicts the result of NEES for the JEKF-SANTO with the synthetic dataset using MRDE-PC and MRDE-PU. The designated upper threshold for the acceptance region is set at 7.815. The majority of the NEES(k) values are observed to fall within the defined confidence interval [0, $\;\chi_{3}^{2}\left(0.95\right)=7.815$ ], which means the estimation error and the covariance are compatible with each other, and the estimation of the JEKF-SANTO is reliable and credible. Moreover, these findings are in alignment with those reported by Bar-Shalom et al. in [62], particularly in the context of single-run simulation tests with a small number of degrees of freedom.

## 6. Results

The results are organized by research questions **RQ1-G1**, **RQ2-G2** and **RQ3-G2**.

**Figure 8:**
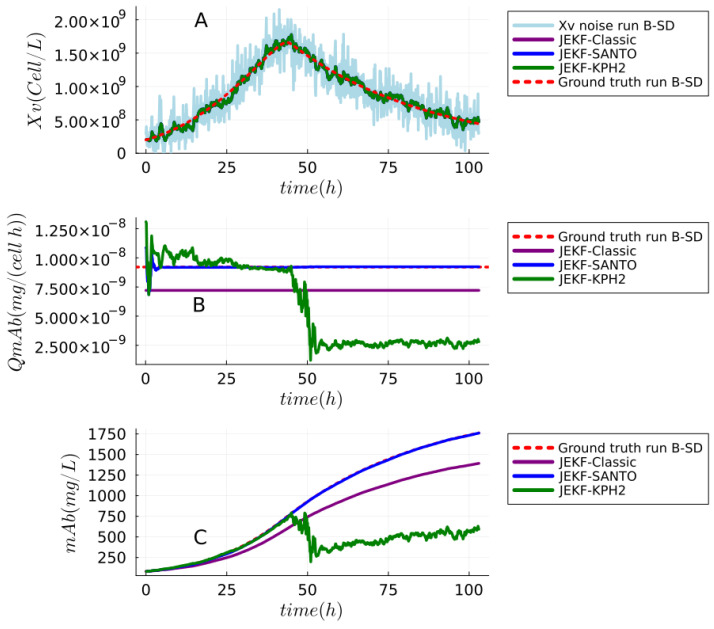
Experimental test of the theorem (JEKF failure) and the JEKF-SANTO to avoid the JEKF failure with the biomanufacturing conditions (failure case). This experiment used run B of the synthetic dataset, and plot (**A**) shows that all estimations with regard to Xv were close to the ground truth. Plots (**B**,**C**) show the estimations with regard to the unshared parameter QmAb and mAb (titer), respectively. The JEKF-SANTO was able to evolve QmAb with convergence to the ground truth value, but JEKF-KPH2 and JEKF-Classic failed. They were not able to evolve the mAb. All NSEs were executed with MRDE-PC and specific **P**(t = 0).

**Figure 9:**
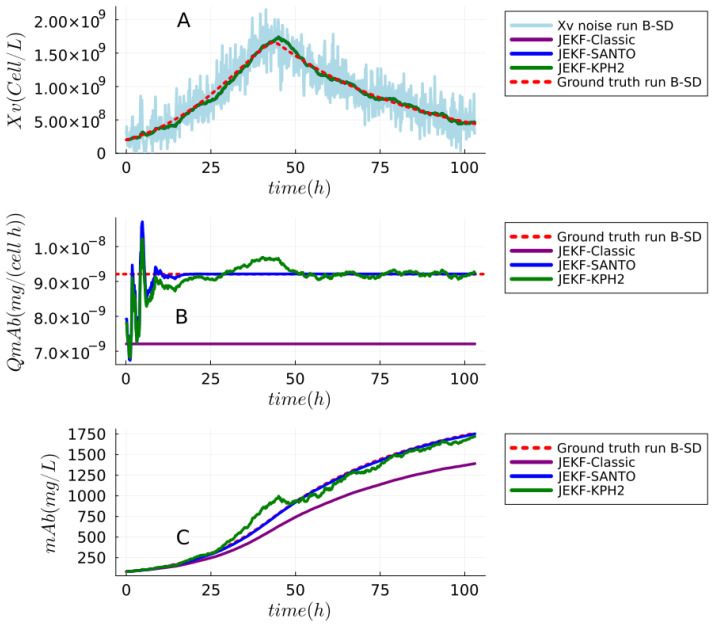
Experimental test that JEKF-Classic cannot avoid the JEKF failure with run B of the synthetic dataset. First, plot (**A**) shows the estimations regarding Xv, and all estimations were close to the ground truth. The plots (**B**,**C**) show the estimations regarding the unshared parameter QmAb and mAb (titer), respectively. All NSEs evolved QmAb with convergence to the ground truth value except JEKF-Classic. All NSEs were executed with MRDE-PU and specific P ${\text{​}}_{UP,UP}\left(t=0\right)$ .

**Figure 10:**
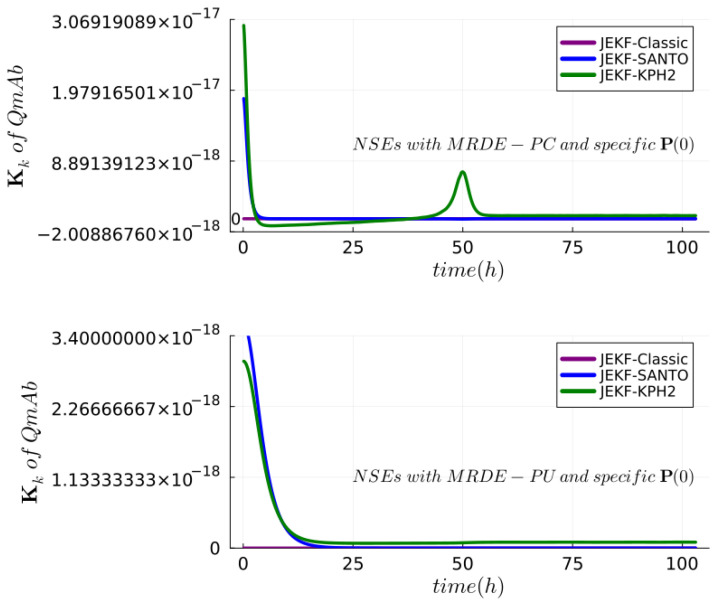
Kalmain gain over time for the NSEs with run B of synthetic dataset. In all cases, JEKF-Classic is constant and equal to zero.

**Answer to RQ1-G1.**The results of the experimental test of Theorem 1 (JEKF failure) can be seen in Figure 8 and Figure 9. We also reported the estimations made using JEKF-SANTO and JEKF-KPH2 in regard to Xv, mAb, and QmAb of mAb production (run B-SD) using MRDE-PC and MRDE-PU with specific **P**(t = 0). In plot A of Figure 8 and Figure 9, we can see that all NSEs estimated the Xv close to the ground truth. However, the JEKF-Classic (purple line) was not able to evolve (update) the unshared parameter QmAb, because the estimations about QmAb were constant and equal to the initial value of 7.21 $\times \;10^{-9}\;$ mg cells ${\text{​}}^{-1}$ h ${\text{​}}^{-1}$ during the entire process. Consequently, the JEKF-Classic estimation regarding mAb was far from the ground truth (red dash line) of run B-SD (see plots B and C in Figure 8), and it had a high RMSPE value of 18.65%; see Table 1. The same results regarding the JEKF-Classic were obtained using MRDE-PU; see Figure 9. It is important to point out that the Kalman gain over time obtained by JEKF-Classic with SD is constant and equal to zero using MRDE-PU or MRDE-PC (see Figure 10). Furthermore, the Kalman gain values obtained by JEKF-SANTO with MRDE-PC were more stable than those obtained by JEKF-KPH2.

**Answer to RQ2-G2.** The results of JEKF-SANTO avoiding the JEKF failure (using runs B-SD ) can be seen in the plots B and C of Figure 8 and Figure 9. In these plots, we can see that JEKF-SANTO (blue line) evolved the QmAb from the initial value to the ground truth (red dash line) and consequently estimated the mAb close to the ground truth of run B-SD (red dash line) with MRDE-PU and MRDE-PC. These results are the opposite of the ones obtained with JEKF-Classic. In addition, JEKF-SANTO had the smallest RMSPE values between the NSEs in all cases; see Table 1. On the other hand, the JEKF-KPH2 did not perform similarly to JEKF-SANTO. The unique case where JEKF-KPH2 (green line) had a good performance was in run B-SD with MRDE-PU with specific **P**(t = 0). In that case, JEKF-KPH2 estimations were near to the ground truth (red dash line); see plots B and C of Figure 9. However, JEKF-KPH2 did not present stability, and the estimation converged to values far from the ground truth in run B-SD (with MRDE-PC with specific **P**(t = 0)). The best performances of JEKF-SANTO and JEKF-KPH2 were obtained by the use of specific **P**(t = 0) because when we used a standard **P**(t = 0) for JEKF-SANTO and JEKF-KPH2, their estimations are worse with runs B-SD. The results using standard **P**(t = 0) with runs B-SD can be found in Figures S15 and S16 and Table S12 of the Supplementary Material. These results (with standard and specific **P**(t = 0)) show that JEKF-KPH2 is sensitive to the initial P ${\text{​}}_{QmAb,QmAb}\left(t=0\right)$ , and JEKF-SANTO is sensitive to P ${\text{​}}_{X_{V},QmAb}\left(t=0\right)$ , since their better results were obtained with their specific **P**(t = 0). Table S4 of the Supplementary Material shows the specific **P**(t = 0) used in JEKF-KPH2, and Table S5 of the Supplementary Material shows the specific **P**(t = 0) used in JEKF-SANTO. It is important to point out that the best results of JEKF-SANTO were obtained with P ${\text{​}}_{X_{V},QmAb}\left(t=0\right)$ with positive values in case of run B-SD; see Table S5 of the Supplementary Material.

**Figure 11:**
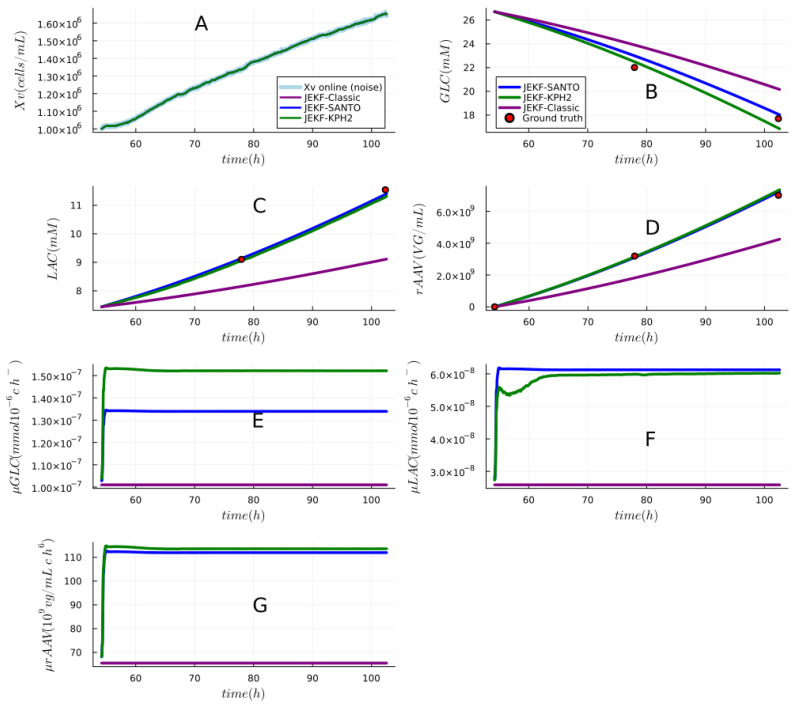
Simultaneous unshared parameters estimation by JEKF-SANTO and JEKF-KPH2 with real dataset (rAAV production). Plot (**A**) shows the estimations regarding Xv, and all estimations were inside of the noise range of the real online measurement of Xv by the capacitance probe. Plots (**B**–**D**) show the estimation obtained regarding the variables GLC, LAC, and rAAV, respectively. In these plots, we can see that JEKF-SANTO and JEKF-KPH2 had similar estimations. They evolved the $\mu_{GLC}$ , $\mu_{LAC}$ , and $\mu_{rAAV}$ (unshared parameters) with convergence, and their estimations related to GLC, LAC, and rAAV were close to the ground truth (red points); see plots (**E**–**G**). All NSEs were executed with MRDE-PC and specific **P**(t = 0).

**Figure 12:**
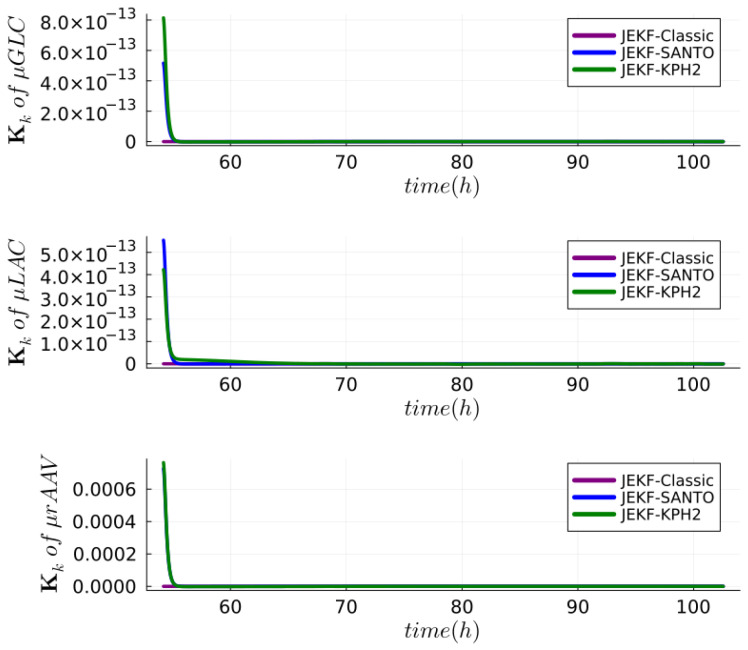
Kalmain gain over time for the NSEs with run B of real dataset. In all cases, the JEKF-Classic is constant and equal to zero.

**Answer to RQ3-G2.** In Figure 11, we show the estimations made by JEKF-SANTO and JEKF-KPH2 with regard to Xv, GLC, LAC, rAAV and the three unshared parameters ( $\mu_{GLC}$ , $\mu_{LAC}$ , and $\mu_{rAAV}$ ) of rAAV production (real dataset) using the MRDE-PC and the specific **P**(t = 0) and standard **Q**. In plot A of Figure 11, we can see that JEKF-SANTO and JEKF-KPH2 estimated the Xv inside of the noise range of the real online measurement of Xv by the capacitance probe. The following plots, B, C, and D, show the estimation obtained for the variables GLC, LAC, and rAAV. JEKF-SANTO (blue line) and JEKF-KPH2 (green line) were able to evolve the three unshared parameters simultaneously converging to values that enabled the estimation of GLC, LAC, and rAAV near the ground truth (red points in plots B, C and D). In these plots of Figure 11, and the RMSPE in Table 2, we can see that JEKF-SANTO and JEKF-KPH2 made similar estimations. Nevertheless, JEKF-SANTO had a slightly better performance than JEKF-KPH2 estimating GLC, LAC, and rAAV (titer); see the RMSPE Table 2. It is important to point out that the Kalman gain over time obtained by JEKF-Classic with RD is constant and equal to zero. See Figure 12. Consequently, JEKF-Classic had the worst performance and could not evolve the three unshared parameters simultaneously; see plots of Figure 11, and the RMSPE Table 2.

## 7. Discussion

Our theoretical and empirical results showed the JEKF failure with biomanufacturing conditions. These results showed that JEKF-Classic could not estimate the unshared parameters and the state simultaneously, since the Kalman gain related to the unshared parameter was constant and equal to zero from the beginning to the end of the processes tested. On the other hand, the results showed that the JEKF-SANTO and JEKF-KPH2 approaches can avoid the JEKF failure. However, the JEKF-SANTO had a more accurate estimation than JEKF-KPH2 while having faster and stable unshared parameters evolution to values that allowed the best performance of the process model tested. It is essential to point out that JEKF-SANTO performed best in two different situations, which were represented by run B-SD with MRDE-PC and MRDE-PC. The best performance of JEKF-KPH2 was only with run B-SD. Furthermore, the results showed that both approaches are sensitive to **P**(t = 0). JEKF-KPH2 is sensible to the P ${\text{​}}_{UP,UP}\left(t=0\right)$ , and JEKF-SANTO is sensible to P ${\text{​}}_{MSV,UP}\left(t=0\right)$ . It is essential to point out that the JEKF-SANTO approach did not change the probabilistic view of JEKF, and the minimization cost function in JEKF remained the same. Therefore, the JEKF-SANTO approach can be viewed as an artifact that prevents the Kalman gain from becoming zero with the biomanufacturing conditions (failure case). In addition, the JEKF-SANTO approach only addresses the failure case. It does not solve other issues, such as nonlinearity or high dimensionality, and should be used as a complementary approach. Beyond the SANTO approach, several methods have been established to tackle singularities and convergence issues in EKF. Rank reduction techniques address ill-conditioned covariance matrices by reducing their dimensionality, thus preventing singularities [63]. Time-correlated noise analysis allows for a more accurate state estimation by adjusting noise covariance matrices based on observed temporal correlations in system noise, providing a more realistic noise model [64]. These methods, used in conjunction with JEKF-SANTO, offer a comprehensive approach to EKF optimization in challenging scenarios like biomanufacturing. It is important to note that our analysis did not explicitly consider observability and stability conditions. However, this omission does not invalidate our study. The focus of our research was on addressing a specific failure case of JEKF under certain biomanufacturing conditions. Our proposed solution, SANTO, was developed to specifically address this issue based on experiments with JEKF that are consistent. Therefore, in our study, while we did not explicitly detail the observability and stability analysis in the traditional sense, we implicitly addressed these aspects through empirical evaluation methods. Regarding observability, our approach primarily focused on the empirical performance of the JEKF in the given case study rather than a formal observability analysis.

## 8. Conclusions

In this work, firstly, we presented the common conditions in biomanufacturing that represent a failure case for the classical JEKF. Secondly, we proved that the classical JEKF, with these conditions, cannot estimate the unshared parameters and the state simultaneously since the Kalman gain related to the unshared parameter is constant and equal to zero in the entire process. Lastly, we presented an approach called SANTO, which is a simple and effective way to address the JEKF failure case by adding a positive quantity ( λ ) regarding the initial state error covariance between a measured state variable and an unshared parameter (P ${\text{​}}_{MSV,UP}\left(t=0\right)$ ) in **P**(t = 0). Our empirical evaluation demonstrated that the SANTO approach effectively estimates unshared parameters and states simultaneously, aligning closely with ground truth values in the tested datasets. SANTO notably outperformed both JEKF-Classic and JEKF-KPH2 in accuracy. In a rigorously controlled test using a synthetic dataset, JEKF-SANTO, whether paired with MRDE-PC or MRDE-PU, exhibited a substantial improvement in RMSPE, achieving up to approximately 17% enhancement compared to JEKF-Classic. Meanwhile, JEKF-KPH2 showed an improvement of around 8.7% in RMSPE, but this was limited to its execution with MRDE-PU. This highlights the effectiveness of SANTO in overcoming the limitations of classical JEKF in biomanufacturing applications. Our future works will focus on the development of an auto-tuning mechanism based on an objective function to systematically calibrate **Q**, **R** and λ , as seen in [65], but also investigate the potential of the Unscented Kalman Filter (UKF) to estimate the unshared parameters and the state simultaneously with the biomanufacturing conditions.

## Figures and Tables

**Table 1:** RMSPE between NSEs estimations about mAb and ground truth of run B in synthetic dataset with specific **P**(t = 0).

NSE	RMSPE (MRDE-PU)	RMSPE (MRDE-PC)
JEKF-SANTO	2.06%	1.30%
JEKF-KPH2	10.11%	48.44%
JEKF-Classic	18.80%	18.65%

**Table 2:** RMSPE between NSEs estimations and ground truth of real dataset with MRDE-PC, specific **P**(t = 0) and (standard **Q**).

Ground Truth	JEKF-Classic	JEKF-SANTO	JEKF-KPH2
GLC	11.6%	3.48%	3.58%
LAC	16.66%	1.01%	1.61%
rAAV (titer)	41.41%	2.87%	3.28%

## Data Availability

The data and code used in this study are available in github https://github.com/cristovaoiglesias/JEKF-SANTO (accessed on 20 November 2023).

## Supplementary Materials

The following supporting information can be downloaded at: www.mdpi.com/xxx/s1, Figure S1: Innovation Magnitude Bound Test using the run B of Synthetic dataset for the NSEs with MRDE-PU and specific **Q** and **P**(0); Figure S2: Innovation Magnitude Bound Test using the run B of Synthetic dataset for the NSEs with MRDE-PC and standard **Q** and **P**(0); Figure S3: Innovation Magnitude Bound Test using the run B of Synthetic dataset for the NSEs with MRDE-PU and standard **Q** and **P**(0); Figure S4: Standard Error of $X_{V}$ at each k estimated by NSEs with Synthetic Dataset using MRDE-PU and specific P(0); Figure S5: Standard Error of QmAb at each k estimated by NSEs with Synthetic Dataset using MRDE-PC and specific P(0); Figure S6: Standard Error of QmAb at each k estimated by NSEs with Synthetic Dataset using MRDE-PU and specific P(0); Figure S7: Standard Error of mAb at each k estimated by NSEs with Synthetic Dataset using MRDE-PC and specific P(0); Figure S8: Standard Error of mAb at each k estimated by NSEs with Synthetic Dataset using MRDE-PU and specific P(0); Figure S9: Standard Error of GLC at each k estimated by NSEs with Real Dataset using MRDE-PC and specific P(0); Figure S10: Standard Error of LAC at each k estimated by NSEs with Real Dataset using MRDE-PC and specific P(0); Figure S11: Standard Error of rAAV at each k estimated by NSEs with Real Dataset using MRDE-PC and specific P(0); Figure S12: Standard Error of μGLC at each k estimated by NSEs with Real Dataset using MRDE-PC and specific P(0); Figure S13: Standard Error of μLAC at each k estimated by NSEs with Real Dataset using MRDE-PC and specific P(0); Figure S14: Standard Error of μrAAV at each k estimated by NSEs with Real Dataset using MRDE-PC and specific P(0); Figure S15: The JEKF-SANTO and JEKF-KPH2 avoid the JEKF failure in B-SD, but they need an specific P(t=0) . First, plot A shows the estimations regards Xv, and all estimations were close the ground truth. The plots B and C show the estimations regards the unshared parameter QmAb and mAb (titer) far from the ground truth, respectively. The NSEs were executed with **MRDE-PC** and **standard P(t=0) **; Figure S16: The JEKF-SANTO and JEKF-KPH2 avoid the JEKF failure in B-SD, but they need an specific P(t=0) . First, plot A shows the estimations regards Xv, and all estimations were close the ground truth. The plots B and C show the estimations regards the unshared parameter QmAb and mAb (titer) far from the ground truth, respectively. The NSEs were executed with **MRDE-PU** and **standard P(t=0) .**; Table S1: Parameters used in UMM case 1.4 to generate the runs A-SD, and B-SD of Synthetic Dataset (SD); Table S2: Initial conditions of state variables of UMM case 4 for the JEKF test with Synthetic Dataset; Table S3: Standard initial state error covariance matrix (standard **P**(t = 0)) for JEKF-Classic, JEKF-KPH2 and JEKF-SANTO with run B of Synthetic Dataset; Table S4: Specific initial state error covariance matrix (specific **P**(t = 0)) for JEKF-KPH2 with run B of Synthetic Dataset; Table S5: Specific initial state error covariance matrix (specific **P**(t = 0)) for JEKF-SANTO with run B of Synthetic Dataset; Table S6: Measurement noise variance **R** and error covariance matrix of process model (**Q**) for the JEKF-Classic, JEKF-SANTO and JEKF-KPH2 with run B of Synthetic Dataset using MRDE-PC; Table S7: Measurement noise variance **R** and error covariance matrix of process model (**Q**) for the JEKF-Classic, JEKF-SANTO and JEKF-KPH2 with run B of Synthetic Dataset using MRDE-PU; Table S8: Initial conditions of state variables of UMM case 5 for the JEKF-SANTO and JEKF-KPH2 test with run B-RD (Source [19]); Table S9: Initial parameters obtained with A-RD for the JEKF-SANTO and JEKF-KPH2 test with run B-RD (Source [19]); Table S10: Specific initial state error covariance matrix (specific **P**(t = 0)) for for the JEKF-Classic, JEKF-SANTO and JEKF-KPH2 with Real Dataset (run B) using MRDE-PC; Table S11: Measurement noise variance **R**, and error covariance matrix of process model $Q_{i,i}$ for the JEKF-Classic, JEKF-SANTO and JEKF-KPH2 with Real Dataset (run B) using MRDE-PC; Table S12: RMSPE between NSEs estimations about mAb and ground truth of run B in synthetic dataset with standard**P**(t = 0).

## Abbreviations

The following abbreviations are used in this manuscript:

JEKF	Joint estimation of states and parameters with Extended Kalman Filter
NSE	Nonlinear State Estimator
UMM	Unstructured Mechanistic Model
SMM	Structured Mechanistic Model
MRDE	Matrix Ricatti Differential Equation
MSV	Measured State Variable
UP	Unshared parameter
SANTO	Specific initiAl coNdiTiOn
CD-EKF	Continuous-Discrete EKF
RD	Real dataset
SD	Synthetic dataset
rAAV	Recombinant Adeno-Associated Virus
mAb	Monoclonal Antibody
