# Supplementary material for: How Not to Make the Joint Extended Kalman Filter Fail with Unstructured Mechanistic Models
Source: Sensors (Basel). 2024 Jan 19;24(2):0. doi: 10.3390/s24020653 (PMC11154378; doi:10.3390/s24020653)
Supplement: Supplementary file 1 [file sensors-24-00653-s001.zip › sensors-2759253-supplementary.pdf]

Supplementary Materials: How NOT To Make the Joint Extended Kalman Filter Fail with Unstructured Mechanistic Models

Cristovao Freitas Iglesias Jr [1] and Miodrag Bolic [1,*]

Contents

1. Unstructured Mechanistic Models cases in biomanufacturing . . . 2
1.1. Case 1 . . . 2
1.2. Case 2 . . . 2
1.3. Case 3 . . . 3
1.4. Case 4 . . . 3
1.5. Case 5 . . . 3

2. Continuous-Discrete Extended Kalman Filter . . . 4
2.1. Intuitions behind Kalman Gain and unshared parameters . . . 6

3. Analysis of Unstructured Mechanistic Models . . . 7
3.1. Unshared and shared parameters . . . 7
3.2. Weak and strong terms . . . 7
3.3. Weak and strong variables . . . 8
3.4. MRDE to predict the state error covariance $\mathbf{P}(t_{k|k-1})$ based on $\mathbf{P}$ and $\mathbf{Q}$ with uncorrelated elements . . . 8
3.5. Only one variable measured . . . 9

4. Example of the Lemma: Inability to Update Kalman Gain for Unshared parameters 9

5. Example of the Theorem (JEKF failure) . . . 12

6. Related work: Approach KPH2 . . . 12

7. Empirical Evaluation - Extension . . . 15
7.1. Synthetic dataset development - mAb production . . . 15
7.2. Real dataset development - rAAV production . . . 17
7.3. NSEs (JEKF-classic, JEKF-SANTO and JEKF-KPH2) design to address RQ1-G1 and RQ2-G2 . . . 17
7.4. NSEs (JEKF-SANTO and JEKF-KPH2) design to address RQ3-G2 . . . 25
7.5. Results with synthetic dataset . . . 33
7.6. Codes and datasets availability . . . 35

8. References . . . 35

1. Unstructured Mechanistic Models cases in biomanufacturing

1.1. Case 1

The ODE system 1 represents the dynamics of mammalian cell growth $Xv(t)$, nutrients $N(t)$ and metabolite/production formation $MP(t)$ in a general form [1,2].

$$\begin{aligned}
\frac{dX_V(t)}{dt} &= \mu_{X_v} X_V(t) \\
\frac{dN(t)}{dt} &= -\mu_N X_V(t) \\
\frac{dMP(t)}{dt} &= \mu_{mp} X_V(t)
\end{aligned} \tag{1}$$

1.2. Case 2

The ODE system 2 is a UMM used for Mab production [3]. This system represents the cell growth, cell dead, uptake of substrates, metabolism, and production process with 35 parameters. More details can be found in [3].

$$\begin{aligned}
\frac{d\,fgr}{dt} &= -k_{11} \frac{fgr}{1 + ([GLC]/K_{12})} \\
\frac{d\,X_V}{dt} &= \mu_{max}.fgr.X_V.\frac{[GLC]}{K_{21} + [GLC]}.\frac{[GLN]}{K_{22} + [GLN]}.\frac{1}{[LAC]/K_{24} + 1}.\frac{1}{[AMM]/K_{23} + 1} - C \\
C &= k_d.(1 - fgr).X_V.(\frac{1}{1 + ([AMM]/k_{25})^n} + \frac{k_{26}}{[GLC]}) \\
\frac{d\,X_d}{dt} &= k_d.(1 - fgr).X_V.(\frac{1}{1 + ([AMM]/k_{25})^n} + \frac{k_{26}}{[GLC]}) - k_{lys}.X_d \\
\frac{d\,[GLC]}{dt} &= -(\frac{K_{31}.[GLC].[GLN]}{(k_{32} + [GLC])(k_{33} + [GLN])} + \frac{K_{34}.[GLC]}{(k_{35} + [GLC])}).X_V - k_{36} X_V \\
\frac{d\,[GLN]}{dt} &= -k_{41}.X_V.(\frac{[GLC].[GLN]}{(k_{42} + [GLC])(k_{43} + [GLN])}) \\
\frac{d\,[LAC]}{dt} &= -k_{51}.X_V.\frac{d\,[GLC]}{dt} \\
\frac{d\,[ASN]}{dt} &= -k_{61}.X_V.\frac{[ASN]}{(k_{62} + [ASN])} \\
\frac{d\,[ASP]}{dt} &= X_V.(\frac{K_{61}[ASN]}{K_{62} + [ASN]} - \frac{K_{63}[ASP]}{K_{64} + [ASP]} + \frac{K_{65}.[GLC].[GLN]}{(K_{66} + [GLC])(K_{67} + [GLN])}) \\
\frac{d\,[ALA]}{dt} &= X_V.(\frac{K_{65}.[GLC].[GLN]}{(K_{66} + [GLC])(K_{67} + [GLN])} - \frac{K_{68}[ALA]}{K_{69} + [ALA]}) \\
\frac{d\,[AMM]}{dt} &= -k_{71}.\frac{d\,[GLN]}{dt} + k_{72}.X_V.(\frac{K_{61}[ASN]}{K_{62} + [ASN]} + \frac{K_{63}[ASP]}{K_{64} + [ASP]} + \frac{K_{68}.[ALA]}{(K_{69} + [ALA])}) \\
\frac{d\,[mAb]}{dt} &= X_V(k_{81} + k_{82}.[GLC])
\end{aligned} \tag{2}$$

1.3. Case 3

The ODE system 3 represents the Michaelis-Menten model for enzymatic reactions [4].

$$\begin{aligned}
\frac{dE(t)}{dt} &= -k_1 E(t)S(t) + k_2 ES(t) + k_3 ES(t) \\
\frac{dS(t)}{dt} &= -k_1 E(t)S(t) + k_2 ES(t) \\
\frac{dES(t)}{dt} &= k_1 E(t)S(t) - k_2 ES(t) - k_3 ES(t) \\
\frac{dP(t)}{dt} &= k_3 ES(t)
\end{aligned} \tag{3}$$

1.4. Case 4

The ODE system 4 is a UMM used for Mab production [5]. This system represents the cell growth, uptake of substrates, metabolism, and production process with 16 parameters described in the Table S1. It is important to point out that $QmAb$ denotes the specific mAb production rate, and is an example of unshared parameter. More details can be found in [5].

$$\begin{aligned}
\frac{d\,X_V}{dt} &= (\mu - \mu_d)X_V \\
\frac{d\,X_t}{dt} &= \mu X_V - k_{lysis}(X_t - X_V) \\
\mu &= \mu_{max} \cdot \frac{[GLC]}{K_{glc} + [GLC]} \cdot \frac{[GLN]}{K_{gln} + [GLN]} \cdot \frac{K_{Ilac}}{K_{Ilac} + [LAC]} \cdot \frac{K_{Iamm}}{K_{Iamm} + [AMM]} \\
\mu_d &= \frac{\mu_{d,max}}{1 + (K_{d,amm} + [AMM])^2} \\
\frac{d\,[GLC]}{dt} &= -Q_{glc} Xv \\
\frac{d\,[GLN]}{dt} &= -Q_{gln} Xv - K_{d,gln}[GLN] \\
\frac{d\,[LAC]}{dt} &= Q_{lac} Xv \\
\frac{d\,[AMM]}{dt} &= Q_{amm} Xv + K_{d,gln}[GLN] \\
Q_{glc} Xv &= \frac{\mu}{Y_{x,glc}} + m_{glc} \\
Q_{gln} Xv &= \frac{\mu}{Y_{x,gln}} + m_{gln} = \frac{\mu}{Y_{x,gln}} + \frac{\alpha_2 [GLN]}{\alpha_2 + [GLN]} \\
Q_{lac} Xv &= Y_{lac,glc} Q_{glc} \\
Q_{amm} Xv &= Y_{amm,gln} Q_{gln} \\
\frac{d\,[mAb]}{dt} &= (2 - \gamma\mu) Q_{mAb} . X_V
\end{aligned} \tag{4}$$

1.5. Case 5

An UMM used to monitoring rAAV production [6] is the following

$$\begin{aligned}
\frac{dX_V(t)}{dt} &= \mu_{X_v} X_V(t) \\
\frac{dGlc(t)}{dt} &= -\mu_{Glc} X_V(t) \\
\frac{dGln(t)}{dt} &= -\mu_{Gln} X_V(t) \\
\frac{dLac(t)}{dt} &= \mu_{Lac} X_V(t) \\
\frac{dAmm(t)}{dt} &= \mu_{Amm} X_V(t) + k_{deg} Gln(t) \\
\frac{dAAV(t)}{dt} &= \mu_{AAV} X_V(t)
\end{aligned} \tag{5}$$

This system represents the cell growth, uptake of substrates, metabolism, and production process with six parameters: the specific cell growth rate (μ_{X_v}), the specific rates of uptake (consumption) of the main nutrients, glucose (μ_{Glc}) and glutamine (μ_{Gln}), the specific rates of production of the metabolite waste, lactate (μ_{Lac}) and ammonium (μ_{Amm}), and specific rate of production of rAAV(μ_{AAV}). In the case of ammonium production, the specific rates must consider the spontaneous glutamine degradation in the medium into ammonium. This process follows first-order rate kinetics concerning glutamine concentration, being k_{deg} the glutamine degradation constant.

2. Continuous-Discrete Extended Kalman Filter

The EKF can be implemented in Discrete-Discrete, and Continuous-Discrete versions [7–9]. The most common version used in biomanufacturing for modeling nonlinear biochemical dynamic pathways is Continuous-Discrete EKF (CD-EKF) [3,10–12] and we will describe it here. The EKF requires a state–space model to perform estimation on the state variables of a process (nonlinear system) present in a state variable vector $\boldsymbol{\psi}(t)$ [4,13,14]. A state-space model consists of process and measurement (observation) models [15]. EKF linearizes the nonlinear system (state–space model) by calculating the Jacobians of the nonlinear process and measurement models based on the first-order Taylor series expansion in order to analytically propagate the Gaussian random-variable representation [11,14,16].

Effectively, the nonlinear dynamics are approximated by a time-varying linear system, and the linear Kalman filters equations are applied. Essentially, the mean $\hat{\boldsymbol{\psi}}(t)$ and error covariance matrix $\mathbf{P}(t)$ of the state variables in $\boldsymbol{\psi}(t)$ are recursively corrected. The EKF recursively estimates the (posterior) mean $\hat{\boldsymbol{\psi}}(t_{k/k})$ and error covariance matrix $\mathbf{P}(t_{k/k})$ of the state variables by combining the predicted (a priori) mean $\hat{\boldsymbol{\psi}}(t_{k/k-1})$ and error covariance matrix $\mathbf{P}(t_{k/k-1})$ with the current noisy measurement Z_k [16].

Process Model: An UMM as described in the supplementary information Section 1 can be used as the process model of EKF. The state variables vector to be used by the EKF is composed of the state variables of the UMM (observed and unobserved) and the state variables vector is defined as:

$$\boldsymbol{\psi}(t) = [x_1, x_2, ..., x_n]^T. \tag{6}$$

Subsequently, the process model is represented as

$$\frac{d\boldsymbol{\psi}(t)}{dt} = \phi(\boldsymbol{\psi}(t), t) + \boldsymbol{\omega}(t), \tag{7}$$

where ϕ denotes non-linear functions of the state variables in $\boldsymbol{\psi}(t)$, which corresponds to an UMM. The process model is formulated in a continuous time t and the white process noise vector is represented by $\boldsymbol{\omega} \sim \mathcal{N}(0, \mathbf{Q})$, with zero mean and error covariance matrix of process model represented by $\mathbf{Q}$.

Measurement Model: The measurement model is treated as a discrete system and defined as

$$\mathbf{Z}_k = h(\boldsymbol{\psi}(t_k)) + \boldsymbol{v}. \tag{8}$$

The non-linear function h in the measurement model relates the current state variables to the measurements $\mathbf{Z}_k$. The white measurement noise vector is represented by $\boldsymbol{v} \sim \mathcal{N}(0, \mathbf{R})$, with zero mean and measurement noise variance represented by $\mathbf{R}$. When the some state variables can be measured directly, we have a simple case and h can be a linear model. If h is linear, we have $h(\boldsymbol{\psi}(t_k)) = \mathbf{H}\boldsymbol{\psi}(t_k)$ [3,4,11]. Where the matrix $\mathbf{H}$ is a linear operator (row vector) that matches the states variables of $\boldsymbol{\psi}(t_k)$ to the measured variables $\mathbf{Z}_k$ that are obtained at a discrete instance k [3,11]. Consequently, the measurement model (8) can be rewritten as

$$\mathbf{Z}_k = \mathbf{H}\boldsymbol{\psi}(t_k) + \boldsymbol{v}. \tag{9}$$

Probabilistic state-space models: The *probabilistic process model* approximated by EKF is

$$p(\boldsymbol{\psi}(t)|\boldsymbol{\psi}(t-1)) \approx \mathcal{N}(\boldsymbol{\psi}(t)|\phi(\boldsymbol{\psi}(t-1)), \mathbf{Q}), \tag{10}$$

where the non-linear functions ϕ are linearized as follows

$$\begin{aligned}\phi(\boldsymbol{\psi}(t-1)) \approx \phi(\hat{\boldsymbol{\psi}}(t-1)) + \\ \mathbf{J}_t^{\phi} \times (\boldsymbol{\psi}(t-1) - \hat{\boldsymbol{\psi}}(t-1))\end{aligned} \tag{11}$$

and $\mathbf{J}_t^{\phi}$ is the Jacobian matrix of ϕ evaluated at the prior mode [17,18],

$$\mathbf{J}_t^{\phi} = \left.\frac{\partial \phi(\boldsymbol{\psi}(t))}{\partial \boldsymbol{\psi}_i}\right|_{\boldsymbol{\psi}(t)=\hat{\boldsymbol{\psi}}(t-1)}. \tag{12}$$

The *probabilistic measurement model* (measurement likelihood distribution) approximated by EKF is the following

$$p(\mathbf{Z}_k|\boldsymbol{\psi}(t_k)) \approx \mathcal{N}(\mathbf{Z}_k|h(\boldsymbol{\psi}(t_k)), \mathbf{R}) \tag{13}$$

where the non-linear functions h are linearized as follows

$$\begin{aligned}h(\boldsymbol{\psi}(t_k)) \approx h(\hat{\boldsymbol{\psi}}(t_k)) + \\ \mathbf{J}_t^{h} \times (\boldsymbol{\psi}(t_k) - \hat{\boldsymbol{\psi}}(t_k))\end{aligned} \tag{14}$$

and the $\mathbf{J}_{t_k}^{h}$ is the Jacobian matrix of h evaluated at the prior mode [17,18]

$$\mathbf{J}_{t_k}^{h} = \left.\frac{\partial h(\boldsymbol{\psi}(t_k))}{\partial \boldsymbol{\psi}}\right|_{\boldsymbol{\psi}(t)=\hat{\boldsymbol{\psi}}(t_{k/k-1})}. \tag{15}$$

In this work, we will consider the case of h be linear. Then, we have

$$\mathbf{J}_{t_k}^{h} = \left.\frac{\partial (\mathbf{H}\boldsymbol{\psi}(t_k))}{\partial \boldsymbol{\psi}}\right|_{\boldsymbol{\psi}(t)=\hat{\boldsymbol{\psi}}(t_{k/k-1})} = \mathbf{H}\left.\frac{\partial (\boldsymbol{\psi}(t_k))}{\partial \boldsymbol{\psi}}\right|_{\boldsymbol{\psi}(t)=\hat{\boldsymbol{\psi}}(t_{k/k-1})} = \mathbf{H} \tag{16}$$

and consequently

$$p(\mathbf{Z}_k|\boldsymbol{\psi}(t_k)) \approx \mathcal{N}(\mathbf{Z}_k|\mathbf{H}\hat{\boldsymbol{\psi}}(t_k) + \mathbf{H} \times (\boldsymbol{\psi}(t_k) - \hat{\boldsymbol{\psi}}(t_k)), \mathbf{R}) \tag{17}$$

or

$$p(\mathbf{Z}_k|\boldsymbol{\psi}(t_k)) \approx \mathcal{N}(\mathbf{Z}_k|\mathbf{H}\hat{\boldsymbol{\psi}}(t_{k/k-1}) + \mathbf{H} \times (\boldsymbol{\psi}(t_{k/k-1}) - \hat{\boldsymbol{\psi}}(t_{k/k-1})), \mathbf{R}). \quad (18)$$

EKF algorithm: The EKF algorithm is implemented through the initial condition, prediction step (time update) and correction step (measurement update) [3,4,11–13].

Initialization step: The initial condition are composed of the initial mean $\hat{\boldsymbol{\psi}}_0 = E[\boldsymbol{\psi}_0]$, and initial error covariance matrix $\mathbf{P}_0 = \mathbf{P}_{i,i}(t=0) = E[(\boldsymbol{\psi}_0 - \hat{\boldsymbol{\psi}}_0)(\boldsymbol{\psi}_0 - \hat{\boldsymbol{\psi}}_0)^T]$ of state variables vector [16].

Prediction step: In this step, the *a priori* predictions represented by the predicted mean $\hat{\boldsymbol{\psi}}(t_{k/k-1})$ and predicted error covariance matrix $\mathbf{P}(t_{k|k-1})$ of state variables vector $\boldsymbol{\psi}(t)$ are obtained respectively by numerically integrating $\phi(\boldsymbol{\psi}(t), t)$ from discrete time t_{k-1} to t_k the following equation

$$\hat{\boldsymbol{\psi}}(t_{k/k-1}) = \hat{\boldsymbol{\psi}}(t_{k-1}) + \int_{t_{k-1}}^{t_k} \phi(\hat{\boldsymbol{\psi}}(t))dt\Big|_{\hat{\boldsymbol{\psi}}(t_{k-1})} \quad (19)$$

and solving the matrix Riccati Differential equation (MRDE) for predict the state error covariance matrix [7,19]

$$\frac{d\mathbf{P}(t)}{dt} = \mathbf{J}_t^{\phi}\mathbf{P}(t) + \mathbf{P}(t)\mathbf{J}_t^{\phi T} + \mathbf{Q} \quad (20)$$

from t_{k-1} to t_k, where a new measurement is obtained at time k [20], [7] and [21]. The Equation 20 is basically a matrix of ODEs, and the matrix of ODEs solutions obtained from t_{k-1} to t_k represent each state error covariance of the system. See the MRDE represented by Equation 37 and the respective solution represented by Matrix 40 in the Section 4 of Supplementary Material.

Correction step: In this step, the results of the prediction step ($\hat{\boldsymbol{\psi}}(t_{k/k-1})$ and $\mathbf{P}(t_{k|k-1})$) are combined with the measured value $\mathbf{Z}_k$ and Kalman gain ($\mathbf{K}_k$) to provide the estimated mean $\hat{\boldsymbol{\psi}}(t_{k/k})$ and estimated error covariance matrix $\mathbf{P}(t_{k|k})$ of state variables vector using the following equations:

$$\mathbf{K}_k = \mathbf{P}(t_{k|k-1})\mathbf{H}^T(\mathbf{H}\mathbf{P}(t_{k|k-1})\mathbf{H}^T + \mathbf{R})^{-1} \quad (21)$$

$$\hat{\boldsymbol{\psi}}(t_{k/k}) = \hat{\boldsymbol{\psi}}(t_{k/k-1}) + \mathbf{K}_k(\mathbf{Z}_k - \mathbf{H}\hat{\boldsymbol{\psi}}(t_{k/k-1})) \quad (22)$$

$$\mathbf{P}(t_{k|k}) = (\mathbf{I} - \mathbf{K}_k\mathbf{H})\mathbf{P}(t_{k|k-1}) \quad (23)$$

The Kalman gain is a scaling factor (ratio) to estimate the state variables by setting a value between the predicted state and measured state [7,22]. The $\mathbf{K}_k$ chooses a value along the residual range ($\mathbf{Z}_k$ - $\mathbf{H}\hat{\boldsymbol{\psi}}(t_{k/k-1})$)[16,22]. $\mathbf{K}_k$ enables to set a value for $\hat{\boldsymbol{\psi}}(t_{k/k})$ between the $\hat{\boldsymbol{\psi}}(t_{k/k-1})$ (prediction) and $\mathbf{Z}_k$ (measurement) using Equation 22, and update the belief regards the state variables based on how certain we are regards the measurement using the Equation 23, [22] (pages 137 and 209). The Kalman gain is computed as a ratio of prior and measurement uncertainty available; see Equation 21. The one dimensional form Equation 21 is the following $K = P/(P+R)$ [22]. It is important to point out that linear operator $\mathbf{H}$ matches the states variables of $\boldsymbol{\psi}(t_k)$ to the measured variables $\mathbf{Z}_k$ that are obtained at a discrete instance. It is linear operator with zeros and ones. Where the elements ones represent elements of the state variable vector that are measured. For example, if the state variables vector has 3 variables and only the first one is measured, we have $\mathbf{H}$ = [1 0 0].

Using the estimated mean $\hat{\boldsymbol{\psi}}(t_{k/k})$ and estimated error covariance matrix $\mathbf{P}(t_{k|k})$ state variables vector as an initial condition, we can return to the prediction step until the next measurement be obtained and everything repeated again.

2.1. Intuitions behind Kalman Gain and unshared parameters

Analyzing the Equation 21 is possible to have the following approximation [22,23]

$$\mathbf{K}_k \approx \frac{Process_{\ Uncertainty}}{System_{\ Uncertainty}}, \tag{24}$$

and extract the following two interpretations [22]: **First**, When the Process uncertainty is large (nominator in Equation 24), K is large and so the corrections (Equation 22) are almost determined by the influence of the measured state variable. Since, $\mathbf{K}$ is multiplied by the residual $(\mathbf{Z}_k - \mathbf{H}\hat{\boldsymbol{\psi}}(t_{k/k-1}))$. So, a large K favors the measurement [24]; and **Second**, On the other hand, if the Process uncertainty is very low, the correction step is almost the estimated without influence of the measurement obtained. Since, $\mathbf{K} \approx 0$ and $\hat{\boldsymbol{\psi}}(t_{k/k}) = \hat{\boldsymbol{\psi}}(t_{k/k-1})$ [23].

3. Analysis of Unstructured Mechanistic Models

3.1. Unshared and shared parameters

The UMM case 1.1 is the case of an ODE system with only **unshared parameters**. On another hand, the UMM case 1.3 is a ODE system with only **shared parameters**. For example, the k_3 is used in different ODEs of this system. The UMM case 1.2 is a case of system with unshared and shared parameters.

3.2. Weak and strong terms

The three UMM cases (1.1, 1.2 and 1.3) presented above are examples of ODE systems with weak and strong terms. The UMM case 1.1 is example of ODE system with only terms that could be considered weak, because they have low percentage of variables that compose the state variable vector. For example, let assume the following state variable vector $\psi(t)_{case1} = [Xv, N, MP, \mu_{Xv}, \mu_N, \mu_{mp}]$ with six elements for the UMM of case 1.1. We have that all terms of this UMM has a $1/3$ of the state variable vector. On another hand, the UMM case 1.2 is example of ODE system with terms that could be considered weak and strong. For example, let consider the following state variable vector for UMM case 1.2,

$$\psi_{case2} = [X_v, fgr, X_d, GLC, GLN, LAC, ASN, ASP, ALA, AMM, Mab, \mu_{max}, k_{31}, k_d] \tag{25}$$

we have that the first term of equation $\frac{dX_v}{dt}$ is the strongest term in this system, since it has $7/14$ of state variable vector, and the first term of the equation $\frac{dMab}{dt}$ as the weakest term, since it has $2/14$ of state variable vector. In the context of JEKF, we have that a strong term in an UMM contribute more than weak term to compute of predicted state error covariance $\mathbf{P}(t_{k|k-1})$. Since, many elements of Jacobian $\mathbf{J}_t^{\phi}$, results from the first-order partial derivatives of strong term with respect to the variables of state variable vector $\psi(t)$. For example, let consider the following:

- State variable vector $\psi(t) = [x_1, x_2, x_3, x_4]$
- An UMM composed of an ODE with a strong term by the function S(x1,x2,x3,x4) and three ODEs composed of waek terms represented by the function $W_1(x_1)$, $W_2(x_2)$ and $W_3(x_3)$.
- MRDE (Equation 20).
- **P** and **Q** uncorrelated for the $\psi(t)$,

$$\mathbf{P} = \begin{bmatrix} P_{1,1} & 0 & 0 & 0 \\ 0 & P_{2,2} & 0 & 0 \\ 0 & 0 & P_{3,3} & 0 \\ 0 & 0 & 0 & P_{4,4} \end{bmatrix}, \mathbf{Q} = \begin{bmatrix} Q_{1,1} & 0 & 0 & 0 \\ 0 & Q_{2,2} & 0 & 0 \\ 0 & 0 & Q_{3,3} & 0 \\ 0 & 0 & 0 & Q_{4,4} \end{bmatrix}. \tag{26}$$

Given this we have the following Jacobian

$$\mathbf{Jacobian}(S, W_1, W_2, W_3) = \begin{bmatrix} \frac{\partial S}{\partial x_1} & \frac{\partial S}{\partial x_2} & \frac{\partial S}{\partial x_3} & \frac{\partial S}{\partial x_4} \\ \frac{\partial W_1}{\partial x_1} & 0 & 0 & 0 \\ 0 & \frac{\partial W_2}{\partial x_2} & 0 & 0 \\ 0 & 0 & \frac{\partial W_3}{\partial x_3} & 0 \end{bmatrix} \quad (27)$$

and the following MRDE to compute the Predicted state error covariance $\mathbf{P}(t_{k/k-1})$ from t_{k-1} to t_k,

$$\frac{d\mathbf{P}(t)}{dt} = \begin{bmatrix} Q_{1,1} + 2.P_{1,1}.\frac{\partial S}{\partial x_1} & P_{1,1}.\frac{\partial W_1}{\partial x_1} + P_{2,2}.\frac{\partial S}{\partial x_2} & P_{3,3}.\frac{\partial S}{\partial x_3} & P_{4,4}.\frac{\partial S}{\partial x_4} \\ P_{1,1}.\frac{\partial W_1}{\partial x_1} + P_{2,2}.\frac{\partial S}{\partial x_2} & Q_{2,2} & P_{2,2}.\frac{\partial W_2}{\partial x_2} & 0 \\ P_{3,3}.\frac{\partial S}{\partial x_3} & P_{2,2}.\frac{\partial W_2}{\partial x_2} & Q_{3,3} & P_{3,3}.\frac{\partial W_3}{\partial x_3} \\ P_{4,4}.\frac{\partial S}{\partial x_4} & 0 & P_{3,3}.\frac{\partial W_3}{\partial x_3} & Q_{4,4} \end{bmatrix}. \quad (28)$$

Then, we can see that S contribute with 7 partial derivatives and the others functions with 2 partial derivative only each one. If we consider the MRDE formed with $\mathbf{P}$ correlated, we have S contributing with 32 partial derivatives and the others functions with 8 partial derivative only each one. It is important point out that the element 0 in the MRDE (Equation 28) represents an time invariant ODE $\frac{dP_{x_2,x_4}(t_{k/k-1})}{dt} = 0$ to predicts the covariance between x_4 and x_2 when solved from t_{k-1} to t_k.

3.3. *Weak and strong variables*

The three UMM cases (1.1, 1.2 and 1.3) presented above are examples of the ODE system with weak and strong variables. The variable *MP* in the UMM case 1.1 and *Mab* in UMM case 1.2 are examples of weak variable. In these cases, the first-order partial derivatives of all functions with respect to these two variables are equal to zero and this reflects that the variable has a column with zeros in the jacobian $\mathbf{J}_t^{\phi}$. On another hand, in the UMM case 1.1 and 1.2, *Xv* is an example of strong variable.

3.4. *MRDE to predict the state error covariance $\mathbf{P}(t_{k|k-1})$ based on* **P** *and* **Q** *with uncorrelated elements*

For any UMM, the use of **P** and **Q** with uncorrelated elements in MRDE, means that the predicted state error covariance $\mathbf{P}(t_{k|k-1})$ will be updated/calculated based only in noise variance of $P_{i,i}$ and $Q_{i,i}$ and elements of Jacobian $\mathbf{J}_t^{\phi}$.

For example, let consider the following conditions:

- The set of functions $\mathbf{f} = [f_1, f_2, f_3, f_4, f_5]$ and state variables vector $\psi(t)_{\mathbf{f}} = [x_1, x_2, x_3, x_4, x_5]$.
- P and Q uncorrelated for the $\psi(t)_{\mathbf{f}}$,

$$\mathbf{P} = \begin{bmatrix} P_{1,1} & 0 & 0 & 0 & 0 \\ 0 & P_{2,2} & 0 & 0 & 0 \\ 0 & 0 & P_{3,3} & 0 & 0 \\ 0 & 0 & 0 & P_{4,4} & 0 \\ 0 & 0 & 0 & 0 & P_{5,5} \end{bmatrix}, \mathbf{Q} = \begin{bmatrix} Q_{1,1} & 0 & 0 & 0 & 0 \\ 0 & Q_{2,2} & 0 & 0 & 0 \\ 0 & 0 & Q_{3,3} & 0 & 0 \\ 0 & 0 & 0 & Q_{4,4} & 0 \\ 0 & 0 & 0 & 0 & Q_{5,5} \end{bmatrix}. \quad (29)$$

- The Jacobian ,

$$\textbf{Jacobian}(\mathbf{f}) = \begin{bmatrix} \frac{\partial f_1}{\partial x_1} & \frac{\partial f_1}{\partial x_2} & \frac{\partial f_1}{\partial x_3} & \frac{\partial f_1}{\partial x_4} & \frac{\partial f_1}{\partial x_5} \\ \frac{\partial f_2}{\partial x_1} & \frac{\partial f_2}{\partial x_2} & \frac{\partial f_2}{\partial x_3} & \frac{\partial f_2}{\partial x_4} & \frac{\partial f_2}{\partial x_5} \\ \frac{\partial f_3}{\partial x_1} & \frac{\partial f_3}{\partial x_2} & \frac{\partial f_3}{\partial x_3} & \frac{\partial f_3}{\partial x_4} & \frac{\partial f_3}{\partial x_5} \\ \frac{\partial f_4}{\partial x_1} & \frac{\partial f_4}{\partial x_2} & \frac{\partial f_4}{\partial x_3} & \frac{\partial f_4}{\partial x_4} & \frac{\partial f_4}{\partial x_5} \\ \frac{\partial f_5}{\partial x_1} & \frac{\partial f_5}{\partial x_2} & \frac{\partial f_5}{\partial x_3} & \frac{\partial f_5}{\partial x_4} & \frac{\partial f_5}{\partial x_5} \end{bmatrix} \quad (30)$$

Given these conditions and the Equation 20, we have the following MRDE to compute the predicted state error covariance $P(t_{k/k-1})$ from t_{k-1} to t_k,

$$\frac{d\mathbf{P}(t)}{dt} = \begin{bmatrix} Q_{1,1} + 2.\frac{\partial f_1}{\partial x_1}.P_{1,1} & P_{1,1}.\frac{\partial f_2}{\partial x_1} + \frac{\partial f_1}{\partial x_2}.P_{2,2} & P_{1,1}.\frac{\partial f_3}{\partial x_1} + \frac{\partial f_1}{\partial x_3}.P_{3,3} & P_{1,1}.\frac{\partial f_4}{\partial x_1} + \frac{\partial f_1}{\partial x_4}.P_{4,4} & P_{1,1}.\frac{\partial f_5}{\partial x_1} + \frac{\partial f_1}{\partial x_5}.P_{5,5} \\ P_{2,2}.\frac{\partial f_1}{\partial x_2} + \frac{\partial f_2}{\partial x_1}.P_{1,1} & Q_{2,2} + 2.\frac{\partial f_2}{\partial x_2}.P_{2,2} & P_{2,2}.\frac{\partial f_3}{\partial x_2} + \frac{\partial f_2}{\partial x_3}.P_{3,3} & P_{2,2}.\frac{\partial f_4}{\partial x_2} + \frac{\partial f_2}{\partial x_4}.P_{4,4} & P_{2,2}.\frac{\partial f_5}{\partial x_2} + \frac{\partial f_2}{\partial x_5}.P_{5,5} \\ P_{3,3}.\frac{\partial f_1}{\partial x_3} + \frac{\partial f_3}{\partial x_1}.P_{1,1} & P_{3,3}.\frac{\partial f_2}{\partial x_3} + \frac{\partial f_3}{\partial x_2}.P_{2,2} & Q_{3,3} + 2.\frac{\partial f_3}{\partial x_3}.P_{3,3} & P_{3,3}.\frac{\partial f_4}{\partial x_3} + \frac{\partial f_3}{\partial x_4}.P_{4,4} & P_{3,3}.\frac{\partial f_5}{\partial x_3} + \frac{\partial f_3}{\partial x_5}.P_{5,5} \\ P_{4,4}.\frac{\partial f_1}{\partial x_4} + \frac{\partial f_4}{\partial x_1}.P_{1,1} & P_{4,4}.\frac{\partial f_2}{\partial x_4} + \frac{\partial f_4}{\partial x_2}.P_{2,2} & P_{4,4}.\frac{\partial f_3}{\partial x_4} + \frac{\partial f_4}{\partial x_3}.P_{3,3} & Q_{4,4} + 2.\frac{\partial f_4}{\partial x_4}.P_{4,4} & P_{4,4}.\frac{\partial f_5}{\partial x_4} + \frac{\partial f_4}{\partial x_5}.P_{5,5} \\ P_{5,5}.\frac{\partial f_1}{\partial x_5} + \frac{\partial f_5}{\partial x_1}.P_{1,1} & P_{5,5}.\frac{\partial f_2}{\partial x_5} + \frac{\partial f_5}{\partial x_2}.P_{2,2} & P_{5,5}.\frac{\partial f_3}{\partial x_5} + \frac{\partial f_5}{\partial x_3}.P_{3,3} & P_{5,5}.\frac{\partial f_4}{\partial x_5} + \frac{\partial f_5}{\partial x_4}.P_{4,4} & Q_{5,5} + 2.\frac{\partial f_5}{\partial x_5}.P_{5,5} \end{bmatrix}. \quad (31)$$

Then, we have that each ODE of the $\mathbf{P}(t_{k/k-1})$ is composed only of the elements of $P_{i,i}$ and $Q_{i,i}$ and elements of $\textbf{Jacobian}(\mathbf{f})$. Furthermore, these ODEs can be time invariant, if the partial derivative are zero. See the equation 28.

3.5. Only one variable measured

In the UMM case 1.2. For example, let consider the following state variable vector,

$$\psi_{case2} = [X_v, fgr, X_d, GLC, GLN, LAC, ASN, ASP, ALA, AMM, Mab, \mu_{max}, k_{31}, k_d] \quad (32)$$

To estimate the entire state variable vector is need to have the minimum of two measurement, and an option is Xv and GLC. Since, the column regards X_v in MRDE is zero k_{31} ODE, but it is different of zero in GLC column.

4. Example of the Lemma: Inability to Update Kalman Gain for Unshared parameters

Lemma 1. *The Kalman gain cannot be updated (by Eq 21) for an unshared parameter that is part of a state variable vector and part of a weak term in an UMM, if the initial state error covariance matrix* **P***(t=0) and* **Q** *are formed by uncorrelated elements and there is only one state variable measured.*

To illustrate this Lemma, we show that the Kalman gain value cannot be updated for the unshared parameter μ_{mp} of the UMM with weak terms presented in Case 1.1. Let's consider the following:

- The state variables vector

$$\psi(t)_{case1} = [Xv, N, MP, \mu_{Xv}, \mu_N, \mu_{mp}]. \quad (33)$$

- Xv is the unique measured variable and **H** = [1 0 0 0 0 0].
- R as measurement noise variance of Xv.
- μ_{mp} the unshared parameter to be evolved (estimated) and that is related to a weak term.
- **P** and **Q** with uncorrelated elements for the $\psi(t)_{case1}$ (Equation 33),

$$\mathbf{P} = \begin{bmatrix} P_{1,1} & 0 & 0 & 0 & 0 & 0 \\ 0 & P_{2,2} & 0 & 0 & 0 & 0 \\ 0 & 0 & P_{3,3} & 0 & 0 & 0 \\ 0 & 0 & 0 & P_{4,4} & 0 & 0 \\ 0 & 0 & 0 & 0 & P_{5,5} & 0 \\ 0 & 0 & 0 & 0 & 0 & P_{6,6} \end{bmatrix}, \mathbf{Q} = \begin{bmatrix} Q_{1,1} & 0 & 0 & 0 & 0 & 0 \\ 0 & Q_{2,2} & 0 & 0 & 0 & 0 \\ 0 & 0 & Q_{3,3} & 0 & 0 & 0 \\ 0 & 0 & 0 & Q_{4,4} & 0 & 0 \\ 0 & 0 & 0 & 0 & Q_{5,5} & 0 \\ 0 & 0 & 0 & 0 & 0 & Q_{6,6} \end{bmatrix}. \tag{34}$$

- The Jacobian $\mathbf{J}_t^{\phi}$, (Equation 12) with the $\psi(t)_{case1}$ (Equation 33),

$$\mathbf{J}_t^{\phi} = \begin{bmatrix} \mu_{Xv} & 0 & 0 & Xv & 0 & 0 \\ -\mu_N & 0 & 0 & 0 & -Xv & 0 \\ \mu_{mp} & 0 & 0 & 0 & 0 & Xv \\ 0 & 0 & 0 & 0 & 0 & 0 \\ 0 & 0 & 0 & 0 & 0 & 0 \\ 0 & 0 & 0 & 0 & 0 & 0 \end{bmatrix}. \tag{35}$$

Given these conditions and the Equation 20, we have the following MRDE (based on **P** with uncorrelated elements)

$$\frac{d\mathbf{P}(t)}{dt} = \begin{bmatrix} Q_{1,1} + 2.P_{1,1}.\mu_{Xv} & -P_{1,1}.\mu_N & P_{1,1}.\mu_{mp} & P_{4,4}.Xv & 0 & 0 \\ -P_{1,1}.\mu_N & Q_{2,2} & 0 & 0 & -P_{5,5}.Xv & 0 \\ P_{1,1}.\mu_{mp} & 0 & Q_{3,3} & 0 & 0 & P_{6,6}.Xv \\ P_{4,4}.Xv & 0 & 0 & Q_{4,4} & 0 & 0 \\ 0 & -P_{5,5}.Xv & 0 & 0 & Q_{5,5} & 0 \\ 0 & 0 & P_{6,6}.Xv & 0 & 0 & Q_{6,6} \end{bmatrix} = \tag{36}$$

$$\frac{d\mathbf{P}(t)}{dt} = \begin{bmatrix} \frac{P_{Xv,Xv}(t)}{dt} = Q_{1,1} + 2.P_{1,1}.\mu_{Xv} & \frac{P_{N,Xv}(t)}{dt} = -P_{1,1}.\mu_N & \ldots & \frac{P_{\mu_N,Xv}(t)}{dt} = 0 & \frac{P_{\mu mp,Xv}(t)}{dt} = 0 \\ \frac{P_{Xv,N}(t)}{dt} = -P_{1,1}.\mu_N & \frac{P_{N,N}(t)}{dt} = Q_{2,2} & \ldots & \frac{P_{\mu_N,N}(t)}{dt} = -P_{5,5}.Xv & \frac{P_{\mu mp,N}(t)}{dt} = 0 \\ \frac{P_{Xv,MP}(t)}{dt} = P_{1,1}.\mu_{mp} & \frac{P_{N,MP}(t)}{dt} = 0 & \ldots & \frac{P_{\mu_N,MP}(t)}{dt} = 0 & \frac{P_{\mu mp,MP}(t)}{dt} = P_{6,6}.Xv \\ \frac{P_{Xv,\mu_{Xv}}(t)}{dt} = P_{4,4}.Xv & \frac{P_{N,\mu_{Xv}}(t)}{dt} = 0 & \ldots & \frac{P_{\mu_N,\mu_{Xv}}(t)}{dt} = 0 & \frac{P_{\mu mp,\mu_{Xv}}(t)}{dt} = 0 \\ \frac{P_{Xv,\mu_N}(t)}{dt} = 0 & \frac{P_{N,\mu_N}(t)}{dt} = -P_{5,5}.Xv & \ldots & \frac{P_{\mu_N,\mu_N}(t)}{dt} = Q_{5,5} & \frac{P_{\mu mp,\mu_N}(t)}{dt} = 0 \\ \frac{P_{Xv,\mu mp}(t)}{dt} = 0 & \frac{P_{N,\mu mp}(t)}{dt} = 0 & \ldots & \frac{P_{\mu_N,\mu mp}(t)}{dt} = 0 & \frac{P_{\mu mp,\mu mp}(t)}{dt} = Q_{6,6} \end{bmatrix}. \tag{37}$$

Now, using this Equation 37 to compute the predicted state error covariance matrix $\mathbf{P}(t_{k/k-1})$ (for the Case 1.1) from t_{k-1} to t_k with a initial predicted state error covariance matrix $\mathbf{P}(t_{k-1}) = \mathbf{P}(t=0)$ with uncorrelated elements as following

$$\mathbf{P}(t=0) = \begin{bmatrix} P_{X_v,X_v}(t=0) & 0 & 0 & 0 & 0 & 0 \\ 0 & P_{N,N}(t=0) & 0 & 0 & 0 & 0 \\ 0 & 0 & P_{MP,MP}(t=0) & 0 & 0 & 0 \\ 0 & 0 & 0 & P_{\mu_{X_v},\mu_{X_v}}(t=0) & 0 & 0 \\ 0 & 0 & 0 & 0 & P_{\mu_N,\mu_N}(t=0) & 0 \\ 0 & 0 & 0 & 0 & 0 & P_{\mu_{mp},\mu_{mp}}(t=0) \end{bmatrix}, \tag{38}$$

we have

$$\mathbf{P}(t_{k/k-1}) = \begin{bmatrix} P_{X_v,X_v}(t_{k/k-1}) & P_{N,X_v}(t_{k/k-1}) & P_{MP,X_v}(t_{k/k-1}) & P_{\mu_{X_v},X_v}(t_{k/k-1}) & P_{\mu_N,X_v}(t_{k/k-1}) & P_{\mu_{mp},X_v}(t_{k/k-1}) \\ P_{X_v,N}(t_{k/k-1}) & P_{N,N}(t_{k/k-1}) & P_{MP,N}(t_{k/k-1}) & P_{\mu_{X_v},N}(t_{k/k-1}) & P_{\mu_N,N}(t_{k/k-1}) & P_{\mu_{mp},N}(t_{k/k-1}) \\ P_{X_v,MP}(t_{k/k-1}) & P_{N,MP}(t_{k/k-1}) & P_{MP,MP}(t_{k/k-1}) & P_{\mu_{X_v},MP}(t_{k/k-1}) & P_{\mu_N,MP}(t_{k/k-1}) & P_{\mu_{mp},MP}(t_{k/k-1}) \\ P_{X_v,\mu_{X_v}}(t_{k/k-1}) & P_{N,\mu_{X_v}}(t_{k/k-1}) & P_{MP,\mu_{X_v}}(t_{k/k-1}) & P_{\mu_{X_v},\mu_{X_v}}(t_{k/k-1}) & P_{\mu_N,\mu_{X_v}}(t_{k/k-1}) & P_{\mu_{mp},\mu_{X_v}}(t_{k/k-1}) \\ P_{X_v,\mu_N}(t_{k/k-1}) & P_{N,\mu_N}(t_{k/k-1}) & P_{MP,\mu_N}(t_{k/k-1}) & P_{\mu_{X_v},\mu_N}(t_{k/k-1}) & P_{\mu_N,\mu_N}(t_{k/k-1}) & P_{\mu_{mp},\mu_N}(t_{k/k-1}) \\ P_{X_v,\mu_{mp}}(t_{k/k-1}) & P_{N,\mu_{mp}}(t_{k/k-1}) & P_{MP,\mu_{mp}}(t_{k/k-1}) & P_{\mu_{X_v},\mu_{mp}}(t_{k/k-1}) & P_{\mu_N,\mu_{mp}}(t_{k/k-1}) & P_{\mu_{mp},\mu_{mp}}(t_{k/k-1}) \end{bmatrix} = \tag{39}$$

$$\mathbf{P}(t_{k/k-1}) = \begin{bmatrix} Cov(X_v,X_v) & Cov(N,X_v) & Cov(MP,X_v) & Cov(\mu_{X_v},X_v) & Cov(\mu_N,X_v) & Cov(\mu_{mp},X_v) \\ Cov(X_v,N) & Cov(N,N) & Cov(MP,N) & Cov(\mu_{X_v},N) & Cov(\mu_N,N) & Cov(\mu_{mp},N) \\ Cov(X_v,MP) & Cov(N,MP) & Cov(MP,MP) & Cov(\mu_{X_v},MP) & Cov(\mu_N,MP) & Cov(\mu_{mp},MP) \\ Cov(X_v,\mu_{X_v}) & Cov(N,\mu_{X_v}) & Cov(MP,\mu_{X_v}) & Cov(\mu_{X_v},\mu_{X_v}) & Cov(\mu_N,\mu_{X_v}) & Cov(\mu_{mp},\mu_{X_v}) \\ Cov(X_v,\mu_N) & Cov(N,\mu_N) & Cov(MP,\mu_N) & Cov(\mu_{X_v},\mu_N) & Cov(\mu_N,\mu_N) & Cov(\mu_{mp},\mu_N) \\ Cov(X_v,\mu_{mp}) & Cov(N,\mu_{mp}) & Cov(MP,\mu_{mp}) & Cov(\mu_{X_v},\mu_{mp}) & Cov(\mu_N,\mu_{mp}) & Cov(\mu_{mp},\mu_{mp}) \end{bmatrix}. \tag{40}$$

Now, using $\mathbf{P}(t_{k/k-1})$, $\mathbf{H}$ and R to compute the Kalman gain values for all variables in the state variable vector, we have

$$\mathbf{K}_k = \mathbf{P}(t_{k|k-1})\mathbf{H}^T(\mathbf{H}\mathbf{P}(t_{k|k-1})\mathbf{H}^T + \mathbf{R})^{-1} = \begin{bmatrix} K_{X_v} \\ K_N \\ K_{MP} \\ K_{\mu_{X_v}} \\ K_{\mu_N} \\ K_{\mu_{mp}} \end{bmatrix} = \begin{bmatrix} \frac{P_{X_v,X_v}(t_{k/k-1})}{P_{X_v,X_v}(t_{k/k-1})+R} \\ \frac{P_{X_v,N}(t_{k/k-1})}{P_{X_v,X_v}(t_{k/k-1})+R} \\ \frac{P_{X_v,MP}(t_{k/k-1})}{P_{X_v,X_v}(t_{k/k-1})+R} \\ \frac{P_{X_v,\mu_{X_v}}(t_{k/k-1})}{P_{X_v,X_v}(t_{k/k-1})+R} \\ \frac{P_{X_v,\mu_N}(t_{k/k-1})}{P_{X_v,X_v}(t_{k/k-1})+R} \\ \frac{P_{X_v,\mu_{mp}}(t_{k/k-1})}{P_{X_v,X_v}(t_{k/k-1})+R} \end{bmatrix} = \begin{bmatrix} \frac{P_{X_v,X_v}(t_{k/k-1})}{P_{X_v,X_v}(t_{k/k-1})+R} \\ \frac{P_{X_v,N}(t_{k/k-1})}{P_{X_v,X_v}(t_{k/k-1})+R} \\ \frac{P_{X_v,MP}(t_{k/k-1})}{P_{X_v,X_v}(t_{k/k-1})+R} \\ \frac{P_{X_v,\mu_{X_v}}(t_{k/k-1})}{P_{X_v,X_v}(t_{k/k-1})+R} \\ \frac{P_{X_v,\mu_N}(t_{k/k-1})}{P_{X_v,X_v}(t_{k/k-1})+R} \\ \frac{0}{P_{X_v,X_v}(t_{k/k-1})+R} \end{bmatrix} = \begin{bmatrix} \frac{P_{X_v,X_v}(t_{k/k-1})}{P_{X_v,X_v}(t_{k/k-1})+R} \\ \frac{P_{X_v,N}(t_{k/k-1})}{P_{X_v,X_v}(t_{k/k-1})+R} \\ \frac{P_{X_v,MP}(t_{k/k-1})}{P_{X_v,X_v}(t_{k/k-1})+R} \\ \frac{P_{X_v,\mu_{X_v}}(t_{k/k-1})}{P_{X_v,X_v}(t_{k/k-1})+R} \\ \frac{P_{X_v,\mu_N}(t_{k/k-1})}{P_{X_v,X_v}(t_{k/k-1})+R} \\ 0 \end{bmatrix}. \tag{41}$$

$\mathbf{H}$ selected the first column of $\mathbf{P}(t_{k/k-1})$ since it is related to the measured value X_v. However, in this column, we have that the predicted error covariance between X_v and μ_{mp} is zero, $Cov(X_v, \mu_{mp}) = 0$. Since the solution of $\frac{P_{X_v,\mu_{mp}}(t)}{dt} = 0$ obtained from t_{k-1} to t_k is zero, and we have $P_{X_v,\mu_{mp}}(t_{k/k-1}) = P_{\mu_{X_v},\mu_{mp}}(t_{k-1}) = P_{\mu_{X_v},\mu_{mp}}(t = 0) = 0$. This means that due to $\mathbf{P}$(t=0) with uncorrelated elements the obtained solution is equal to the initial condition. Then, we have the kalman gain value for the unshared parameter is zero, $K_{\mu_{mp}} = 0$, and consequently the predicted state error covariance $P_{X_v,\mu_{mp}}(t_{k/k-1})$ cannot be updated in $\mathbf{P}(t_{k|k-1})$ by Eq 23. Since

$$\mathbf{P}(t_{k|k}) = (\mathbf{I} - \mathbf{K}_k\mathbf{H})\mathbf{P}(t_{k|k-1}) = \begin{bmatrix} P_{X_v,X_v}(t_{k/k-1}) - K_{Xv}.P_{X_v,X_v}(t_{k/k-1}) & ... \\ P_{X_v,N}(t_{k/k-1}) - K_N.P_{X_v,X_v}(t_{k/k-1}) & ... \\ P_{X_v,MP}(t_{k/k-1}) - K_{mp}.P_{X_v,X_v}(t_{k/k-1}) & ... \\ P_{X_v,\mu_{X_v}}(t_{k/k-1}) - K_{\mu_{Xv}}.P_{X_v,X_v}(t_{k/k-1}) & ... \\ P_{X_v,\mu_N}(t_{k/k-1}) - K_{\mu_N}.P_{X_v,X_v}(t_{k/k-1}) & ... \\ P_{X_v,\mu_{mp}}(t_{k/k-1}) - K_{\mu_{mp}}.P_{X_v,X_v}(t_{k/k-1}) & ... \end{bmatrix} = \tag{42}$$

$$\mathbf{P}(t_{k|k}) = (\mathbf{I} - \mathbf{K}_k\mathbf{H})\mathbf{P}(t_{k|k-1}) = \begin{bmatrix} P_{X_v,X_v}(t_{k/k-1}) - K_{Xv}.P_{X_v,X_v}(t_{k/k-1}) & \dots \\ P_{X_v,N}(t_{k/k-1}) - K_N.P_{X_v,X_v}(t_{k/k-1}) & \dots \\ P_{X_v,MP}(t_{k/k-1}) - K_{mp}.P_{X_v,X_v}(t_{k/k-1}) & \dots \\ P_{X_v,\mu_{Xv}}(t_{k/k-1}) - K_{\mu_{Xv}}.P_{X_v,X_v}(t_{k/k-1}) & \dots \\ P_{X_v,\mu_N}(t_{k/k-1}) - K_{\mu_N}.P_{X_v,X_v}(t_{k/k-1}) & \dots \\ 0 - 0.P_{X_v,X_v}(t_{k/k-1}) & \dots \end{bmatrix}. \quad (43)$$

We have that $P_{X_v,\mu_{mp}}(t_{k/k}) = P_{X_v,\mu_{mp}}(t_{k/k-1}) = 0$, and as the $P_{X_v,\mu_{mp}}(t_{k/k}) = 0$ have to be used as a new initial condition for MRDE (Equation 37), we have $K_{\mu_{mp}} = 0$ for all $P_{X_v,\mu_{mp}}(t_{k/k-1})$ in $\mathbf{P}(t_{k/k-1})$ that are obtained from t_{k-1} to t_k using Equation 37 and consequently no updates for $K_{\mu_{mp}}$ and $P_{X_v,\mu_{mp}}(t_{k/k-1})$.

5. Example of the Theorem (JEKF failure)

Theorem 1. *The JEKF fails to estimate an unshared parameter (parameter evolution) that is part of a state variable vector and part of a weak term in a UMM if the initial state error covariance matrix* $\mathbf{P}$*(t=0) and* $\mathbf{Q}$ *are composed of uncorrelated elements, and there is only one state variable measured. This is because the Kalman gain value for the unshared parameter is equal to zero for all steps of execution of the JEKF algorithm.*

To illustrate this Theorem, we show the JEKF failure with the condition and results used in the example 4 of the Lemma 1.

Then, let's consider the following:

- the UMM of Case 1.1.
- $\mathbf{H}$=[1 0 0 0 0 0] and $\mathbf{K}_k = [\mathbf{K}_{X_v}, \mathbf{K}_N, \mathbf{K}_{MP}, \mathbf{K}_{\mu_{X_v}}, \mathbf{K}_{\mu_N}, \mathbf{K}_{\mu_{mp}}]^T$ as obtained in the proof of Lemma 1, where $\mathbf{K}_{\mu_{mp}} = 0$.
- $\mathbf{Z}_k$ as measured value of X_v .
- $\hat{\boldsymbol{\psi}}(t_{k/k-1})_{case1} = [\hat{X}_v, \hat{N}, \hat{MP}, \hat{\mu_{Xv}}, \hat{\mu_N}, \hat{\mu_{mp}}]^T$.

Now, using the Equation 22 to provide the estimated mean of the state variable vector $\hat{\boldsymbol{\psi}}(t_{k/k})_{case1}$, we have

$$\hat{\boldsymbol{\psi}}(t_{k/k})_{case1} = \hat{\boldsymbol{\psi}}(t_{k/k-1})_{case1} + \mathbf{K}_k(\mathbf{Z}_k - \mathbf{H}\hat{\boldsymbol{\psi}}(t_{k/k-1})_{case1}) \quad (44)$$

$$\hat{\boldsymbol{\psi}}(t_{k/k})_{case1} = \begin{bmatrix} \hat{X}_v \\ \hat{N} \\ \hat{MP} \\ \hat{\mu_{Xv}} \\ \hat{\mu_N} \\ \hat{\mu_{mp}} \end{bmatrix} + \begin{bmatrix} \mathbf{K}_{X_v} \\ \mathbf{K}_N \\ \mathbf{K}_{MP} \\ \mathbf{K}_{\mu_{X_v}} \\ \mathbf{K}_{\mu_N} \\ \mathbf{K}_{\mu_{mp}} \end{bmatrix} .(\mathbf{Z}_k - X_v) = \begin{bmatrix} \hat{X}_v + \mathbf{K}_{X_v}.(\mathbf{Z}_k - \hat{X}_v) \\ \hat{N} + \mathbf{K}_N.(\mathbf{Z}_k - \hat{X}_v) \\ \hat{MP} + \mathbf{K}_{MP}.(\mathbf{Z}_k - \hat{X}_v) \\ \hat{\mu_{Xv}} + \mathbf{K}_{\mu_{X_v}}.(\mathbf{Z}_k - \hat{X}_v) \\ \hat{\mu_N} + \mathbf{K}_{\mu_{mp}}.(\mathbf{Z}_k - \hat{X}_v) \\ \hat{\mu_{mp}} + 0 \end{bmatrix} \quad (45)$$

Then, we have the estimated mean $\hat{\boldsymbol{\psi}}(t_{k/k})_{case1}$ of the unshared parameter is equal to the predicted mean $\hat{\boldsymbol{\psi}}(t_{k/k-1})_{case1}$ of unshared parameter for all step from t_{k-1} to t_k. In another words, the JEKF failure to perform the parameter evolution, since $\hat{\mu_{mp}}(t_{k/k}) = \hat{\mu_{mp}}(t_{k/k-1})$ all step from t_{k-1} to t_k.

6. Related work: Approach KPH2

In this section, we describe the approach KPH2 [6] that can to side-step JEFK failure. The authors did not give details about the approach, because the focus was to report application developed to monitoring a rAAV production that is a new bioprocess. Basically, the KPH2 tries to prevent the Kalman gain value regards to an unshared parameter from being zero. Because Kalman gain value equal to zero resulted from an low process uncertainty would mean that the prediction regarding the unshared parameter is perfect

and does not need the influence of the measurement in the correction step of JEKF since there is no uncertainty in the prediction regarding the unshared parameter. This is an unrealistic situation and therefore, there is the need to increase the Process uncertainty, $\mathbf{K}_k \approx \frac{Process\ _{Uncertainty}}{System\ _{Uncertainty}}$ to obtain $|K| > 0$ and enable the predicted unshared parameter to be corrected by the influence of residual error in JEKF algorithm. The KPH2 approach tries to fulfill this need by adding more information about the prior error covariances regard to an unshared parameter in two update steps: i) in the Kalman gain computation (Equation 21) and ii) in the update of predicted state error covariance matrix (Equation 23) of EKF algorithm. In the following, we describe the details of this.

The Process uncertainty (Equation 24) is only composed of prior error covariance related to the measured state variable and this information is "incomplete" with regards to unshared parameter, $Cov(MSV, UP) = 0$ in the initial condition **P**(t=0) and in the predictions during the process of execution of JEKF algorithm. However, the prior error covariances related to unshared parameter $\mathbf{P}_{i,UP}(t_{k|k-1})$ are informations that are already available in the $\mathbf{P}(t_{k|k-1})$ and can be easily extracted from it. Then, an approach to increase process uncertainty (in Equation 24) is to add the Prior error covariances of unshared parameter $\mathbf{P}_{i,UP}(t_{k|k-1})$ to $\mathbf{P}(t_{k|k-1})\mathbf{H}^T$ in Equation 21 as following. Given that $\mathbf{P}(t_{k|k-1})\mathbf{H}^T$ is a vector with prior error covariances of each state variable (SV) with measured state variable (MSV)

$$\mathbf{P}(t_{k|k-1})\mathbf{H}^T = \begin{bmatrix} Cov(SV_i, MSV) \\ \vdots \\ Cov(SV_n, MSV) \end{bmatrix}, \tag{46}$$

and $\mathbf{P}_{i,UP}(t_{k|k-1})$ is also a vector with prior error covariances of each state variable (SV) with all unshared parameter to be estimated

$$\mathbf{P}_{i,UP}(t_{k|k-1}) = \begin{bmatrix} Cov(SV_i, \sum_i^j UP_i) \\ \vdots \\ Cov(SV_n, \sum_i^j UP_i) \end{bmatrix}, \tag{47}$$

we have that the sum of $\mathbf{P}_{i,UP}(t_{k|k-1})$ and $\mathbf{P}(t_{k|k-1})\mathbf{H}^T$ in the equation 21 is

$$\mathbf{K}_k = (\mathbf{P}(t_{k|k-1})\mathbf{H}^T + \mathbf{P}_{i,UP}(t_{k|k-1}))(\mathbf{H}\mathbf{P}(t_{k|k-1})\mathbf{H}^T + \mathbf{R})^{-1}. \tag{48}$$

Since, all information that we need to perform $\mathbf{P}(t_{k|k-1})\mathbf{H}^T + \mathbf{P}_{i,UP}(t_{k|k-1})$ are available in $\mathbf{P}(t_{k|k-1})$, we can apply a specific linear operator $\mathbf{H}_2$ (row vector) to $\mathbf{P}(t_{k|k-1})$ to extract all information easily. We need only to defining $\mathbf{H}_2$ with the state variable vector "position" of MSV and UP. For example, if the position of MSV and UP in the state variable vector, $\psi(t) = [MSV, x_2, x_3, x_4, UP]$, is 1st and 5th, we have $\mathbf{H}_2$ =[1 0 0 0 1]. Then, the final version of the Equation 48 is

$$\mathbf{K}_k = \mathbf{P}(t_{k|k-1})\mathbf{H}_2^T(\mathbf{H}\mathbf{P}(t_{k|k-1})\mathbf{H}^T + \mathbf{R})^{-1}. \tag{49}$$

In theory, this Equation 49 could prevent $\mathbf{K} = 0$. However, in the case of too many unshared parameters to be estimated, it can unbalance the ratio in the Equation 24, since the Process uncertainty (PU) can become too large in relation to the System uncertainty (SU) in the entire process of execution of JEKF algorithm. Then, to try to preserve a more realistic ratio between PU and SU, the PU that compose the SU can be increased. Since $SU = PU + Measurement_{uncertainty} = \mathbf{H}\mathbf{P}(t_{k|k-1})\mathbf{H}^T + \mathbf{R}$ [22]. Given this, an approach is add the total sum of all prior error covariances of unshared parameters to be estimated $\mathbf{P}_{UP_{total}}(t_{k|k-1})$ to $\mathbf{H}\mathbf{P}(t_{k|k-1})\mathbf{H}^T$ that is part of the system uncertainty (Equation 49) as following. Given that $\mathbf{H}\mathbf{P}(t_{k|k-1})\mathbf{H}^T$ results in a scalar representing prior error variance of MSV

$$\mathbf{HP}(t_{k|k-1})\mathbf{H}^T = Cov(MSV, MSV), \tag{50}$$

and $\mathbf{P}_{UP_{total}}(t_{k|k-1})$ is also a scalar, but representing the total sum of all prior error covariances related with the unshared parameters to be estimated

$$\mathbf{P}_{UP_{total}}(t_{k|k-1}) = Cov(MSV, \sum_i^n UP_i) + \sum_j^n Cov(UP_j, MSV + \sum_i^n UP_i), \tag{51}$$

we have that the sum of $\mathbf{P}_{UP_{total}}(t_{k|k-1})$ and $\mathbf{HP}(t_{k|k-1})\mathbf{H}^T$ in the equation 49 is

$$\mathbf{K}_k = (\mathbf{P}(t_{k|k-1})\mathbf{H}^T + \mathbf{P}_{i,UP}(t_{k|k-1}))(\mathbf{HP}(t_{k|k-1})\mathbf{H}^T + \mathbf{P}_{UP_{total}}(t_{k|k-1}) + \mathbf{R})^{-1}. \tag{52}$$

Since all information that we need to perform the sum of $\mathbf{P}_{UP_{total}}(t_{k|k-1})$ and $\mathbf{HP}(t_{k|k-1})\mathbf{H}^T$ are available in $\mathbf{P}(t_{k|k-1})$. We can apply a specific linear operator $\mathbf{H}_2$ to $\mathbf{P}(t_{k|k-1})$ to extract all information easily. Then, the final version of the Equation 52 is

$$\mathbf{K}_k = \mathbf{P}(t_{k|k-1})\mathbf{H}_2^T(\mathbf{H}_2\mathbf{P}(t_{k|k-1})\mathbf{H}_2^T + \mathbf{R})^{-1}. \tag{53}$$

The Equation 53 tries to preserve a more realistic ratio between PU and SU by increasing the PU that compose the SU. However, if the kalman gain continue being small, the $\mathbf{P}(t_{k|k-1})$ could be updated slowly by the Equation 23. Because $\mathbf{K}_k\mathbf{HP}(t_{k|k-1})$ is the factor that update $\mathbf{P}(t_{k|k-1})$ and it is totally dependent of $\mathbf{K}$, as we can see in

$$\mathbf{K}_k\mathbf{HP}(t_{k|k-1}) = \begin{bmatrix} Cov(MSV, SV_1).K_1, \ldots, Cov(MSV, SV_n).K_1, \\ \vdots \\ Cov(MSV, SV_1).K_n, \ldots, Cov(MSV, SV_n).K_n, \end{bmatrix}. \tag{54}$$

Them, an artefact to avoid a possible slow update of $\mathbf{P}(t_{k|k-1})$ can be to add the Prior error covariance related to unshared parameter $\mathbf{P}_{i,UP}(t_{k|k-1})$ to the $\mathbf{K}_k\mathbf{HP}(t_{k|k-1})$ in the Equation 23 as the following way

$$\mathbf{K}_k\mathbf{H}_2\mathbf{P}(t_{k|k-1}) = \begin{bmatrix} (Cov(MSV, SV_1) + Cov(\sum_i^j UP_i, SV_1)).K_1, \ldots, (Cov(MSV, SV_n) + Cov(\sum_i^j UP_i, SV_n)).K_1, \\ \vdots \\ (Cov(MSV, SV_1) + Cov(\sum_i^j UP_i, SV_1)).K_n, \ldots, (Cov(MSV, SV_n) + Cov(\sum_i^j UP_i, SV_n)).K_n, \end{bmatrix} \tag{55}$$

$$\mathbf{P}(t_{k|k}) = (\mathbf{I} - \mathbf{K}_k\mathbf{H}_2)\mathbf{P}(t_{k|k-1}), \tag{56}$$

where $\mathbf{H}_2$ is the same linear operator used in Equation 53 .

7. Empirical Evaluation - Extension

7.1. Synthetic dataset development - mAb production

The Synthetic dataset (SD) is composed of two runs (A-SD, and B-SD). The runs have different samples regarding the state variables Xv, GLC, GLN, LAC, AMM, and mAb and were generated using the UMM case 1.4 with three set of different parameters. These parameters are presented in the Table S1.

Table S1. Parameters used in UMM case 1.4 to generate the runs A-SD, and B-SD of Synthetic Dataset (SD).

Parameter	Name	run A-SD	run B-SD
$\mu_{max}(h^{-})$	Maximum growth rate	5.8×10^{-2}	7.5×10^{-2}
$k_{glc}(mM)$	Monod constant glucose	7.5×10^{-1}	7.5×10^{-1}
$k_{gln}(mM)$	Monod constant glutamine	7.5×10^{-2}	7.5×10^{-2}
$k_{Ilac}(mM)$	Monod constant lactate for inhibition	1.72×10^{2}	1.72×10^{2}
$k_{Iamm}(mM)$	Monod constant ammonium for inhibition	2.85×10^{1}	2.85×10^{1}
$\mu_{d,max}(h^{-})$	Maximum death rate	3.0×10^{-2}	3.0×10^{-2}
$K_{d,amm}(mM)$	Monod constant ammonium for death	1.76	1.76
$K_{lysis}(h^{-})$	Breakdown of cell membranes	5.51×10^{-2}	5.51×10^{-2}
$Y_{X,glc}(cells\ mmol^{-})$	Yield coefficient cell conc./glucose	1.06×10^{8}	1.06×10^{8}
$m_{glc}(mmol/cells\ h)$	Glucose maintenance coefficient	4.85×10^{-14}	4.85×10^{-14}
$Y_{X,gln}(cells/mmmol)$	Yield coefficient cell conc./glutamine	5.57×10^{8}	5.57×10^{8}
$\alpha_1(mmols\ cells^{-}\ h^{-})$	Coefficient for m_{gln}	3.40×10^{-13}	3.40×10^{-13}
$\alpha_2(mM)$	Coefficient for m_{gln}	4.0	4.0
$k_{d,gln}(h^{-})$	Monod constant glutamine for death	9.6×10^{-3}	9.6×10^{-3}
$Y_{lac/glc}(1)$	Yield coefficient lactate/glucose	1.4	1.4
$Y_{amm/gln}(1)$	Yield coefficient ammonium/glutamine	4.27×10^{-1}	4.27×10^{-1}
γ	constant parameter	4.27×10^{-1}	4.27×10^{-1}
$Q_{mAb}(mg\ cells^{-}\ h^{-})$	mAb specific production rate	7.21×10^{-9}	9.21×10^{-9}

Table S2. Initial conditions of state variables of UMM case 1.4 for the JEKF test with Synthetic Dataset.

State Variable	Name	Value
Xv	Viable cells density	2×10^{8} c/mL
Xt	total cells density	2×10^{8} c/mL
GLC	Glucose	29.1 mM
GLN	Glutamine	4.9 mM
LAC	Lactate	0 mM
AMM	Ammonium	0.31 mM
mAb	Monoclonal Antibody (titer)	80.6 mg/L
QmAb	Specific production rate of mAb	$7.21\times10^{-9}\ mg\ cells^{-1}h^{-1}$

7.2. Real dataset development - rAAV production

The details of Real dataset (RD) can be found in [6].

7.3. NSEs (JEKF-classic, JEKF-SANTO and JEKF-KPH2) design to address RQ1-G1 and RQ2-G2

The process model (based on UMM case 1.4) and joint state variable vector used by JEKF-Classic, JEKF-SANTO and JEKF-KPH2 are the following:

$$\boldsymbol{\psi}(t)_{case4} = [X_V, X_t, GLC, GLN, LAC, AMM, mAb, QmAb]^T, \tag{57}$$

and

$$\frac{d}{dt}\begin{bmatrix} X_V \\ X_t \\ GLC \\ GLN \\ LAC \\ AMM \\ mAb \\ QmAb \end{bmatrix} = \begin{bmatrix} f_{X_V} \\ f_{GLC} \\ f_{GLN} \\ f_{LAC} \\ f_{AMM} \\ f_{mAb} \\ 0 \end{bmatrix} + \boldsymbol{\omega}(t). \tag{58}$$

The standard and specific **P**(t=0) that were used with run B of Synthetic Dataset to address the RQ1-G1 and RQ2-G2 are in Tables S3, S4, and S5.

In regards to the run B-SD, the standard **P**(t=0) for the NSEs were obtained following $\mathbf{P}(t=0) = diag((\boldsymbol{\psi}_{case4}(t=0) - \hat{\boldsymbol{\psi}}_{case4}(t=0))(\boldsymbol{\psi}_{case4}(t=0) - \hat{\boldsymbol{\psi}}_{case4}(t=0))^T)$ as done in [4,6]. Then, we have $\mathrm{P_{QmAb,QmAb}} = (9.21 \times 10^{-9} - 07.21 \times 10^{-9})^2 = 3.9 \times 10^{-18}(g\ cells^{-1}h^{-1})^2$, see Table S3. Since $\mathrm{P_{X_v,QmAb}}$ is a off-diagonal element, we defined it as 1/4 of $\mathrm{P_{QmAb,QmAb}}$ $(\mathrm{g\ cells^{-1}h^{-1}})^2$. Then, $\mathrm{P_{X_v,QmAb}} = (3.9 \times 10^{-18})/4 = 9.99 \times 10^{-19}$ $(c^2/mL^2)(g\ cells^{-1}h^{-1})$. On the other hand, the specific **P**(t=0) for the NSEs were obtained by trial and error. Furthermore, the **R** and **Q** used by the NSEs (for runs B of Synthetic Dataset) are presented in Tables S6 and S7. It is important point out that all NSEs used a standard and specific **Q** that were obtained by by trial and error until achieve positive results in the Innovation Magnitude Bound Test and the Normalised Innovations Squared Chi-square Test.

Table S3. Standard initial state error covariance matrix (standard **P**(t=0)) for JEKF-Classic, JEKF-KPH2 and JEKF-SANTO with run B of Synthetic Dataset.

Parameter	Name	$\mathbf{P}_{i,i}$ for JEKF-Classic and JEKF-KPH2 in MRDE-PC and MRDE-PU	$\mathbf{P}_{i,i}$ for JEKF-SANTO in MRDE-PC and MRDE-PU
P_{X_v,X_v} (c^2/mL^2)	Viable cells	0.00	0.00
$P_{GLC,GLC}$ (mM^2)	Glucose	0.00	0.00
$P_{GLN,GLN}$ (mM^2)	Glutamine	0.00	0.00
$P_{LAC,LAC}$ (mM^2)	Lactate	0.00	0.00
$P_{AMM,AMM}$ (mM^2)	Ammonium	0.00	0.00
$P_{mAb,mAb}$ $(mg/L)^2$	Monoclonal Antibody (titer)	0.00	0.00
$P_{QmAb,QmAb}$ $(g\ cells^{-1}h^{-1})^2$	Specific production rate of mAb	3.9e-18	3.9e-18
$P_{X_v,QmAb}$ $(c^2/mL^2)(g\ cells^{-1}h^{-1})$	Initial $Cov(X_v, QmAb)$	0.0	9.99e-19

Table S4. Specific initial state error covariance matrix (specific **P**(t=0)) for JEKF-KPH2 with run B of Synthetic Dataset.

Parameter	Name	Value in MRDE-PC	Value in MRDE-PU
P_{X_v,X_v} (c^2/mL^2)	Viable cells	0.00	0.00
$P_{GLC,GLC}$ (mM^2)	Glucose	0.00	0.00
$P_{GLN,GLN}$ (mM^2)	Glutamine	0.00	0.00
$P_{LAC,LAC}$ (mM^2)	Lactate	0.00	0.00
$P_{AMM,AMM}$ (mM^2)	Ammonium	0.00	0.00
$P_{mAb,mAb}$ $(mg/L)^2$	Monoclonal Antibody (titer)	0.00	0.00
$P_{QmAb,QmAb}$ $(g\ cells^{-1}h^{-1})^2$	Specific production rate of mAb	12.21e-1	11.97e-2

Table S5. Specific initial state error covariance matrix (specific **P**(t=0)) for JEKF-SANTO with run B of Synthetic Dataset.

Parameter	Name	Value in MRDE-PC	Value in MRDE-PU
P_{X_v,X_v} (c^2/mL^2)	$Cov(x_v, x_v)$	0.00	0.00
$P_{GLC,GLC}$ (mM^2)	Glucose	0.00	0.00
$P_{GLN,GLN}$ (mM^2)	Glutamine	0.00	0.00
$P_{LAC,LAC}$ (mM^2)	Lactate	0.00	0.00
$P_{AMM,AMM}$ (mM^2)	Ammonium	0.00	0.00
$P_{mAb,mAb}$ $(mg/L)^2$	Monoclonal Antibody (titer)	0.00	0.00
$P_{QmAb,QmAb}$ $(g\ cells^{-1}h^{-1})^2$	Specific production rate of mAb	3.9e-18	3.9e-18
$P_{X_V,QmAb}$ $(c^2/mL^2)(g\ cells^{-1}h^{-1})$	Initial $Cov(X_V, QmAb)$	0.754	0.1445

Table S6. Measurement noise variance **R** and error covariance matrix of process model (**Q**) for the JEKF-Classic, JEKF-SANTO and JEKF-KPH2 with run B of Synthetic Dataset using MRDE-PC.

		Standard **Q**	Specific **Q**	
**Parameter**	**Name**	**JEKF-Classic, JEKF-SANTO, and JEKF-KPH2 values with MRDE-PC and standard P(0)**	**JEKF-SANTO values with MRDE-PC and specific P(0)**	**JEKF-KPH2 values with MRDE-PC with specific P(0)**
R^2 (c^2/mL^2)	Viable cells MNV [1]	$(20\times 10^7)^2$	$(20\times 10^7)^2$	$(20\times 10^7)^2$
Q_{X_v,X_v} (c^2/mL^2)	Viable cells PNV [2]	$(90\times 10^6)^2$	$(90\times 10^6)^2$	$(90\times 10^6)^2$
Q_{X_t,X_t} (c^2/mL^2)	Viable cells PNV [2]	0.001	0.001	0.001
$Q_{GLC,GLC}$ (mM^2)	Glucose PNV	0.001	0.001	0.001
$Q_{GLN,GLN}$ mM^2	Glutamine PNV	0.001	0.001	0.001
$Q_{LAC,LAC}$ (mM^2)	Lactate PNV	0.001	0.001	0.001
$Q_{AMM,AMM}$ (mM^2)	Ammonium PNV	0.001	0.001	0.001
$Q_{mAb,mAb}$ (VG^2/mL^2)	Monoclonal Antibody (titer) PNV	0.001	1.7	0.001
$Q_{QmAb,QmAb}$ (h^{-2})	Specific production rate of mAb	$(1\times 10^9)^2$	1×10^9	$(1\times 10^8)^2$

[1] MNV—measurement noise value; [2] PNV—process noise value.

Table S7. Measurement noise variance **R** and error covariance matrix of process model (**Q**) for the JEKF-Classic, JEKF-SANTO and JEKF-KPH2 with run B of Synthetic Dataset using MRDE-PU.

		Standard **Q**	Specific **Q**	
**Parameter**	**Name**	**JEKF-Classic, JEKF-SANTO, and JEKF-KPH2 values with MRDE-PU and standard P(0)**	**JEKF-SANTO values with MRDE-PU and specific P(0)**	**JEKF-KPH2 values with MRDE-PU with specific P(0)**
R^2 (c^2/mL^2)	Viable cells MNV [1]	$(20\times 10^7)^2$	$(20\times 10^7)^2$	$(20\times 10^7)^2$
Q_{X_v,X_v} (c^2/mL^2)	Viable cells PNV [2]	$(20\times 10^6)^2$	$(20\times 10^6)^2$	$(20\times 10^6)^2$
Q_{X_t,X_t} (c^2/mL^2)	Viable cells PNV [2]	0.001	0.001	0.001
$Q_{GLC,GLC}$ (mM^2)	Glucose PNV	0.001	0.001	0.001
$Q_{GLN,GLN}$ mM^2	Glutamine PNV	0.001	0.001	0.001
$Q_{LAC,LAC}$ (mM^2)	Lactate PNV	0.001	0.001	0.001
$Q_{AMM,AMM}$ (mM^2)	Ammonium PNV	0.001	0.001	0.001
$Q_{mAb,mAb}$ (VG^2/mL^2)	Monoclonal Antibody (titer) PNV	1.04	1.7	20300.9
$Q_{QmAb,QmAb}$ (h^{-2})	Specific production rate of mAb	17×10^{-15}	1×10^9	0.001

[1] MNV—measurement noise value; [2] PNV—process noise value.

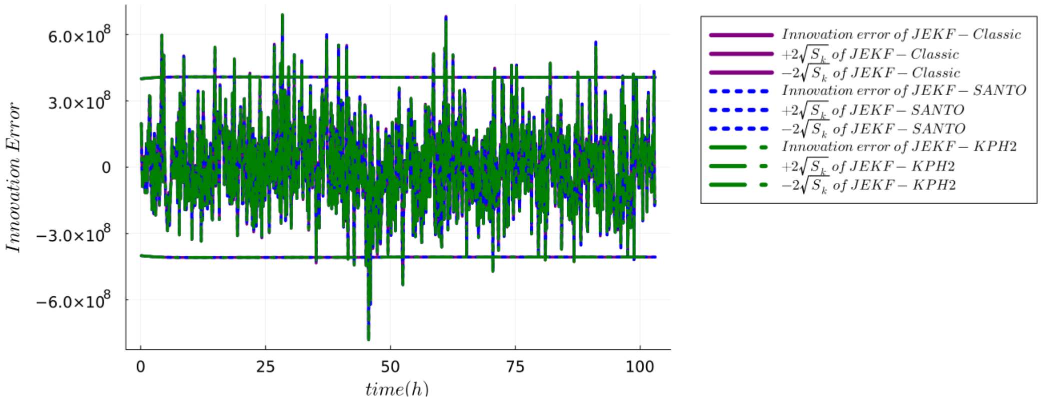

Figure S1. Innovation Magnitude Bound Test using the run B of Synthetic dataset for the NSEs with MRDE-PU and specific **Q** and **P**(0).

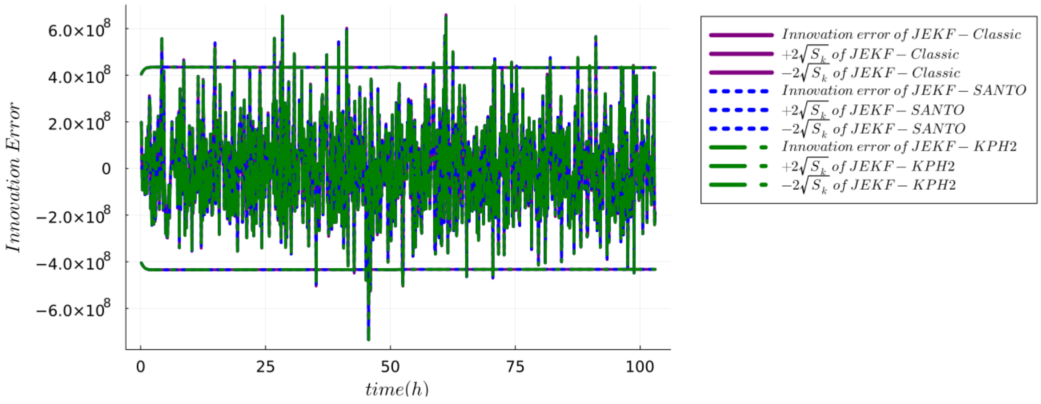

Figure S2. Innovation Magnitude Bound Test using the run B of Synthetic dataset for the NSEs with MRDE-PC and standard **Q** and **P**(0).

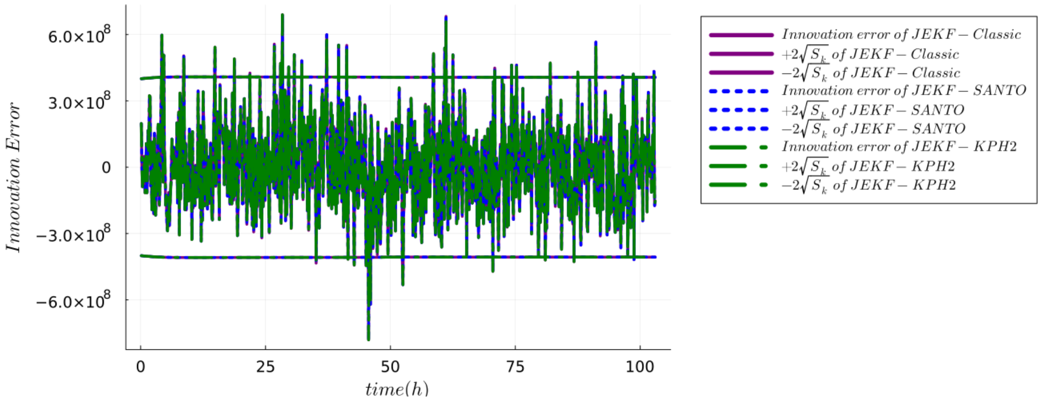

Figure S3. Innovation Magnitude Bound Test using the run B of Synthetic dataset for the NSEs with MRDE-PU and standard **Q** and **P**(0).

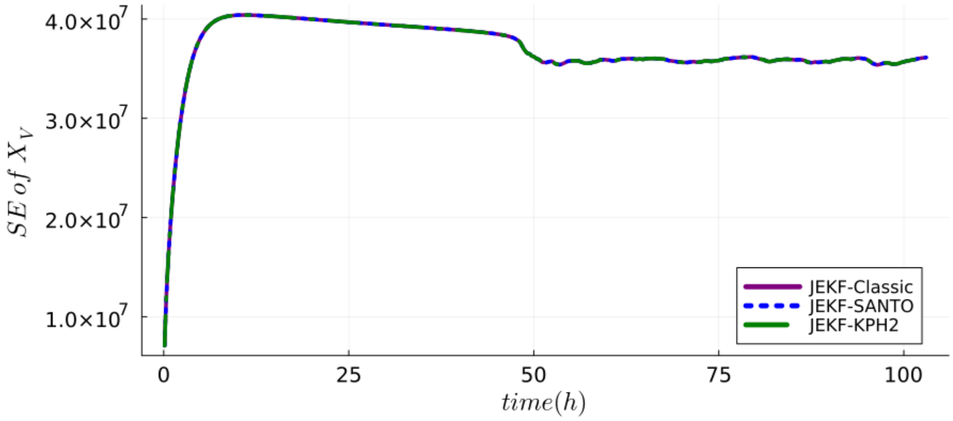

Figure S4. Standard Error of X_V at each k estimated by NSEs with Synthetic Dataset using MRDE-PU and specific P(0).

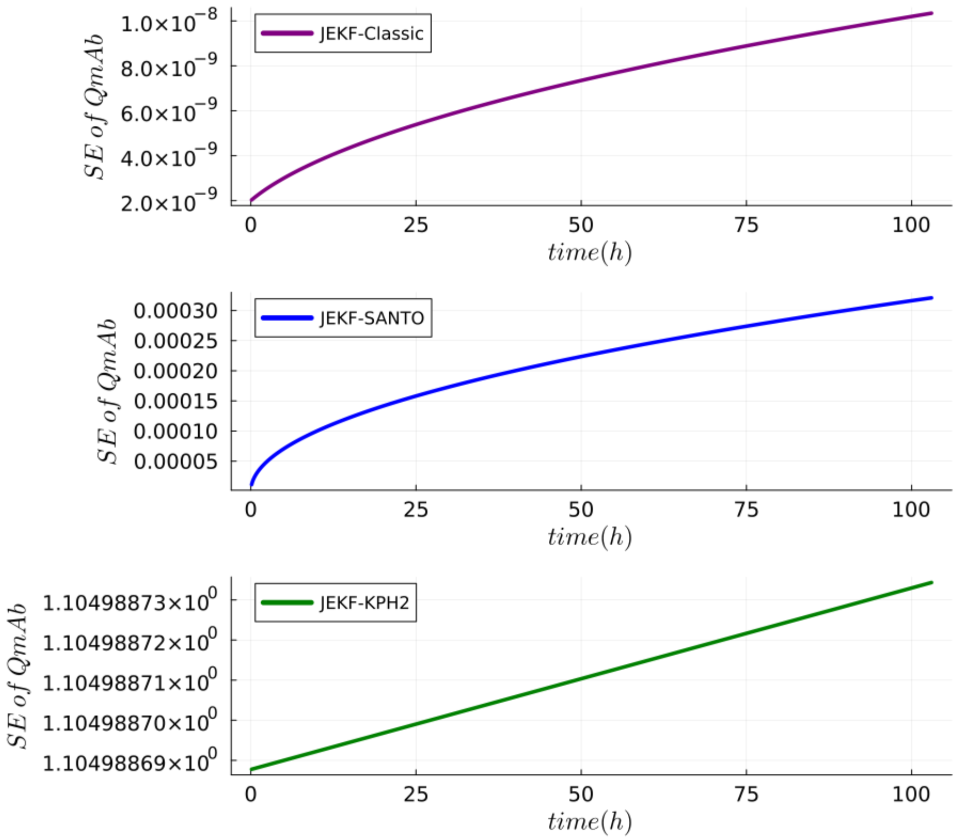

Figure S5. Standard Error of $QmAb$ at each k estimated by NSEs with Synthetic Dataset using MRDE-PC and specific P(0).

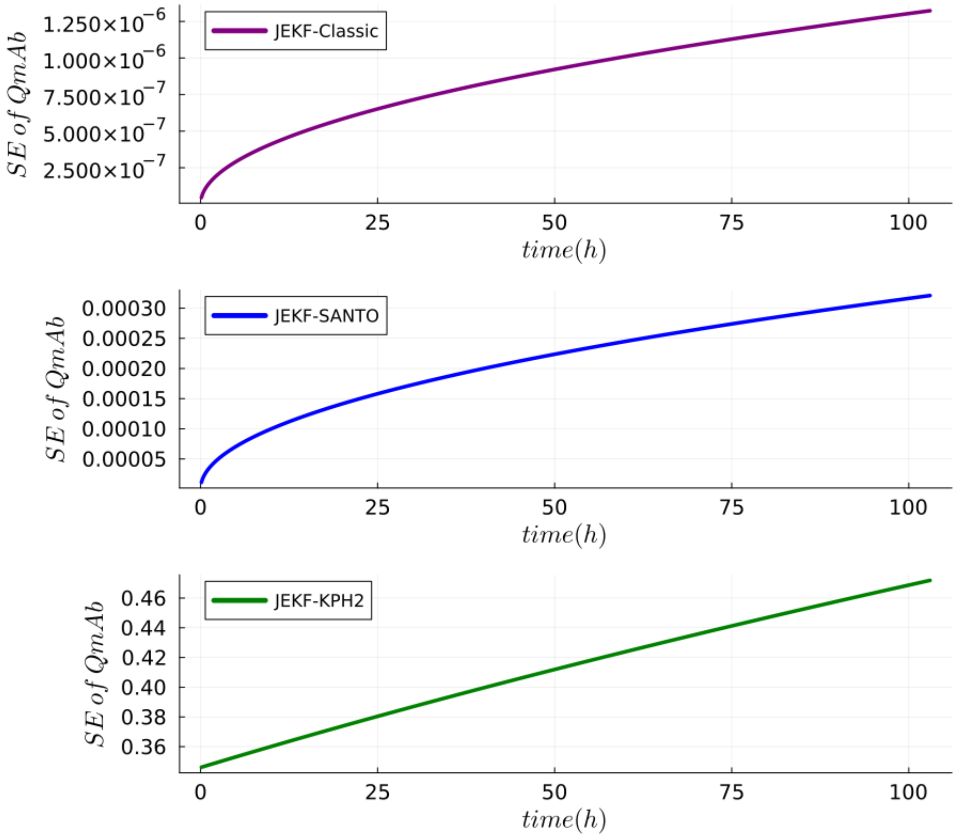

Figure S6. Standard Error of $QmAb$ at each k estimated by NSEs with Synthetic Dataset using MRDE-PU and specific P(0).

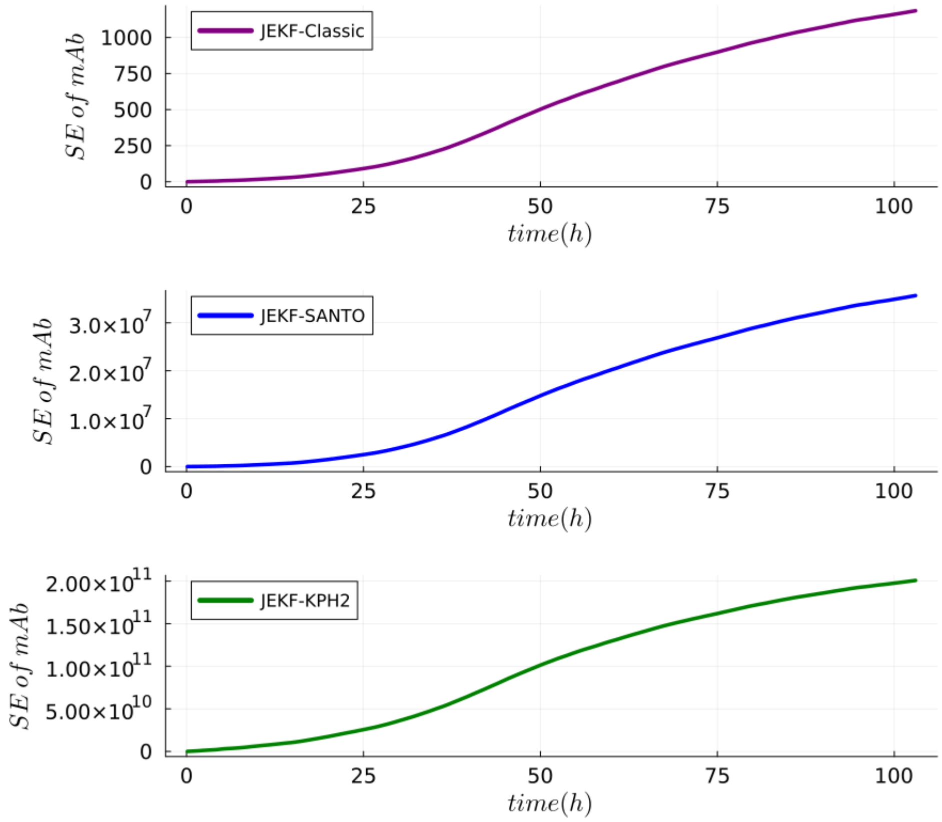

Figure S7. Standard Error of mAb at each k estimated by NSEs with Synthetic Dataset using MRDE-PC and specific P(0).

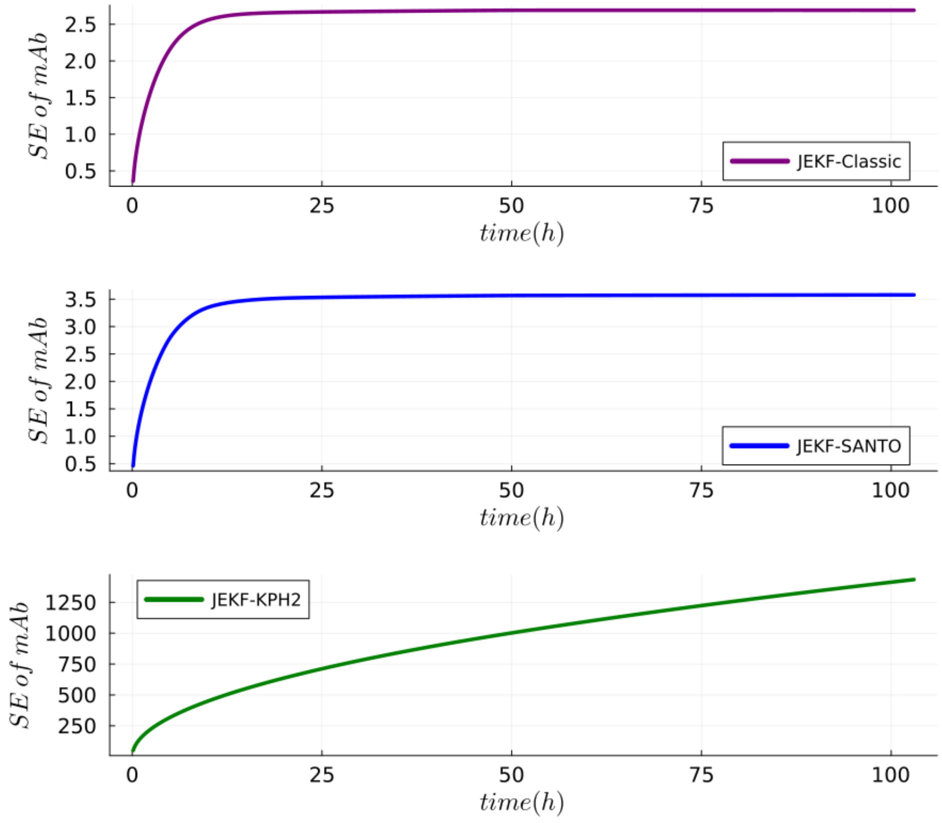

Figure S8. Standard Error of mAb at each k estimated by NSEs with Synthetic Dataset using MRDE-PU and specific P(0).

7.4. NSEs (JEKF-SANTO and JEKF-KPH2) design to address RQ3-G2

The process model (based on UMM of Section 1.5) and joint state variable vector used by JEKF-SANTO and JEKF-KPH2 to address the RQ3-G2 are the following:

$$\boldsymbol{\psi}(t) = [X_v, GLC, GLN, LAC, AMM, AAV, \mu_{X_v}, \mu_{Glc}, \mu_{Gln}, \mu_{Lac}, \mu_{Amm}, k_{deg}, \mu_{AAV}]^T. \quad (59)$$

and

$$\frac{d\boldsymbol{\psi}(t)}{dt} = \phi(\boldsymbol{\psi}(t), t) + \boldsymbol{\omega}(t), \quad (60)$$

$$\frac{d}{dt}\begin{bmatrix} X_V \\ Glc \\ Gln \\ Lac \\ Amm \\ AAV \\ \mu_{X_v} \\ \mu_{Glc} \\ \mu_{Gln} \\ \mu_{Lac} \\ \mu_{Amm} \\ k_{deg} \\ \mu_{AAV} \end{bmatrix} = \begin{bmatrix} \mu_{X_v} X_V \\ -\mu_{Glc} X_V \\ -\mu_{Gln} X_V \\ \mu_{Lac} X_V \\ \mu_{Amm} X_V + k_{deg} Gln \\ \mu_{AAV} X_V \\ 0 \\ 0 \\ 0 \\ 0 \\ 0 \\ 0 \\ 0 \end{bmatrix} + \boldsymbol{\omega}(t). \quad (61)$$

The specific **P**(t=0) that was used by the NSEs to address the RQ3-G2 are in Table S10. The specific **P**(t=0) for JEKF-KPH2 with MRDE-PC and specific **Q** come from article of Iglesias et al [6]. Furthermore, the **R**, and standard and specific **Q** used by the NSEs are presented in Table S11.

Table S8. Initial conditions of state variables of UMM case 1.5 for the JEKF-SANTO and JEKF-KPH2 test with run B-RD (Source [6]).

State Variable	Name	run B-RD
Xv	Viable cells	1.0011×10^6 c/mL
GLC	Glucose	26.7039 mM
GLN	Glutamine	4.0310 mM
LAC	Lactate	7.4385 mM
AMM	Ammonium	1.5678 mM
rAAV	rAAV viral titer	0 VG/mL

Table S9. Initial parameters obtained with A-RD for the JEKF-SANTO and JEKF-KPH2 test with run B-RD (Source [6]).

State Variable	run B-RD
μ_{Xv} (h^{-1})	0.006
μ_{GLC} (mmol 10^{-6}c h^{-1})	1.01e-7
μ_{GLN} (mmol 10^{-6}c h^{-1})	2.12e-8
μ_{LAC} (mmol 10^{-6}c h^{-1})	2.58571e-8
μ_{AMM} (mmol 10^{-6}c h^{-1})	1.47905e-9
k_{deg} (h^{-1})	0.0014591
μ_{AAV} (10^9 vg/mL h 10^6c)	65.6

Table S10. Specific initial state error covariance matrix (specific **P**(t=0)) for for the JEKF-Classic, JEKF-SANTO and JEKF-KPH2 with Real Dataset (run B) using MRDE-PC.

Parameter	Initial error Covariance	JEKF-Classic	JEKF-SANTO	JEKF-KPH2
$\mathrm{P_{X_v,X_v}}$ $(\mathrm{c^2/mL^2})$	$Cov(X_v, X_v)$	0.00	0.00	0.00
$\mathrm{P_{GLC,GLC}}$ $(\mathrm{mM^2})$	$Cov(GLC, GLC)$	0.00	0.00	0.00
$\mathrm{P_{GLN,GLN}}$ $(\mathrm{mM^2})$	$Cov(GLN, GLN)$	0.00	0.00	0.00
$\mathrm{P_{LAC,LAC}}$ $(\mathrm{mM^2})$	$Cov(LAC, LAC)$	0.00	0.00	0.00
$\mathrm{P_{AMM,AMM}}$ $(\mathrm{mM^2})$	$Cov(AMM, AMM)$	0.00	0.00	0.00
$\mathrm{P_{rAAV,rAAV}}$ $(\mathrm{VG^2/mL^2})$	$Cov(rAAV, rAAV)$	0.00	0.00	0.00
$P_{\mu_{Xv},\mu_{Xv}}$ (h^{-2})	$Cov(\mu_{Xv}, \mu_{Xv})$	1.77×10^{-9}	1.77×10^{-9}	1.77×10^{-9}
$P_{\mu_{GLC},\mu_{GLC}}$ $(mmol\ 10^{-12}c\ h^{-2})$	$Cov(\mu_{GLC}, \mu_{GLC})$	4.76×10^{-5}	4.76×10^{-5}	10.66×10^{-6}
$P_{\mu_{GLN},\mu_{GLN}}$ $(mmol\ 10^{-12}c\ h^{-2})$	$Cov(\mu_{GLN}, \mu_{GLN})$	1.05×10^{-5}	1.05×10^{-5}	1.05×10^{-5}
$P_{\mu_{LAC},\mu_{LAC}}$ $(mmol\ 10^{-12}c\ h^{-2})$	$Cov(\mu_{LAC}, \mu_{LAC})$	35.59×10^{-7}	35.59×10^{-7}	550.59×10^{-8}
$P_{\mu_{AMM},\mu_{AMM}}$ $(mmol\ 10^{-12}c\ h^{-2})$	$Cov(\mu_{AMM}, \mu_{AMM})$	6.71×10^{-10}	6.71×10^{-10}	6.71×10^{-10}
$P_{k_{deg},k_{deg}}$ (h^{-2})	$Cov(k_{deg}, k_{deg})$	8.71×10^{-8}	8.71×10^{-8}	8.71×10^{-8}
$P_{\mu_{rAAV},\mu_{rAAV}}$ $(vg^2/mL^2\ h^2\ 10^{12}c)$	$Cov(\mu_{rAAV}, \mu_{rAAV})$	42000	42000	10000
$P_{X_v,\mu_{GLC}}$ $(c/mL)(mM)$	$Cov(X_v, \mu_{GLC})$	0.00	0.000006751	0.00
$P_{X_v,\mu_{LAC}}$ $(c/mL)(mM)$	$Cov(X_v, \mu_{LAC})$	0.00	0.0000072505	0.00
$P_{X_v,\mu_{rAAV}}$ $(c/mL)(VG/mL)$	$Cov(X_v, \mu_{rAAV})$	0.00	9500.3625	0.00

Table S11. Measurement noise variance **R**, and error covariance matrix of process model $\mathbf{Q}_{i,i}$ for the JEKF-Classic, JEKF-SANTO and JEKF-KPH2 with Real Dataset (run B) using MRDE-PC.

**Parameter**	**Name**	**JEKF-Classic**	**JEKF-SANTO**	**JEKF-KPH2**
R (c^2/mL^2)	Viable cells MNV [1]	130×10^5	130×10^5	130×10^5
Q_{X_v,X_v} (c^2/mL^2)	Viable cells PNV [2]	50×10^5	50×10^5	50×10^5
$Q_{GLC,GLC}$ (mM^2)	Glucose PNV	0.0006	0.0006	0.0006
$Q_{GLN,GLN}$ mM^2	Glutamine PNV	0.0006	0.0006	0.0006
$Q_{LAC,LAC}$ (mM^2)	Lactate PNV	0.0006	0.0006	0.0006
$Q_{AMM,AMM}$ (mM^2)	Ammonium PNV	0.0006	0.0006	0.0006
$Q_{rAAV,rAAV}$ (VG^2/mL^2)	AAV viral titer PNV	0.0006	0.0006	0.0006
$Q_{\mu_{X_V},\mu_{X_V}}$ (h^{-2})	μ_{X_V} PNV	1.77×10^{-7}	1.77×10^{-7}	1.77×10^{-7}
$Q_{\mu_{GLC},\mu_{GLC}}$ $(mmol\ 10^{-12}c\ h^{-2})$	μ_{GLC} PNV	0.21×10^{-16}	0.21×10^{-16}	0.21×10^{-16}
$Q_{\mu_{GLN},\mu_{GLN}}$ $(mmol\ 10^{-12}c\ h^{-2})$	μ_{GLN} PNV	7.86×10^{-18}	7.86×10^{-18}	7.86×10^{-18}
$Q_{\mu_{LAC},\mu_{LAC}}$ $(mmol\ 10^{-12}c\ h^{-2})$	μ_{LAC} PNV	13.59×10^{-7}	13.59×10^{-7}	13.59×10^{-7}
$Q_{\mu_{AMM},\mu_{AMM}}$ $(mmol\ 10^{-12}c\ h^{-2})$	μ_{AMM} PNV	0.11×10^{-8}	0.11×10^{-8}	0.11×10^{-8}
$Q_{k_{deg},k_{deg}}$ (h^{-2})	k_{deg} PNV	0.71×10^{-8}	0.71×10^{-8}	0.71×10^{-8}
$Q_{\mu_{rAAV},\mu_{rAAV}}$ $(vg^2/mL^2\ h^2\ 10^{12}c)$	μ_{rAAV} PNV	0.31	0.31	0.31

[1] MNV—measurement noise value; [2] PNV—process noise value.

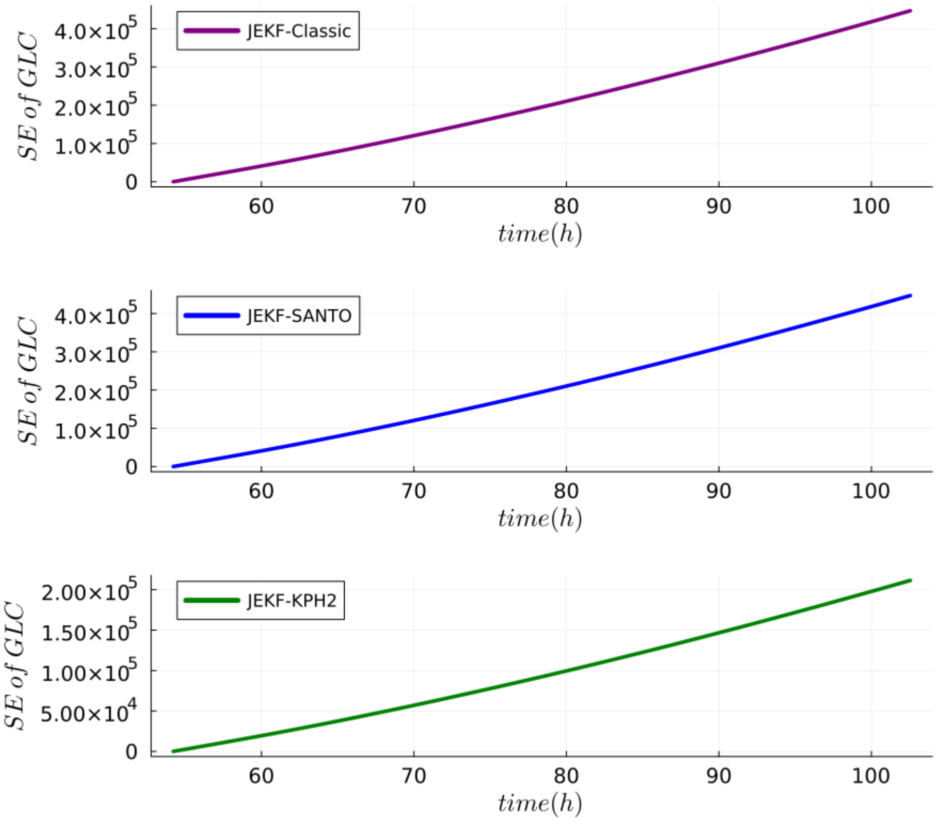

Figure S9. Standard Error of *GLC* at each k estimated by NSEs with Real Dataset using MRDE-PC and specific P(0).

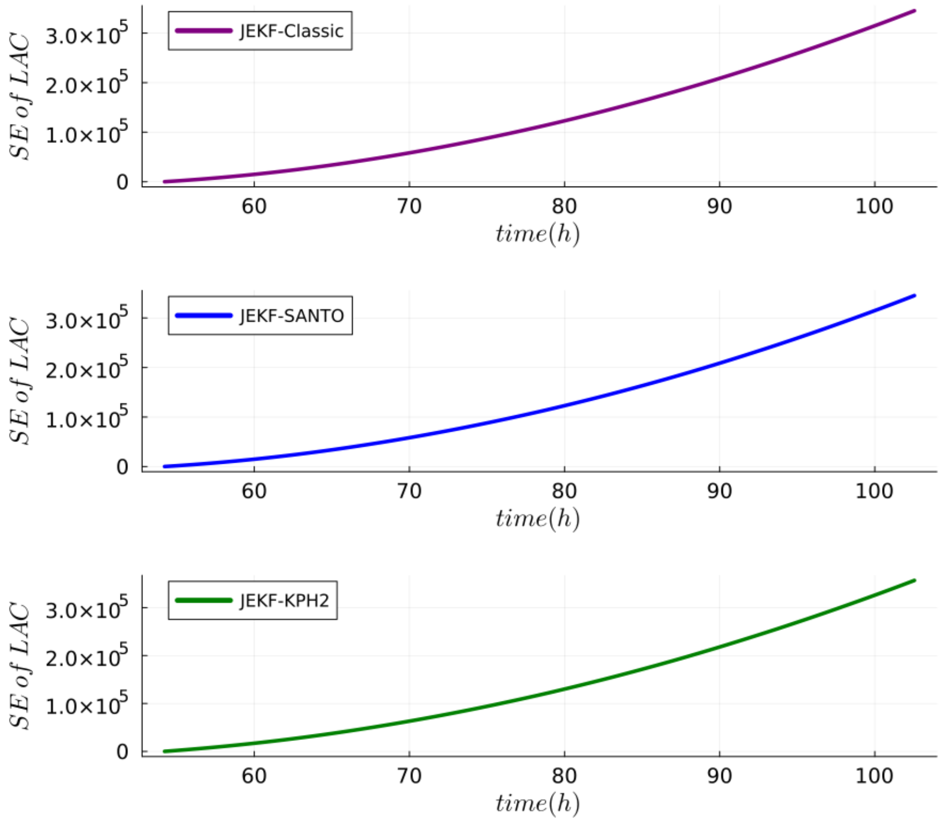

Figure S10. Standard Error of *LAC* at each k estimated by NSEs with Real Dataset using MRDE-PC and specific P(0).

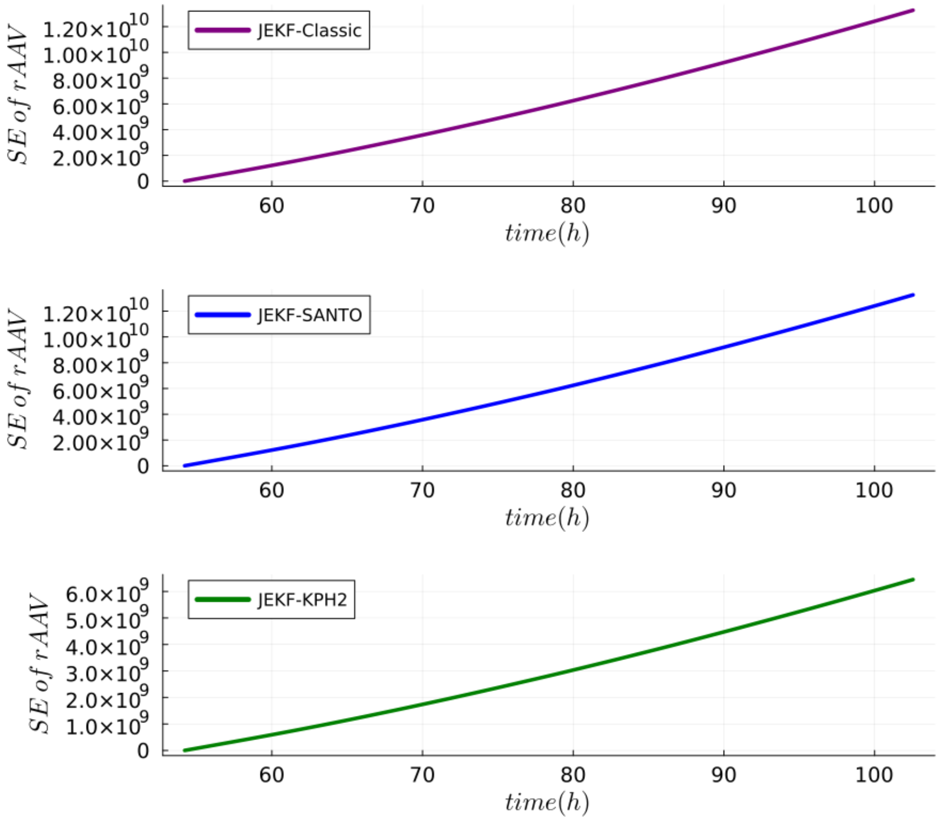

Figure S11. Standard Error of $rAAV$ at each k estimated by NSEs with Real Dataset using MRDE-PC and specific P(0).

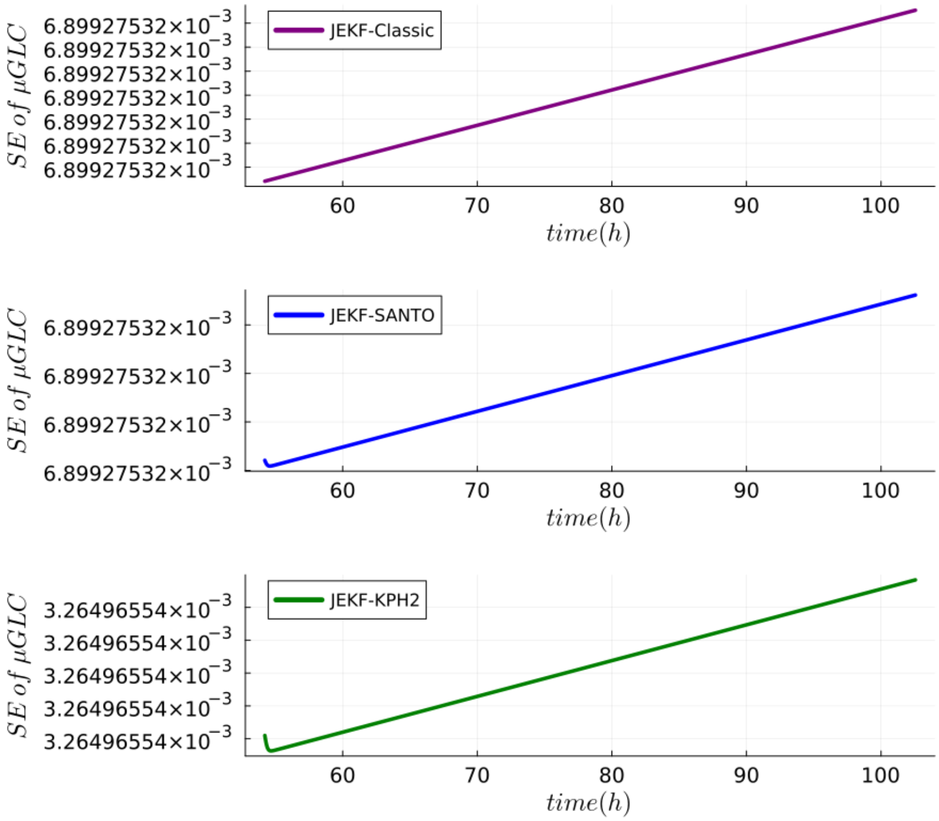

Figure S12. Standard Error of μGLC at each k estimated by NSEs with Real Dataset using MRDE-PC and specific P(0).

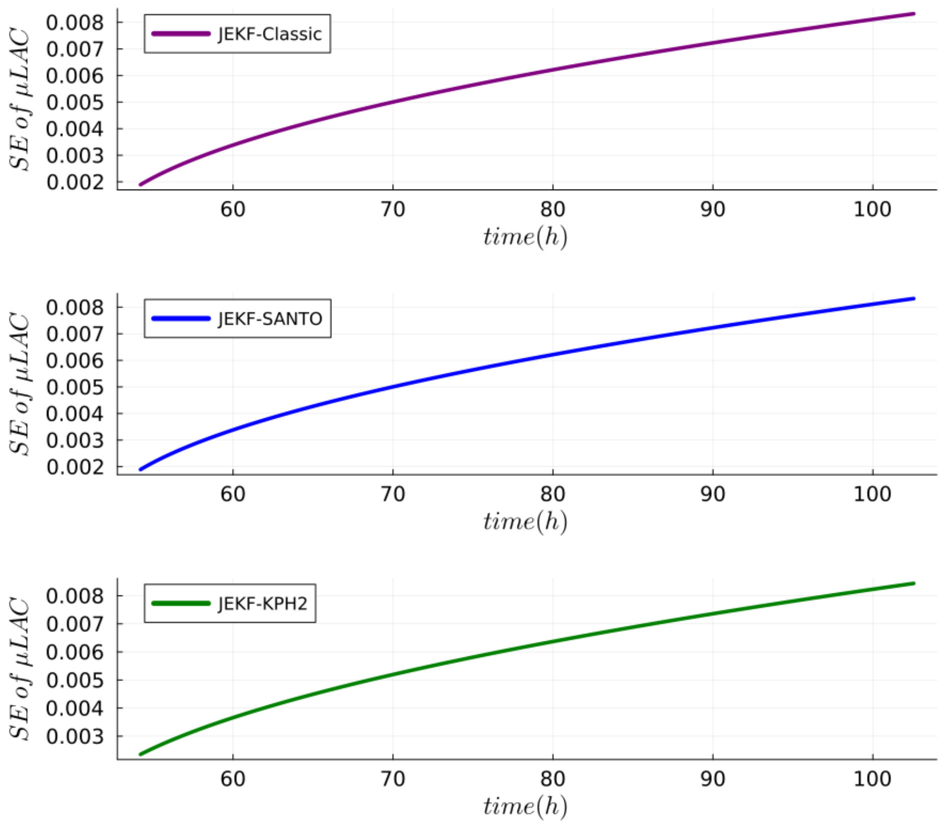

Figure S13. Standard Error of μLAC at each k estimated by NSEs with Real Dataset using MRDE-PC and specific P(0).

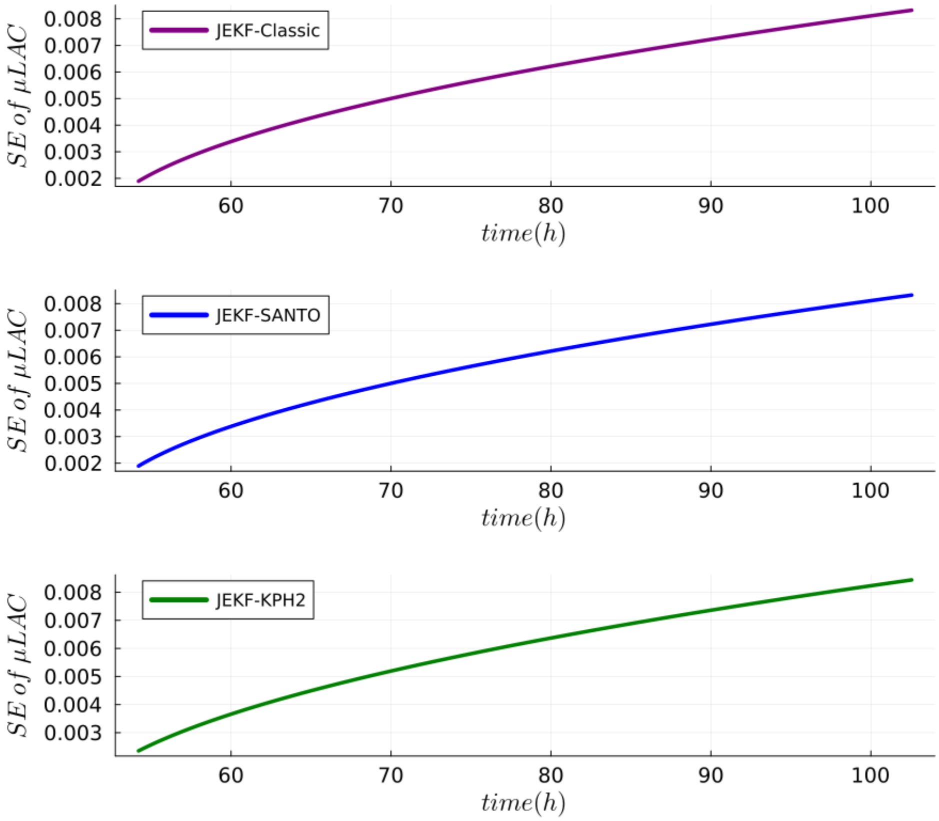

Figure S14. Standard Error of $\mu rAAV$ at each k estimated by NSEs with Real Dataset using MRDE-PC and specific P(0).

7.5. Results with synthetic dataset

In Figures S15, and S16, we can see the estimations of JEKF-SANTO and JEKF-KPH2 with run B-SD with standard **P**(t=0). This show that they are sensible to **P**(t=0) because the best results were obtained with specific **P**(t=0) (results in main text).

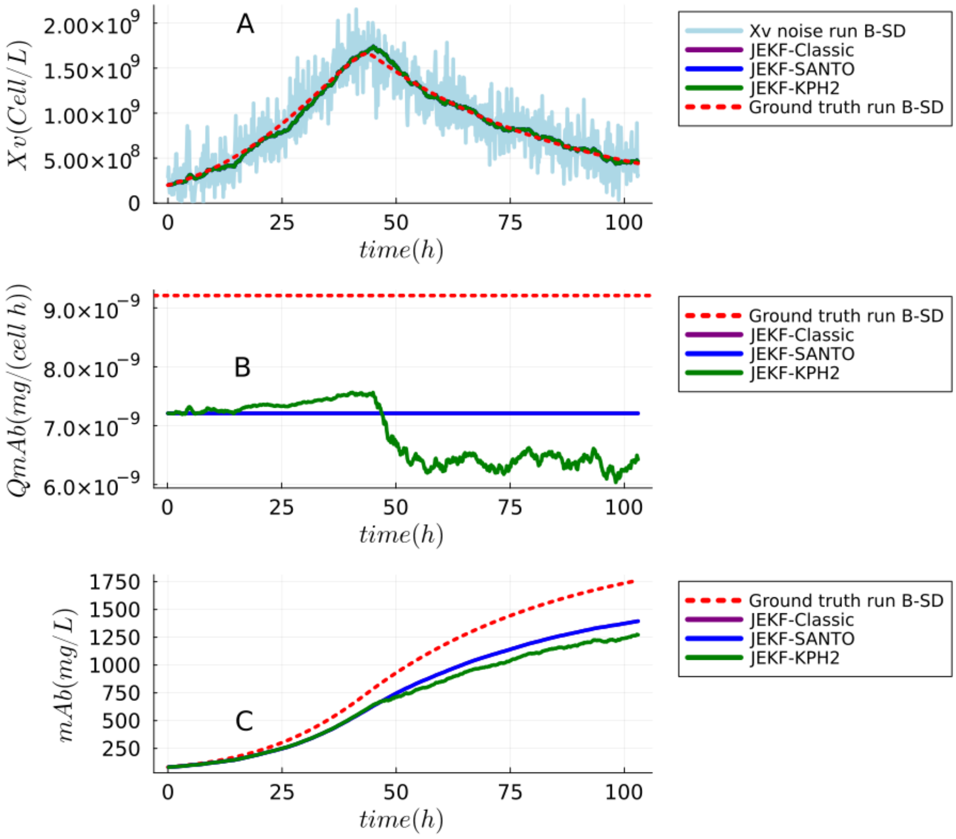

Figure S15. The JEKF-SANTO and JEKF-KPH2 avoid the JEKF failure in B-SD, but they need an specific $P(t=0)$. First, plot A shows the estimations regards Xv, and all estimations were close the ground truth. The plots B and C show the estimations regards the unshared parameter QmAb and mAb (titer) far from the ground truth, respectively. The NSEs were executed with **MRDE-PC** and **standard** $P(t=0)$.

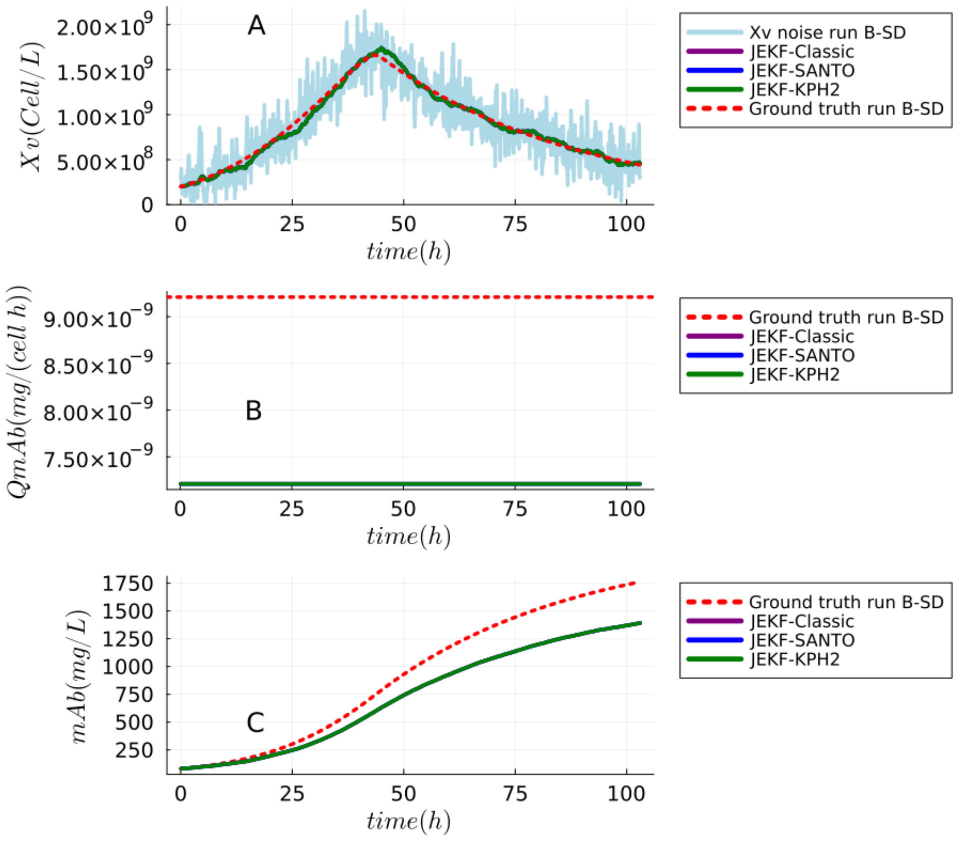

Figure S16. The JEKF-SANTO and JEKF-KPH2 avoid the JEKF failure in B-SD, but they need an specific $P(t = 0)$. First, plot A shows the estimations regards Xv, and all estimations were close the ground truth. The plots B and C show the estimations regards the unshared parameter QmAb and mAb (titer) far from the ground truth, respectively. The NSEs were executed with **MRDE-PU** and **standard** $P(t = 0)$.

Table S12. RMSPE between NSEs estimations about mAb and ground truth of run B in synthetic dataset with standard **P**(t=0).

NSE	RMSPE (MRDE-PU)	RMSPE (MRDE-PC)
JEKF-SANTO	18.80%	18.65%
JEKF-KPH2	18.80%	18.65%
JEKF-Classic	18.80%	18.65%

7.6. Codes and datasets availability

All source codes and datasets used in this work can be found in GitHub: https://github.com/cristovaoiglesias/JEKF-SANTO.

References

1. Goudar, C.T. Computer programs for modeling mammalian cell batch and fed-batch cultures using logistic equations. *Cytotechnology* **2012**, *64*, 465–475.
2. Narayanan, H.; Behle, L.; Luna, M.F.; Sokolov, M.; Guillén-Gosálbez, G.; Morbidelli, M.; Butté, A. Hybrid-EKF: Hybrid Model coupled with Extended Kalman Filter for real-time monitoring and control of mammalian cell culture. *Biotechnology and Bioengineering* **2020**, *117*, 2703–2714.
3. Ohadi, K.; Legge, R.L.; Budman, H.M. Development of a soft-sensor based on multi-wavelength fluorescence spectroscopy and a dynamic metabolic model for monitoring mammalian cell cultures. *Biotechnology and bioengineering* **2015**, *112*, 197–208.
4. Ji, Z.; Brown, M. Joint state and parameter estimation for biochemical dynamic pathways with iterative extended Kalman filter: comparison with dual state and parameter estimation. *The Open Automation and Control Systems Journal* **2009**, *2*.
5. Liu, Y.; Gunawan, R. Bioprocess optimization under uncertainty using ensemble modeling. *Journal of biotechnology* **2017**, *244*, 34–44.
6. Iglesias Jr, C.F.; Xu, X.; Mehta, V.; Akassou, M.; Venereo-Sanchez, A.; Belacel, N.; Kamen, A.; Bolic, M. Monitoring the Recombinant Adeno-Associated Virus Production using Extended Kalman Filter. *Processes* **2022**, *10*, 2180.
7. Jazwinski, A. Stochastic processes and filtering theory. *Mathematics in science and engineering (* **1970**.
8. Frogerais, P.; Bellanger, J.J.; Senhadji, L. Various ways to compute the continuous-discrete extended Kalman filter. *IEEE Transactions on Automatic Control* **2011**, *57*, 1000–1004.
9. Kulikov, G.Y.; Kulikova, M.V. Accurate numerical implementation of the continuous-discrete extended Kalman filter. *IEEE Transactions on Automatic Control* **2013**, *59*, 273–279.
10. Albiol, J.; Robusté, J.; Casas, C.; Poch, M. Biomass estimation in plant cell cultures using an extended Kalman filter. *Biotechnology progress* **1993**, *9*, 174–178.
11. Yousefi-Darani, A.; Paquet-Durand, O.; Hitzmann, B. The Kalman filter for the supervision of cultivation processes. *Digital Twins* **2020**, pp. 95–125.
12. Paquet-Durand, O.; Zettel, V.; Yousefi-Darani, A.; Hitzmann, B. The supervision of dough fermentation using image analysis complemented by a continuous discrete extended Kalman filter. *Processes* **2020**, *8*, 1669.
13. Jin, X.B.; Robert Jeremiah, R.J.; Su, T.L.; Bai, Y.T.; Kong, J.L. The new trend of state estimation: from model-driven to hybrid-driven methods. *Sensors* **2021**, *21*, 2085.
14. Zhang, D.; Del Rio-Chanona, E.A.; Petsagkourakis, P.; Wagner, J. Hybrid physics-based and data-driven modeling for bioprocess online simulation and optimization. *Biotechnology and bioengineering* **2019**, *116*, 2919–2930.
15. Brockwell, P. Time series analysis. *Encyclopedia of Statistics in Behavioral Science* **2005**.
16. Haykin, S.S.; Haykin, S.S. *Kalman filtering and neural networks*; Vol. 284, Wiley Online Library, 2001.
17. Särkkä, S. Bayesian estimation of time-varying systems: Discrete-time systems. *Written material for the course held in Spring* **2012**, *2012*.
18. Murphy, K.P. *Machine learning: a probabilistic perspective*; MIT press, 2012.
19. Assimakis, N.; Adam, M. Kalman filter Riccati equation for the prediction, estimation, and smoothing error covariance matrices. *International Scholarly Research Notices* **2013**, *2013*.
20. Kulikova, M.V.; Kulikov, G.Y. Adaptive ODE solvers in extended Kalman filtering algorithms. *Journal of Computational and Applied Mathematics* **2014**, *262*, 205–216.
21. Fraser, C.T. Adaptive extended Kalman filtering strategies for autonomous relative navigation of formation flying spacecraft. PhD thesis, Carleton University, 2019.
22. Labbe Jr, R.R. Kalman and Bayesian Filters in Python **2020**.
23. Benhamou, E. Kalman filter demystified: from intuition to probabilistic graphical model to real case in financial markets. *arXiv preprint arXiv:1811.11618* **2018**.
24. Yousefi-Darani, A.; Paquet-Durand, O.; Hitzmann, B., The Kalman Filter for the Supervision of Cultivation Processes. In *Digital Twins: Applications to the Design and Optimization of Bioprocesses*; Herwig, C.; Pörtner, R.; Möller, J., Eds.; Springer International Publishing: Cham, 2021; pp. 95–125. https://doi.org/10.1007/10_2020_145.
